# Serum miR-1290 and miR-1246 as Potential Diagnostic Biomarkers of Human Pancreatic Cancer

**DOI:** 10.7150/jca.38048

**Published:** 2020-01-01

**Authors:** Jia Wei, Lu Yang, Yi-ning Wu, Jian Xu

**Affiliations:** 1 Department of Laboratory Medicine, The First Affiliated Hospital of Nanjing Medical University, Nanjing 210029, China.; 2 National Key Clinical Department of Laboratory Medicine, Nanjing 210029, China.

**Keywords:** serum miRNA, pancreatic cancer, diagnostic biomarker

## Abstract

**Background**: Pancreatic cancer (PC) is a highly malignant tumor with no effective early diagnostic biomarkers. This study was performed to screen and identify serum microRNAs (miRNAs) as noninvasive biomarkers for PC diagnosis.

**Methods**: Two upregulated miRNAs were selected by integrated analysis of three independent GEO datasets. Then, the expressions of two miRNAs in serum were determined by quantitative reverse-transcription PCR among 120 PC patients, 40 benign disease controls and 40 healthy controls. The correlation between serum miRNAs and clinical characteristics was analyzed. The diagnostic utility of miRNAs was compared to CA19-9 using receiver operating characteristic curve analysis.

**Results**: We discovered miR-1290 and miR-1246 were upregulated in PC patients through GEO datasets analysis. Serum miR-1290 and miR-1246 expression levels were elevated in PC patients compared to all controls and dramatically decreased after tumor resection (all P<0.001). The area under the curve (AUC) for miR-1290 was larger than miR-1246 and CA19-9 (miR-1290: 0.91; miR-1246: 0.81; CA19-9: 0.82). The combined diagnosis of individual or both miRNAs with CA19-9 was more effective for discriminating PC from all controls than the single CA19-9 assay (miR-1290+CA19-9: 0.96, miR-1246+CA19-9: 0.93, miR-1290+miR-1246+CA19-9: 0.97). The abundance of serum miR-1290 and miR-1246 was associated with tumor stage and size respectively and logistic modeling proved that both of them were independent risk factors for PC.

**Conclusion**: Serum miR-1290 and miR-1246 might be promising biomarkers for PC diagnosis and the combined detection of CA19-9, together with miR-1290 or miR-1246, could improve the diagnostic accuracy of PC.

## Introduction

Pancreatic cancer (PC) is considered one of the most malignant cancers, being the fourth leading cause of cancer deaths worldwide 1. Most patients are diagnosed at advanced stages because of the lack of early clinical manifestations and occult onset. In addition, pancreatic tumors are typically resistant to radiotherapy and chemotherapy, leading to a very low survival rate of approximately 8% at 5 years and high mortality 1-2. The health burden of PC in China is continuously increasing, with annual mortality rates (79.4 per 100,000 people) almost to the same degree with the incidence rates (90.1 per 100,000 people) 3.

Serum carbohydrate antigen 19-9(CA19-9) is the only clinically available Food and Drug Administration (FDA) approved biomarker for PC, displaying a mean sensitivity of 79-81% and a mean specificity of 70-92% for PC diagnosis 4. However, the CA19-9 is also elevated in other forms of digestive tract cancer and some non-cancerous conditions 4. Although endoscopic ultrasound-guided fine needle aspiration (EUS-FNA) has become the preferred modality for obtaining pathologic confirmation, it is limited by the invasive procedures and technical difficulty 5. Therefore, the exploration of novel non-invasive biomarkers with high sensitivity and specificity for PC diagnosis is urgently needed.

MicroRNAs (miRNAs) are small noncoding RNAs with the length ranging from 18 to 25 nucleotides and regulate physical and pathological processes such as cell proliferation, protein metabolism, and tumor development 6. A large body of evidence has suggested that miRNAs could function as tumor suppressor genes or oncogenes, indicating a potential target in cancer diagnosis and treatments 7-8. Moreover, they are highly conserved, tissue-specific and keep stable in body fluids, which make it possible to monitor diseases through miRNA analysis 9. It was reported that the upregulation of miR-21-5p, miR-193a-3p, miR-221-3p, miR-99 and miR-155 was associated with initiation and progression of PC, as well as resistance to chemotherapy 10-14, whereas they were not always of high diagnostic value and further studies are required to identify promising miRNA signatures in blood circulation for PC diagnosis.

In this study, we screened two upregulated miRNAs (miR-1290 and miR-1246) in the serum of PC patients by conducting an integrated analysis of three GEO datasets. Thus, we aimed to explore the potential of candidate miRNAs for PC diagnosis.

## Method and Materials

### Patients and samples

Serum samples were obtained from 120 PC patients, 40 benign pancreatic disease controls (DC) (10 cases with chronic pancreatitis, 15 cases with intraductal papillary mucinous neoplasm and 15 cases with pancreatic neuroendocrine tumor) and 40 healthy controls (HC) at the First Affiliated Hospital of Nanjing Medical University between January 2016 and December 2017. These samples were collected before any therapeutic procedures, including surgery, chemotherapy, and radiotherapy. Patients with any cancer history other than PC were excluded. Serum samples were extracted from whole blood after centrifugation (2800g, 10 min) and stored at -80 ℃ until miRNA extraction.

Clinicopathological factors and clinical stage were classified according to the 8th edition of International Union Against Cancer (UICC) tumor-node-metastasis (TMN) system. Clinical data including patient age, gender, tumor size and location, lymph node metastasis, differentiation degrees, clinical and histological grade, diabetes history, tobacco and alcohol addiction, CA19-9, serum glucose (Glu) and triglyceride (TG) levels was recorded. This study was approved by the Research and Ethical Committee of the First Affiliated Hospital of Nanjing Medical University.

### Study design

To explore the dysregulated miRNAs in PC, microarray datasets of the serum samples were obtained from Gene Expression Omnibus (GEO) database. We carried out a comprehensive analysis of miRNA expression profiles in GSE113486 and GSE106817. The selected miRNAs were investigated in GSE55139. Then, the expression of candidate miRNAs was verified by quantitative reverse-transcription PCR (qRT-PCR) in the serum samples mentioned above. Besides, the dynamic changes of serum miR-1290 and miR-1246 were measured in 34 paired pre- and post-operative serum samples. Postoperative samples were obtained 1 week after pancreatectomy.

### miRNA Extraction and qRT-PCR

MiRNA was isolated from 200μL serum by the miRcute miRNA Isolation Kit (Tiangen, Beijing, China), according to the manufacturer protocol. The resulting RNA pellet was dissolved in 20μL RNase‐free water and stored at -80°C until further analysis.

QRT-PCR for miR-1290 and miR-1246 was performed using Hairpin-it^TM^ microRNA RT-PCR Quantitation Kit (GenePharma, Shanghai, China) according to the manufacturer's instructions. The amplification reactions were initiated with denaturation at 95℃ for 3min, followed by 40 cycles of 95℃ for 12s and 62℃ for 40s.

We also calculated the absolute miRNA concentrations by conducting calibration curves developed with the synthetic mature miRNA oligonucleotides (miR1290: 5'-UGGAUUUUUGGAUCAGGGA-3'; miR1246: 5'-AAUGGAUUUUUGGAGCAGG-3'). For each assay, 4-fold serial dilution of synthetic miRNA from 10^2^ fmol/L to 10^5^ fmol/L was used to generate the standard curves. The resulting CT values were plotted versus the log_10_ of the amount of synthetic miRNAs. Each point represented the mean of three independent experiments (Supplementary Figure S1).

QRT-PCR was performed using the ABI 7500 fast real time PCR system (Applied Biosystems, Foster City, USA). The experiment was independently repeatedly for three times.

### Bioinformatics analysis of miRNAs

We presented DIANA‐miRPath v3.0 (http://www.microrna.gr/miRPathv3) to assess miRNA regulatory roles and identify the controlled pathways 15. The results of Kyoto Encyclopedia of Genes and Genomes (KEGG) and Gene Ontology (GO) analyses were shown by heat‐maps.

### Statistics

All statistical analyses were performed with SPSS version 24.0 (IBM Corp, USA) and GraphPad Prism Software (San Diego, USA). All the results were judged using two-tailed test. P<0.05 was considered statistically significant.

All the data were presented as median and interquartile range. Clinicopathological characteristics among groups were compared by chi-square test. The Wilcoxon signed-rank test was utilized to compare the paired serum samples obtained from pre- and postoperative patients. The Kruskal-Wallis test was performed to compare the differences in miRNA expressions in more than three groups. Besides, spearman correlation analysis was used to evaluate correlations between miRNAs and CA19-9 levels. Receiver-operating characteristic (ROC) curves and the area under the ROC curve (AUC) were used to assess the feasibility of using the serum miRNA expression levels or CA19-9 as diagnostic markers for discriminating PC patients from benign pancreatic disease patients and healthy individuals. Z test was then applied to assess the statistic differences among the AUCs. Univariate and multivariate logistic regression analyses were used to identify independent risk factors associated with PC. The results of the models were presented as odds ratios (OR) with 95% confidence intervals (CI) and p values.

## Results

### MiR-1290 and miR-1246 were upregulated in the GEO datasets analysis

**Figure 1:**
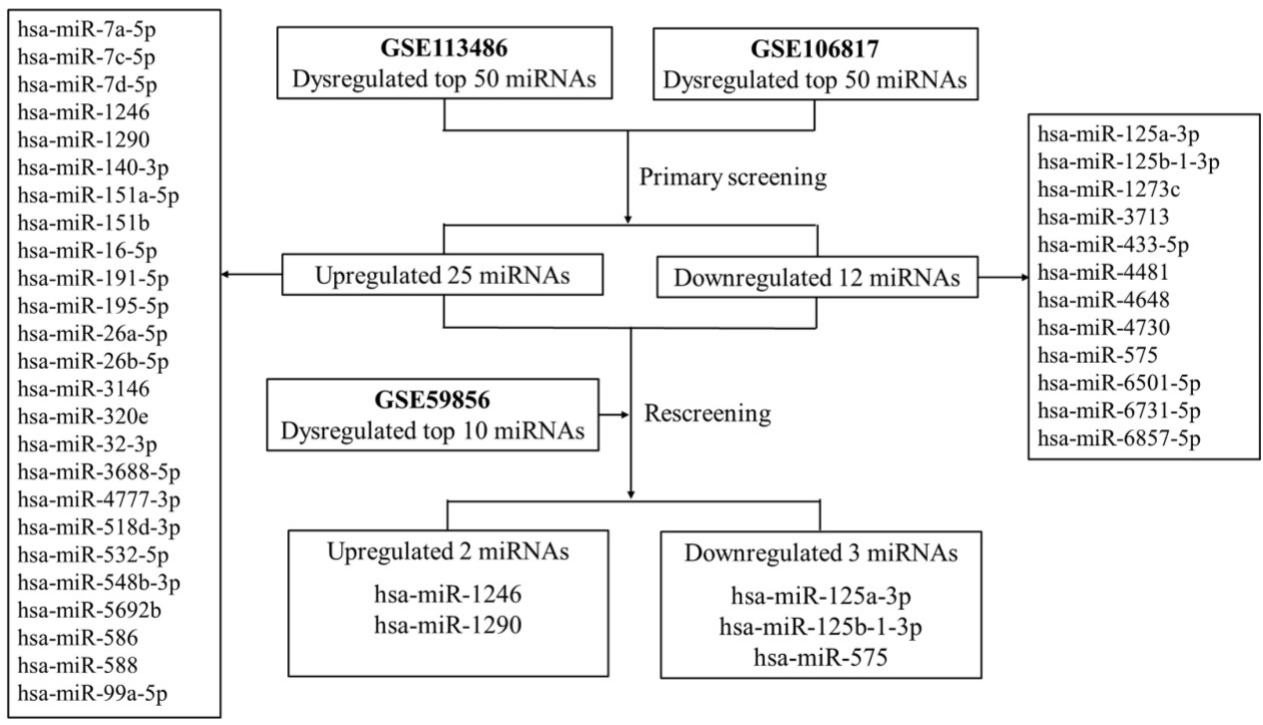
The flow diagram of discovering candidate miRNAs in GEO datasets.

First, two GEO datasets (GSE113486 and GSE106817) which conducted a comprehensive comparative analysis of serum miRNA expression profiles between pancreatic cancer and non-cancer patients were explored. We found 37 consistently dysregulated miRNAs (including 25 upregulated and 12 downregulated miRNAs). By comprehensively comparing with another GEO dataset (GSE59856), we found that only serum miR-1290 and miR-1246 were significantly upregulated in pancreatic cancer when compared with HC (fold change >5, Figure 1). Meanwhile, serum miR-125a-3p, miR-125b-1-3p and miR-575 were downregulated slightly (fold change approximately at -2, Supplementary Table S1). Therefore, we selected serum miR-1290 and miR-1246 acting as potential biomarkers for the subsequent analysis.

### Serum microRNA levels in PC patients compared to controls

**Figure 2:**
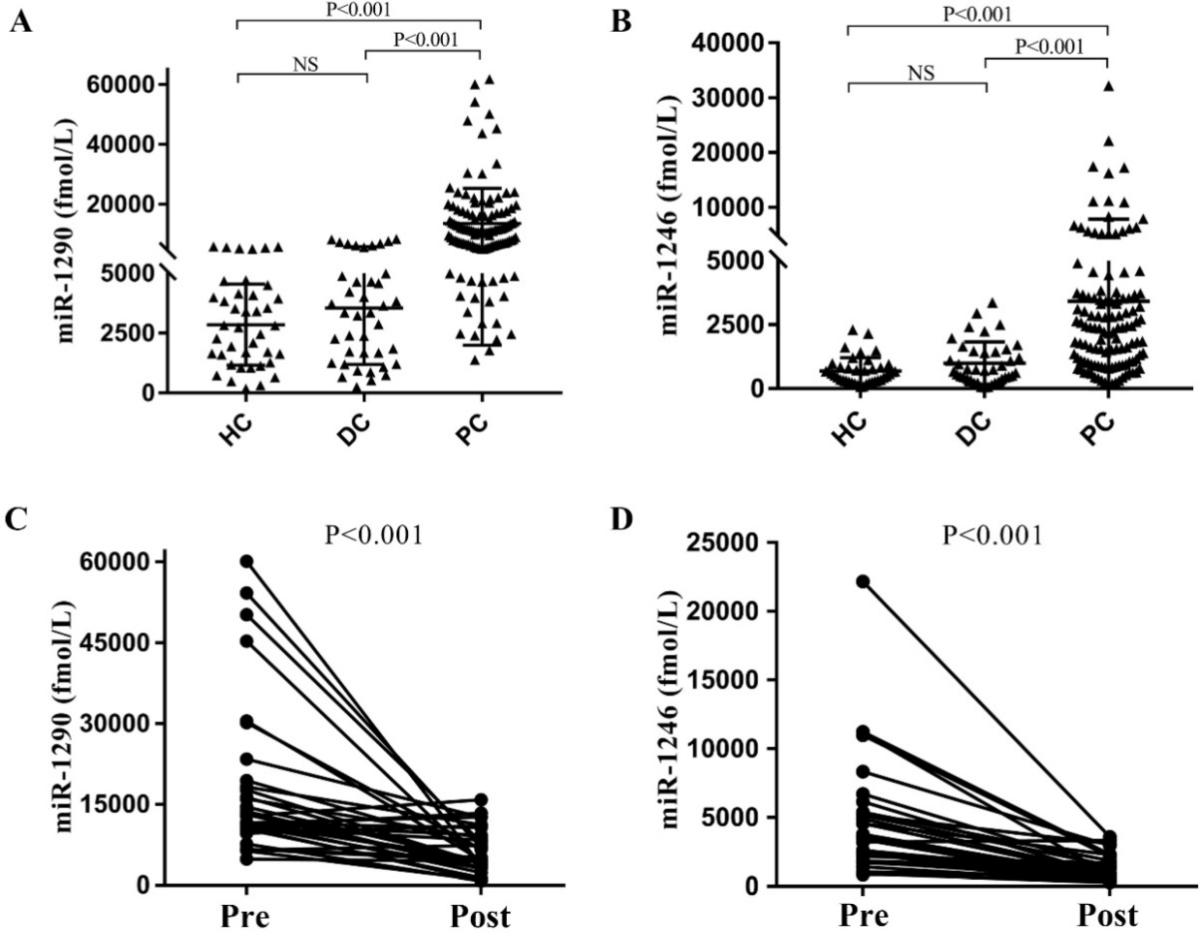
Expression levels of serum targeted miRNAs in the cohort. The absolute concentrations of serum miR-1290 and miR-1246 were detected from patients with pancreatic cancer (PC), benign pancreatic disease (DC) and healthy controls (HC) (A, B). The expression of miR-1290 and miR-1246 decreased significantly after surgical resection of malignancies (C, D).

To verify the above results from the GEO datasets analysis, we next performed qRT-PCR analysis for miR-1290 and miR-1246 in the cohort of 120 PC patients (male: 58.3%, age: 62.72±10.90), 40 DC patients (male: 40.0%, age: 56.05±11.29) and 40 HC individuals. We observed that absolute concentrations of both miRNAs were higher in PC patients' serum than the other controls (all P<0.001, Figure 2A and 2B). Median values for serum miR-1290 in PC patients, DC and HC groups were 10414.25 (5697.79, 15729.12) fmol/l, 3362.20 (1360.54, 4961.07) fmol/l, 2783.27 (1357.21, 4132.84) fmol/l respectively. Similarly, miR-1246 median levels were higher in PC than DC and HC group (1985.38 (908.62, 3710.57) fmol/l vs. 727.59 (392.26, 1464.25) fmol/l and (302.03, 841.26) fmol/l). After analysis of 34 paired pre- and post-operation serum samples of PC patients, we noted the expression of miR-1290 and miR-1246 decreased obviously after surgical resection of malignancies (all P <0.001, Figure 2C and 2D).

### Association between selected miRNAs and clinical characteristics in PC

Association between candidate miRNAs levels and clinicopathological parameters were summarized in Table 1. For the PC samples, the expression level of miR-1290 was notably elevated in male (P=0.026). Moreover, higher expression of miR-1290 was significantly more frequently observed in samples from patients at stage III and IV than those at stage I and II samples (P=0.043). In addition, we found a significant correlation between expression levels of miR-1246 and tumor size (P=0.026). However, there was no significant correlation between the abundance of the two candidate miRNAs and age, lymph node metastasis, differentiation degrees, tumor location and so on.

### Diagnostic performance of serum miR-1290, miR-1246 and CA19-9

**Figure 3:**
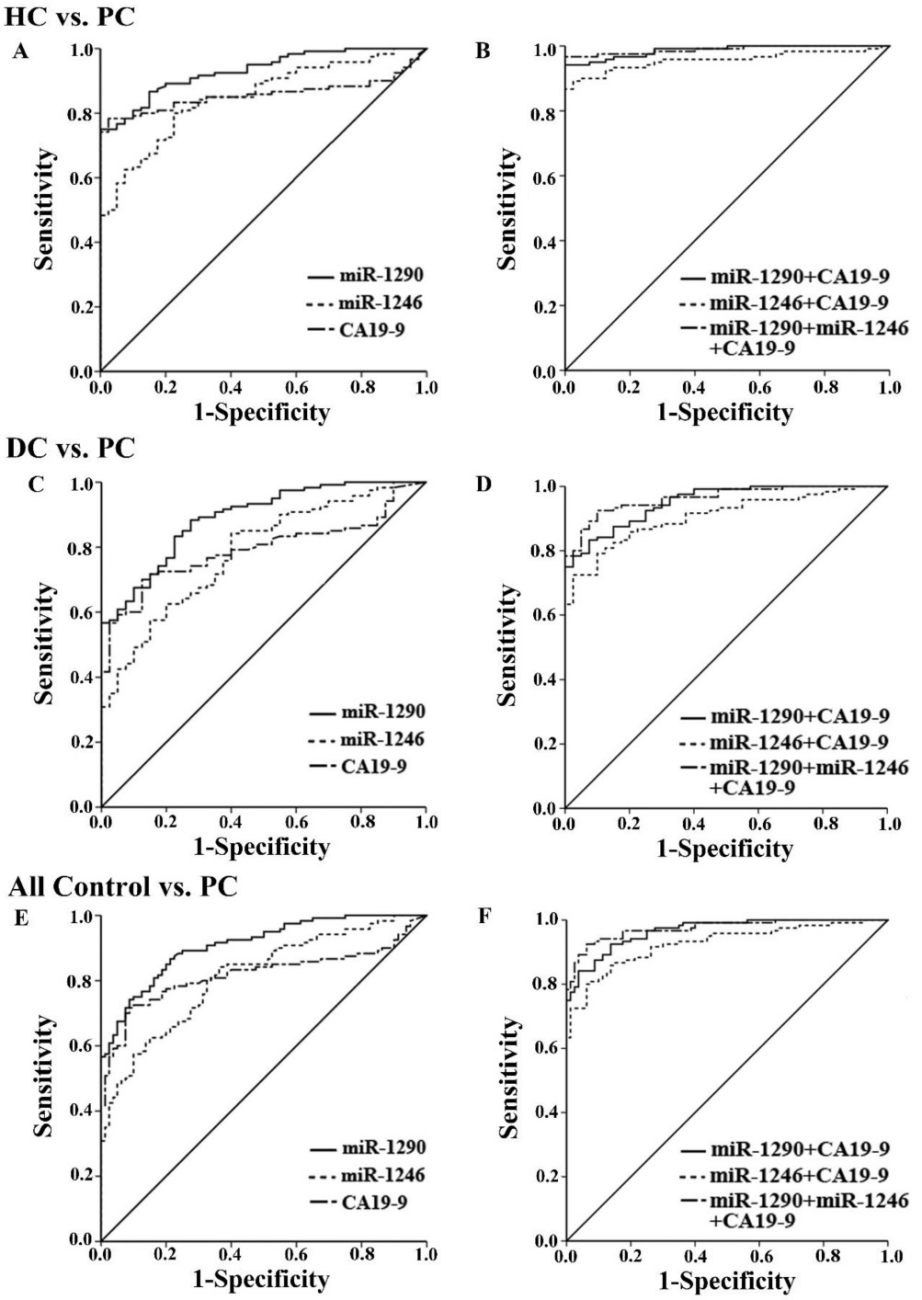
Receiver operating characteristics (ROC) curve analysis of serum miR-1290, miR-1246 and CA19-9 levels. The results of individual and joint diagnostic performance of three tests to distinguish PC patients from HC (A and B), from DC (C and D), from all the controls (E and F).

The ROC curves showed the ability of a single miRNA and the combined tests to differentiate between PC patients from the other controls (Figure 3). Serum miR-1290 had the best discriminating ability with AUC of 0.93 (95% CI: 0.89 to 0.97) against HC, 0.89 (95% CI: 0.84 to 0.94) against DC, and 0.91 (95% CI: 0.87 to 0.95) against all controls. The AUCs for miR‐1246 and CA19-9 were 0.85 (95% CI: 0.79 to 0.91), 0.86 (95% CI: 0.80 to 0.92) against HC, 0.78 (95% CI: 0.71 to 0.86), 0.79 (95% CI: 0.73 to 0.86) against DC and 0.81 (95% CI: 0.75 to 0.87), 0.82 (95% CI: 0.76 to 0.88) against all controls respectively. Besides, the best cutoff value for discrimination was 4764.87 fmol/l for miR-1290, exhibiting a sensitivity of 74.2% and a specificity of 91.2%. For serum miR-1246, the optimal cutoff value was 781.40 fmol/l with a sensitivity of 84.2% and a specificity of 63.7%.

Next, we evaluated the diagnostic utility of two tests by combining miR-1290 or miR-1246 with CA19-9 (Figure 3). When either of miRNAs and CA19-9 were combined, the AUCs for the panel of 2 markers significantly exceeded that of the single CA19-9 assay (Table 2, all P<0.05). We further combined the two miRNAs together with CA19-9 to compare its diagnostic utility for PC with the above 2-marker panel. The AUC for the three markers was 0.99 (95%CI: 0.97 to 1.00) with 96.7% sensitivity and 97.5% specificity against HC, 0.96 (95%CI: 0.93 to 0.99) with 92.5% sensitivity and 90% specificity against DC, as well as 0.97 (95%CI: 0.96 to 0.99) with 92.5% sensitivity and 93.7% specificity against all controls (all P<0.001). Although the AUCs for 3-marker panel were highest among all groups, the diagnostic accuracy of it was not improved statistically when compared to the joint measurement of miR-1290 and CA19-9 (all P>0.05). All the data about ROC curve analysis was shown in Table 2.

In addition, a significant correlation between miR-1290 and CA19-9 was observed (r=0.470, p<0.001), which could also be found between miR-1246 and CA19-9 (r=0.281, p<0.001).

### Both miR-1290 and miR-1246 were independent risk factors for PC

Results of the univariate analysis are summarized in Table 3. The significant variables including age (≥ 60 years old), gender, miR-1290, miR-1246 and CA19-9 were associated with the onset of PC in comparison with DC groups (all P<0.05, Table 3). All the variables were entered into the multivariate logistic regression model to determine the independent significant ones. The results showed that miR-1290 (OR=12.35, P<0.001) and miR-1246 (OR=4.18, P=0.025) remained significant and were independent risk factors for PC (Table 3).

### Bioinformatics analysis of miR-1290 and miR-1246 for PC

Heat‐maps of pathway investigation of KEGG and GO analyses were shown in Supplementary Figure S2. KEGG analysis revealed that both the miRNAs were involved in the cancer‐related signal pathways regulating pluripotency of stem cells. MiR-1290 occupied a more dominant regulated position in tumor-associated pathways, such as choline metabolism and proteoglycans (Supplementary Figure S2A). GO analysis showed intersection of the pathways regarding each individual miRNA. These two miRNAs had close relationship with some processes including responsivity to organelle, ion binding and metabolic process of cellular nitrogen compounds. Moreover, we found that miR-1290 was associated with phosphatidylinositol-mediated pathway, Fc-ε receptor and epidermal growth factor receptor signaling pathways (Supplementary Figure S2B).

## Discussion

PC is one of the most highly lethal diseases with a 5-years survival of approximately 8% worldwide, and it is highlighted by the close parallel between disease incidence and mortality 1-2. To improve the accuracy of prognosis, novel non-invasive biomarkers for detecting patients at high risk of PC are urgently needed.

MiRNAs have been reported to be differentially expressed during pancreatic carcinogenesis and were considered promising noninvasive biomarkers for PC diagnosis in a few studies 16-18. However, considering the inconsistency among these miRNA profiling studies, we carried out an integrated analysis of three GEO datasets and discovered that serum miR-1290 and miR-1246 were significantly upregulated in PC patients. Moreover, previous studies have shown that miR-1290 and miR-1246 both underwent significant changes in PC tissues compared with non-cancer specimens 19-21. Hence, we inferred that these two miRNAs may be novel biomarkers for PC and explored the potential of candidate miRNAs (miR-1290 and miR-1246) for PC diagnosis in this study.

The detection of relative expression levels of miRNAs was frequently used in majority of studies, while absolute quantification data for PC is insufficient so far 22. Here, we accessed serum miR-1290 and miR-1246 levels by absolute quantification to obtain the more reliable results. As expected, both miRNAs increased in PC patients (all P<0.001). Meanwhile, serum concentrations of these two miRNAs decreased obviously after the resection of malignant tumor (all P<0.001). It suggested that serum miR-1290 and miR-1246 were tumor-derived and might be served as potential biomarkers for the diagnosis of PC in Chinese patients.

We also disclosed that serum miR-1290 and miR-1246 shared a positive correlation with TNM stage and tumor size respectively. Results of logistic analyses showed that both of them were determined as independent risk factors for the onset of PC, indicating that both miR-1290 and miR-1246 might play an important role in oncogenesis of PC. Ta N et al. 20 declared that miR-1290 functioned as a tumor oncogene and enhanced PC cell proliferation, invasion, and migration by directly targeting the 3' UTR of IKK1 21. Meantime, a previous study also proved that miR-1290 could promote pancreatic ductal adenocarcinoma malignant progression by silencing the FOXA1/2, which was involved in epithelial-to-mesenchymal transitions 23. In addition, Hasegawa S et al. confirmed that highly expressed miR-1246 facilitated chemoresistance via targeting CCNG2, and could predict worse prognosis in pancreatic cancer patients 24. Discoveries described above indicated that both miR-1290 and miR-1246 involved in the tumorigenesis and progression of PC.

The diagnostic efficiency of circulating miR-1290 and miR-1246 were tested in various cancers, including non-small cell lung cancer, prostate cancer and PC 11, 21, 25-28. Serum miR-1290 had superior ability to identify PC patients than serum miR-1246 and CA19-9, which was in agreement with the findings of Li et al 21. Although elevated serum CA 19-9 may signal pancreatic lesions in the appropriate context of clinical and radiological data, it still could be false negative in Lewis negative phenotype and false positive in other forms of digestive tract cancer and some non-cancerous conditions 29. Thus, tumor-specific miRNAs are more suitable biomarkers, compensating for serum CA19-9, for PC diagnosis 30. Several studies have demonstrated that the combination of miRNAs or protein biomarkers could improve the diagnostic performance than a single CA19-9 did 28, 31-32. We then accessed the diagnostic efficacy of the joint measurement (miRNAs and CA19-9). Surprisingly, when CA19-9 was combined with either miR-1290 or miR-1246, the discriminatory ability was significantly improved than that of single serum CA19-9 in our study (all P <0.05). Besides, the diagnostic accuracy of the combination of 3 markers (miR-1290+miR-1246+CA19-9) was also significantly improved when compared to the 2-marker panel (miR-1246+CA19-9). However, there was no significant difference between the AUCs for the 2-marker panel (miR-1290+CA19-9) and the 3-marker panel (miR-1290+miR-1246+CA19-9). Results of KEGG and GO pathway analysis showed that miR-1290 had a more predominant role in cancer-related pathways than miR-1246, such as proteoglycans and epidermal growth factor receptor signaling pathways. This may explain the better discriminatory ability of serum miR-1290 than miR-1246.

Recent studies have shown that specific exosomal miRNAs (exo-miRNAs) are related to the phenotypes of certain types of cancer 33-35. The value of serum miR-1290 and miR-1246 as tumor biomarkers has been comprehensively investigated by Zou and Madhavan et al. 27, 31. However, there are some disadvantages for exo-miRNA research approaches due to the lack of normalization on the isolation of exosomes and absolute quantification. Furthermore, some miRNAs are quite different distributed inside and outside exosomes. Therefore, we identified the diagnostic performance of absolute serum miR-1290 and miR-1246 levels in PC, which was more reliable in clinical practice. And, the steps are relatively simple and require less sample volume.

Although circulating miR-1290 and miR-1246 appear to be reliable and noninvasive biomarkers for PC diagnosis, there are some limitations in our study. The sample size was relatively small, and expression levels of two miRNAs were not validated in PC tissues samples. In addition, prospective studies with larger sample size are required to explore their prognostic value for PC.

## Conclusion

This study demonstrated that serum miR-1290 and miR-1246 could potentially serve as novel non-invasive biomarkers for PC diagnosis.

## Supplementary Material

Supplementary figures and table.

Supplementary table.

## Figures and Tables

**Table 1:** Correlation between serum miRNAs levels and clinical characteristics.

Characteristics	miR-1290			miR-1246		
	Low(n=60)	High(n=60)	P value	Low(n=60)	High(n=60)	P value
	Cases (%)	Cases (%)		Cases (%)	Cases (%)	
Age (≥60)	41 (68.33)	40 (66.67)	0.845	45 (75.00)	36 (60.00)	0.079
Gender (Male)	29 (48.33)	41 (68.33)	0.026	30 (50.00)	40 (66.67)	0.064
Tumor location			0.882			0.078
Head	38 (63.33)	40 (66.67)		36 (60.00)	42 (70.00)	
Body or tail	17 (28.33)	19 (31.67)		23 (38.33)	13 (21.67)	
NA	5 (8.33)	1 (1.67)		1 (1.67)	5 (8.33)	
Tumor size			0.345			0.026
≥4.0cm	30 (50.00)	24 (40.00)		21 (35.00)	33 (55.00)	
< 4.0cm	29 (48.33)	33 (55.00)		37 (61.67)	25 (41.67)	
NA	1 (1.67)	3 (5.00)		2 (3.33)	2 (3.33)	
Differential			0.984			0.243
Poor	13 (21.67)	15 (25.00)		11(18.33)	17 (28.33)	
Well	21 (35.00)	24 (40.00)		24 (40.00)	21 (35.00)	
NA	26 (43.33)	21 (35.00)		25 (41.67)	22 (36.67)	
Tumor stage			0.043			0.624
I-II	14 (23.33)	6 (10.00)		11(18.33)	9 (15.00)	
III-IV	46 (76.67)	54 (90.00)		49 (81.67)	51 (85.00)	
Lymph nodemetastasis			0.877			0.772
Yes	30 (50.00)	32 (53.33)		33 (55.00)	29 (48.33)	
No	14 (23.33)	16 (26.67)		15 (25.00)	15 (25.00)	
NA	16 (26.67)	12 (20.00)		12 (20.00)	16 (26.67)	
Diabetesmellitus			0.852			0.341
Yes	11(18.33)	11(18.33)		9 (15.00)	13 (21.67)	
No	43 (71.67)	47 (78.33)		47 (78.33)	43 (71.67)	
NA	6 (10.00)	2 (3.33)		4 (6.67)	4 (6.67)	
Smoking			0.475			0.365
Smoker	9 (15.00)	13 (21.67)		9 (15.00)	13 (21.67)	
Nonsmoker	43 (71.67)	44 (73.33)		45 (75.00)	42 (70.00)	
NA	8 (13.33)	3 (5.00)		6 (10.00)	5 (8.33)	
Drinking			0.506			0.057
Drinker	7 (11.67)	10 (16.67)		5 (8.33)	12 (20.00)	
Nondrinker	43 (71.67)	43 (71.67)		47 (78.33)	39 (65.0)	
NA	10 (16.67)	7 (11.67)		8 (13.33)	9 (15.00)	
CA19-9 (Elevated)	38 (63.33)	37 (61.67)	0.850	39 (65.0)	36 (60.00)	0.572
Glu (Elevated)	27 (45.00)	33 (55.00)	0.273	25 (41.67)	35 (58.33)	0.068
TG (Elevated)	13 (21.67)	7 (11.67)	0.142	8 (13.33)	12 (20.00)	0.327

Low: miRNA low expression (≤ median); High: miRNA high expression (> median); Elevated: laboratory test results exceed the normal reference range (CA19-9: ≥39U/ml; Glu: ≥6.1mmol/l; TG ≥6.1mmol/l); NA: not available.

**Table 2:** Diagnostic performance of serum miRNAs and combined tests.

	AUC	95%	CI	Sensi.(%)	Speci.(%)	Z test	P value
**PC vs. HC**							
miR-1290	0.93*	0.89	0.97	75.0	97.5	-	-
miR-1246	0.85*	0.79	0.91	62.5	92.5	-	-
CA19-9	0.86*	0.80	0.92	78.3	97.5	-	-
miR-1290+CA19-9	0.99*	0.97	1.00	94.2	97.5	4.46^a^	<0.001^a^
miR-1246+CA19-9	0.96*	0.93	0.99	89.2	97.5	3.10^b^	0.002^b^
Two miRNAs+CA19-9	0.99*	0.98	1.00	96.7	97.5	0.33^c^	0.745^c^
						1.93^d^	0.053^d^
**PC vs. DC**							
miR-1290	0.89*	0.84	0.94	88.3	72.5	-	-
miR-1246	0.78*	0.71	0.86	62.5	80.0	-	-
CA19-9	0.79*	0.73	0.86	70.0	87.5	-	-
miR-1290+CA19-9	0.95*	0.92	0.98	83.3	92.5	4.20^a^	<0.001^a^
miR-1246+CA19-9	0.91*	0.86	0.95	72.5	97.5	2.87^b^	0.004^b^
Two miRNAs+CA19-9	0.96*	0.93	0.99	92.5	90.0	0.18^c^	0.855^c^
						2.16^d^	0.031^d^
**PC vs. All Control**							
miR-1290	0.91*	0.87	0.95	74.2	91.2	-	-
miR-1246	0.81*	0.75	0.87	84.2	63.7	-	-
CA19-9	0.82*	0.76	0.88	71.7	91.2	-	-
miR-1290+CA19-9	0.96*	0.94	0.99	84.2	96.2	4.30^a^	<0.001^a^
miR-1246+CA19-9	0.93*	0.89	0.96	85.8	86.2	3.09^b^	0.002^b^
Two miRNAs+CA19-9	0.97*	0.96	0.99	92.5	93.7	1.04^c^	0.298^c^
						2.39^d^	0.017^d^

PC: pancreatic cancer; DC: benign pancreatic disease control; HC: healthy control; Sensi.: sensitivity; Speci.: specificity; Two miRNAs+CA19-9: miR-1290+miR-1246+CA19-9. *: The P value of ROC analysis was <0.001; a: the comparison of AUCs between “CA19-9” and “miR-1290+CA19-9”; b: the comparison of AUCs between “CA19-9” and “miR-1246+CA19-9”; c: the comparison of AUCs between “miR-1290+CA19-9” and “Two miRNAs+CA19-9”; d: the comparison of AUCs between “miR-1246+CA19-9” and “Two miRNAs+CA19-9”.

**Table 3:** Univariate and multivariate analysis of factors associated with PC.

	Univariate analysis			Multivariate analysis		
Factors	OR	95%CI	P value	OR	95%CI	P value
Age (≥60)	2.538	1.22-5.27	**0.012**	4.42	1.41-13.83	**0.011**
Gender (male)	2.10	1.01-4.36	**0.046**	0.95	0.31-2.96	0.936
miR-1290(high)	1.62	1.35-1.95	**<0.001**	12.35	3.73-40.90	**<0.001**
miR-1246 (high)	2.30	1.51-3.51	**<0.001**	4.18	1.20-14.57	**0.025**
CA19-9 (high)	9.44	3.68-24.26	**<0.001**	18.48	4.69-72.77	**<0.001**
DM	1.57	0.55-4.47	0.397	0.36	0.09-1.48	0.157
Smoking	1.57	0.55-4.47	0.397	1.22	0.21-7.21	0.828
Drinking	1.49	0.47-4.71	0.501	2.18	0.25-18.76	0.478

OR: odds ratio; DM: diabetes mellitus; high: the expression of miRNAs exceeded the optimal cutoff value (miR-1290: 4991.65 fmol/l; miR-1246: 779.38 fmol/l) and the CA19-9 exceeded 39 U/ml.
