# Supplementary material for: Serum miR-1290 and miR-1246 as Potential Diagnostic Biomarkers of Human Pancreatic Cancer
Source: J Cancer. 2020 Jan 1;11(6):1325–33. doi: 10.7150/jca.38048 (PMC6995378; doi:10.7150/jca.38048)
Supplement: Supplementary file 1 — Supplementary figures and table. [file jcav11p1325s1.pdf]

A

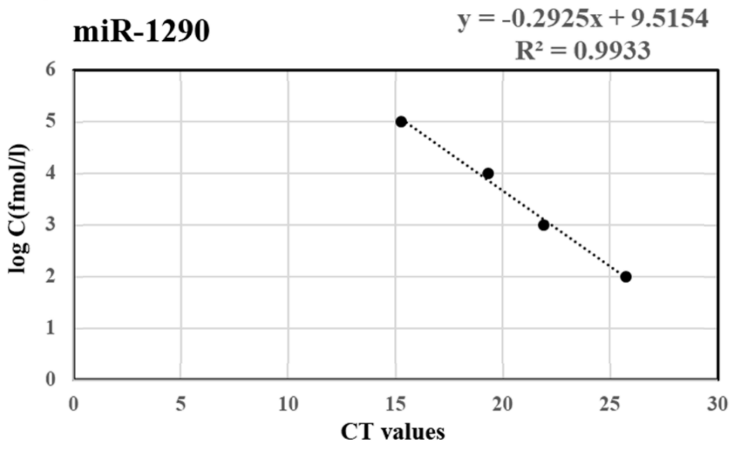

B

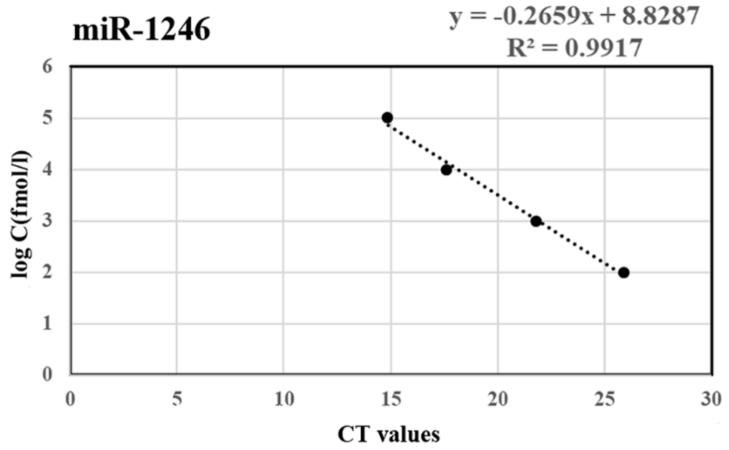

Supplementary Figure S1. Standard curves of miR-1290 and miR-1246 using synthetic mature miRNAs. For each assay, ten-fold serial dilution of synthetic miRNA from 10 fmol/L to 10^7 pmol/L was used to generate the standard curves. The resulting Cq values were plotted versus the $\log_{10}$ of the amount of synthetic miRNAs. Each point represents the mean of three independent experiments.

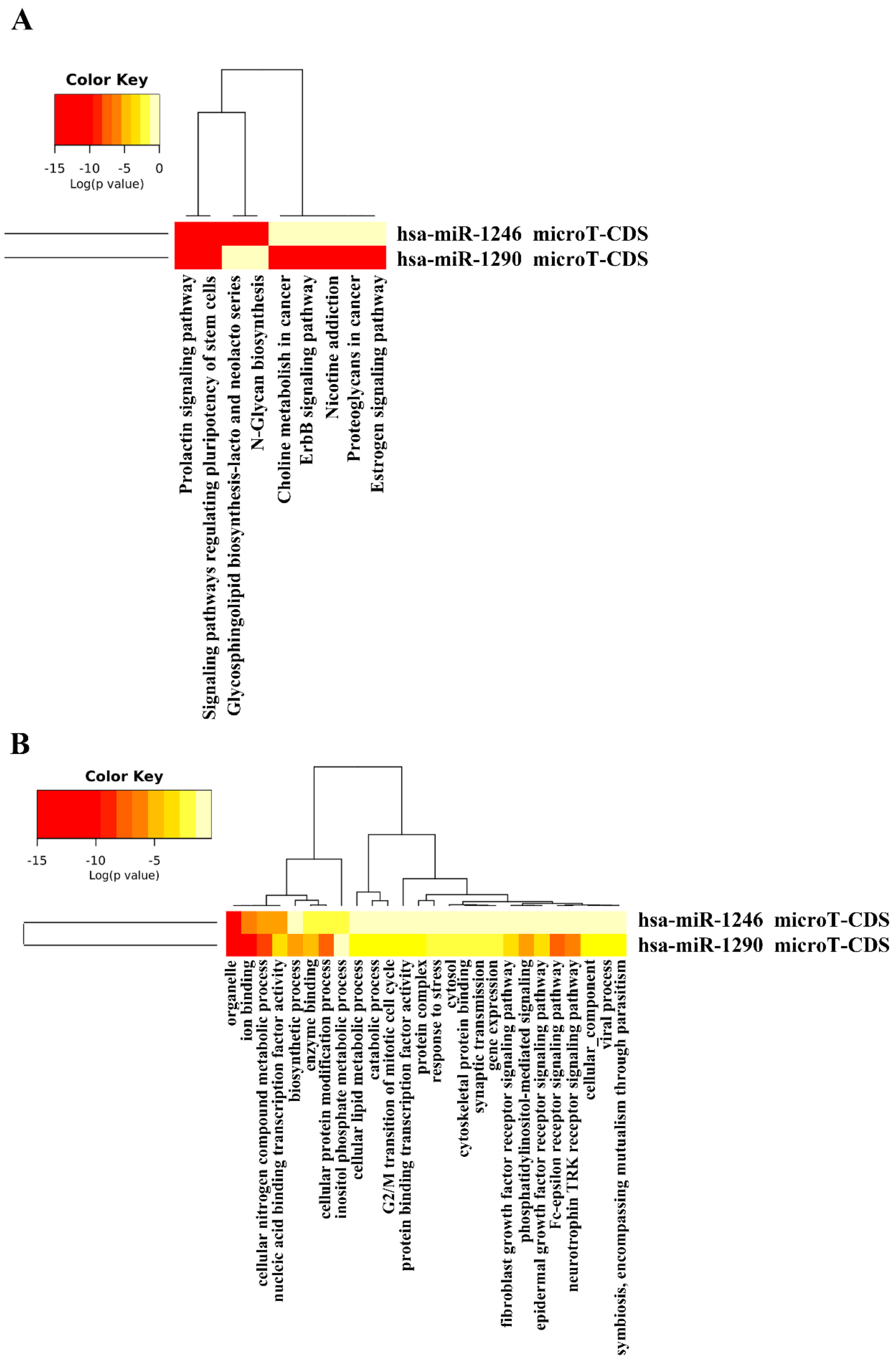

Supplementary Figure S2. Heat-maps of pathway investigation using KEGG (A) and GO (B) analyses of miR-1290 and miR-1246.

KEGG: Kyoto Encyclopedia of Genes and Genomes; GO: Gene Ontology.

Supplementary Table S1

GSE113486 upregulated miRNAs

ID	adj.P.Val	P.Value	t	B	logFC	miRNA_ID_LIST
MIMAT0015072	5.82E-17	1.52E-18	10.2	31.5	4.882	hsa-miR-320e
MIMAT0000683	1.97E-15	5.99E-17	9.54	27.9	4.591	hsa-miR-302a-5p
MIMAT0003254	5.49E-14	2.27E-15	8.92	24.3	4.513	hsa-miR-548b-3p
MIMAT0023698	4.72E-11	4.03E-12	7.59	16.9	4.485	hsa-miR-6073
MIMAT0005898	2.11E-09	3.06E-10	6.78	12.7	4.483	hsa-miR-1246
MIMAT0004597	2.48E-14	9.39E-16	9.07	25.2	4.48	hsa-miR-140-3p
MIMAT0000440	8.97E-12	6.68E-13	7.92	18.7	4.363	hsa-miR-191-5p
MIMAT0004697	2.96E-16	8.42E-18	9.88	29.8	4.304	hsa-miR-151a-5p
MIMAT0019025	3.33E-12	2.19E-13	8.12	19.8	4.295	hsa-miR-4490
MIMAT0003252	1.41E-11	1.10E-12	7.83	18.2	4.294	hsa-miR-586
MIMAT0005880	8.13E-12	5.96E-13	7.94	18.8	4.187	hsa-miR-1290
MIMAT0004810	6.34E-11	5.78E-12	7.52	16.6	4.178	hsa-miR-629-5p
MIMAT0004490	2.77E-11	2.27E-12	7.7	17.5	4.178	hsa-miR-19a-5p
MIMAT0000083	1.65E-12	1.02E-13	8.25	20.6	4.148	hsa-miR-26b-5p
MIMAT0019232	1.13E-11	8.62E-13	7.87	18.5	4.107	hsa-miR-4423-5p
MIMAT0022497	2.58E-12	1.64E-13	8.17	20.1	4.086	hsa-miR-5692b
MIMAT0000064	4.18E-10	4.91E-11	7.13	14.5	4.081	hsa-let-7c-5p
MIMAT0015018	3.12E-14	1.20E-15	9.03	24.9	4.062	hsa-miR-3146
MIMAT0019223	4.13E-12	2.78E-13	8.07	19.6	4.045	hsa-miR-3688-5p
MIMAT0010214	7.35E-12	5.33E-13	7.96	18.9	4.033	hsa-miR-151b
MIMAT0000062	3.00E-10	3.35E-11	7.2	14.9	3.999	hsa-let-7a-5p
MIMAT0000099	6.13E-11	5.54E-12	7.53	16.6	3.987	hsa-miR-101-3p
MIMAT0002868	1.29E-14	4.59E-16	9.2	25.9	3.956	hsa-miR-522-3p
MIMAT0001532	8.88E-12	6.57E-13	7.92	18.7	3.919	hsa-miR-448
MIMAT0004505	9.36E-12	7.00E-13	7.91	18.7	3.912	hsa-miR-32-3p
MIMAT0000097	7.07E-11	6.59E-12	7.5	16.5	3.907	hsa-miR-99a-5p
MIMAT0019935	2.81E-10	3.12E-11	7.21	14.9	3.896	hsa-miR-4777-3p
MIMAT0003255	1.98E-12	1.23E-13	8.22	20.4	3.866	hsa-miR-588
MIMAT0000069	1.10E-09	1.47E-10	6.92	13.4	3.861	hsa-miR-16-5p
MIMAT0000065	3.05E-09	4.63E-10	6.7	12.3	3.86	hsa-let-7d-5p
MIMAT0000252	1.65E-12	1.01E-13	8.25	20.6	3.854	hsa-miR-7-5p
MIMAT0019199	1.89E-11	1.52E-12	7.77	17.9	3.845	hsa-miR-3121-5p
MIMAT0004681	5.50E-11	4.83E-12	7.56	16.8	3.843	hsa-miR-26a-2-3p
MIMAT0000461	6.29E-10	7.89E-11	7.04	14	3.829	hsa-miR-195-5p
MIMAT0000254	4.95E-11	4.27E-12	7.58	16.9	3.829	hsa-miR-10b-5p
MIMAT0019763	6.36E-14	2.78E-15	8.88	24.1	3.826	hsa-miR-4679
MIMAT0019959	1.48E-09	2.03E-10	6.86	13.1	3.803	hsa-miR-4789-5p
MIMAT0000436	7.50E-11	7.02E-12	7.49	16.4	3.797	hsa-miR-144-3p
MIMAT0000098	1.34E-10	1.35E-11	7.37	15.8	3.795	hsa-miR-100-5p
MIMAT0002870	3.97E-14	1.55E-15	8.99	24.7	3.793	hsa-miR-499a-5p
MIMAT0019785	4.64E-14	1.86E-15	8.95	24.5	3.767	hsa-miR-4693-3p
MIMAT0000082	1.27E-08	2.24E-09	6.39	10.7	3.762	hsa-miR-26a-5p
MIMAT0026621	5.77E-14	2.47E-15	8.9	24.2	3.753	hsa-miR-605-3p
MIMAT0019875	1.39E-10	1.43E-11	7.36	15.7	3.752	hsa-miR-4744
MIMAT0003238	3.53E-10	4.00E-11	7.17	14.7	3.745	hsa-miR-573
MIMAT0004768	5.77E-14	2.46E-15	8.91	24.2	3.694	hsa-miR-497-3p
MIMAT0002864	1.29E-09	1.76E-10	6.89	13.2	3.687	hsa-miR-518d-3p
MIMAT0000274	1.87E-10	2.01E-11	7.29	15.4	3.681	hsa-miR-217
MIMAT0017984	4.29E-17	1.07E-18	10.2	31.9	3.676	hsa-miR-3607-5p
MIMAT0002888	1.20E-13	5.39E-15	8.77	23.5	3.67	hsa-miR-532-5p
MIMAT0000096	6.30E-10	7.94E-11	7.04	14	3.663	hsa-miR-98-5p
MIMAT0026478	1.25E-11	9.62E-13	7.85	18.3	3.654	hsa-miR-133a-5p
MIMAT0003272	1.37E-11	1.06E-12	7.83	18.2	3.646	hsa-miR-604
MIMAT0026623	5.77E-14	2.45E-15	8.91	24.2	3.639	hsa-miR-627-3p
MIMAT0003258	5.80E-11	5.14E-12	7.55	16.7	3.624	hsa-miR-590-5p

MIMAT0016844	5.63E-10	6.87E-11	7.06	14.1	3.623	hsa-miR-4295
MIMAT0000079	3.80E-09	5.97E-10	6.65	12	3.616	hsa-miR-24-1-5p
MIMAT0019805	2.03E-15	6.25E-17	9.54	27.9	3.603	hsa-miR-4705
MIMAT0022301	1.44E-16	3.94E-18	10	30.6	3.598	hsa-miR-5591-5p
MIMAT0019731	2.24E-13	1.09E-14	8.65	22.8	3.595	hsa-miR-4662a-5p
MIMAT0000688	2.47E-11	2.01E-12	7.72	17.6	3.592	hsa-miR-301a-3p
MIMAT0000419	1.34E-10	1.35E-11	7.37	15.8	3.589	hsa-miR-27b-3p
MIMAT0006764	4.16E-10	4.87E-11	7.13	14.5	3.578	hsa-miR-320d
MIMAT0000071	2.36E-11	1.91E-12	7.73	17.7	3.577	hsa-miR-17-3p
MIMAT0002849	1.69E-09	2.37E-10	6.83	12.9	3.574	hsa-miR-524-5p
MIMAT0000425	7.69E-10	9.86E-11	7	13.8	3.53	hsa-miR-130a-3p
MIMAT0003253	1.09E-10	1.07E-11	7.41	16	3.521	hsa-miR-587
MIMAT0014983	7.55E-13	4.29E-14	8.41	21.4	3.506	hsa-miR-3121-3p
MIMAT0004793	4.44E-10	5.24E-11	7.12	14.4	3.505	hsa-miR-556-3p
MIMAT0019929	2.62E-12	1.68E-13	8.16	20.1	3.503	hsa-miR-4774-5p
MIMAT0004749	1.88E-10	2.03E-11	7.29	15.3	3.503	hsa-miR-424-3p
MIMAT0021037	5.07E-10	6.12E-11	7.09	14.3	3.499	hsa-miR-548ap-5p
MIMAT0017985	5.50E-15	1.78E-16	9.36	26.8	3.487	hsa-miR-3607-3p
MIMAT0022285	1.25E-13	5.65E-15	8.76	23.4	3.484	hsa-miR-5585-5p
MIMAT0022706	1.61E-13	7.60E-15	8.71	23.1	3.473	hsa-miR-561-5p
MIMAT0019741	3.66E-09	5.72E-10	6.66	12.1	3.472	hsa-miR-4666a-5p
MIMAT0027627	8.98E-10	1.17E-10	6.96	13.6	3.472	hsa-miR-6863
MIMAT0003279	1.47E-10	1.54E-11	7.34	15.6	3.468	hsa-miR-611
MIMAT0004698	6.99E-13	3.91E-14	8.42	21.5	3.464	hsa-miR-135b-3p
MIMAT0000717	4.76E-09	7.65E-10	6.6	11.8	3.454	hsa-miR-302c-3p
MIMAT0004913	1.19E-12	7.03E-14	8.32	20.9	3.449	hsa-miR-891b
MIMAT0016902	1.33E-12	8.01E-14	8.29	20.8	3.446	hsa-miR-4272
MIMAT0019078	6.03E-13	3.29E-14	8.45	21.7	3.439	hsa-miR-4536-5p
MIMAT0000066	7.67E-09	1.28E-09	6.5	11.3	3.418	hsa-let-7e-5p
MIMAT0003287	1.37E-11	1.07E-12	7.83	18.2	3.415	hsa-miR-618
MIMAT0002850	1.86E-08	3.39E-09	6.31	10.3	3.401	hsa-miR-524-3p
MIMAT0004688	1.78E-10	1.90E-11	7.3	15.4	3.395	hsa-miR-374a-3p
MIMAT0002883	3.34E-11	2.80E-12	7.66	17.3	3.395	hsa-miR-514a-3p
MIMAT0022500	9.07E-11	8.66E-12	7.45	16.2	3.395	hsa-miR-5706
MIMAT0016885	5.01E-11	4.34E-12	7.58	16.9	3.391	hsa-miR-4255
MIMAT0004972	4.08E-10	4.76E-11	7.13	14.5	3.381	hsa-miR-922
MIMAT0027632	1.33E-12	7.97E-14	8.3	20.8	3.379	hsa-miR-6866-5p
MIMAT0016898	3.00E-09	4.48E-10	6.71	12.3	3.374	hsa-miR-4263
MIMAT0019024	1.39E-10	1.42E-11	7.36	15.7	3.373	hsa-miR-548al
MIMAT0030427	3.44E-11	2.90E-12	7.65	17.3	3.357	hsa-miR-7852-3p
MIMAT0002867	2.06E-09	2.98E-10	6.79	12.7	3.353	hsa-miR-520h
MIMAT0003220	6.91E-13	3.83E-14	8.43	21.5	3.353	hsa-miR-556-5p
MIMAT0019901	1.32E-07	2.79E-08	5.88	8.27	3.336	hsa-miR-4757-5p
MIMAT0019937	8.25E-12	6.08E-13	7.93	18.8	3.335	hsa-miR-4778-3p
MIMAT0000067	1.17E-08	2.04E-09	6.41	10.8	3.335	hsa-let-7f-5p
MIMAT0000435	6.39E-09	1.05E-09	6.54	11.5	3.335	hsa-miR-143-3p
MIMAT0003288	1.47E-10	1.54E-11	7.34	15.6	3.326	hsa-miR-619-3p
MIMAT0025466	5.99E-10	7.43E-11	7.05	14.1	3.322	hsa-miR-6505-5p
MIMAT0022484	1.69E-09	2.37E-10	6.83	12.9	3.317	hsa-miR-5692a
MIMAT0004699	7.04E-11	6.53E-12	7.5	16.5	3.315	hsa-miR-148b-5p
MIMAT0020959	7.61E-16	2.20E-17	9.72	28.9	3.315	hsa-miR-4536-3p
MIMAT0026606	1.01E-09	1.33E-10	6.94	13.5	3.308	hsa-miR-511-3p
MIMAT0026722	3.44E-15	1.09E-16	9.44	27.3	3.289	hsa-miR-208b-5p
MIMAT0002810	3.71E-08	7.15E-09	6.16	9.6	3.278	hsa-miR-202-5p
MIMAT0031175	3.05E-12	1.97E-13	8.13	19.9	3.275	hsa-miR-548ba
MIMAT0015040	2.39E-10	2.62E-11	7.25	15.1	3.271	hsa-miR-3166
MIMAT0000449	7.34E-08	1.50E-08	6.01	8.88	3.268	hsa-miR-146a-5p
MIMAT0003319	2.24E-09	3.26E-10	6.77	12.6	3.264	hsa-miR-649

MIMAT0019727	6.86E-09	1.14E-09	6.53	11.4	3.253	hsa-miR-4659a-3p
MIMAT0019745	1.98E-13	9.51E-15	8.67	22.9	3.25	hsa-miR-4668-5p
MIMAT0019236	8.31E-09	1.39E-09	6.49	11.2	3.248	hsa-miR-4529-5p
MIMAT0018083	3.05E-13	1.56E-14	8.58	22.4	3.244	hsa-miR-3662
MIMAT0022965	7.35E-12	5.32E-13	7.96	18.9	3.24	hsa-miR-3606-3p
MIMAT0003269	3.87E-10	4.47E-11	7.15	14.6	3.234	hsa-miR-601
MIMAT0000733	4.41E-09	7.02E-10	6.62	11.9	3.232	hsa-miR-379-5p
MIMAT0000101	1.32E-07	2.81E-08	5.88	8.27	3.228	hsa-miR-103a-3p
MIMAT0019924	1.40E-10	1.45E-11	7.36	15.7	3.225	hsa-miR-4770
MIMAT0002866	5.93E-09	9.72E-10	6.56	11.6	3.223	hsa-miR-517c-3p
MIMAT0015004	1.82E-10	1.94E-11	7.3	15.4	3.221	hsa-miR-544b
MIMAT0003285	1.26E-09	1.71E-10	6.89	13.3	3.219	hsa-miR-548c-3p
MIMAT0000718	9.56E-09	1.62E-09	6.46	11.1	3.217	hsa-miR-302d-3p
MIMAT0004596	2.75E-09	4.06E-10	6.73	12.4	3.216	hsa-miR-138-2-3p
MIMAT0003331	1.26E-08	2.20E-09	6.4	10.8	3.214	hsa-miR-655-3p
MIMAT0019832	4.98E-14	2.02E-15	8.94	24.4	3.212	hsa-miR-4719
MIMAT0019730	2.33E-10	2.54E-11	7.25	15.1	3.212	hsa-miR-4661-3p
MIMAT0019816	9.43E-14	4.19E-15	8.81	23.7	3.199	hsa-miR-4711-5p
MIMAT0031893	4.24E-08	8.27E-09	6.13	9.46	3.198	hsa-miR-181b-2-3p
MIMAT0022289	1.10E-09	1.48E-10	6.92	13.4	3.193	hsa-miR-5587-5p
MIMAT0001631	1.45E-06	3.91E-07	5.33	5.71	3.19	hsa-miR-451a
MIMAT0016872	6.08E-10	7.59E-11	7.05	14.1	3.188	hsa-miR-4317
MIMAT0010497	3.26E-11	2.72E-12	7.66	17.3	3.186	hsa-miR-759
MIMAT0019014	1.25E-08	2.19E-09	6.4	10.8	3.184	hsa-miR-4480
MIMAT0004601	6.68E-12	4.77E-13	7.98	19	3.182	hsa-miR-145-3p
MIMAT0019761	1.16E-10	1.15E-11	7.4	15.9	3.181	hsa-miR-4677-3p
MIMAT0018111	9.50E-13	5.52E-14	8.36	21.2	3.174	hsa-miR-3683
MIMAT0017997	3.28E-09	5.04E-10	6.68	12.2	3.174	hsa-miR-3617-5p
MIMAT0022287	8.15E-11	7.72E-12	7.47	16.3	3.172	hsa-miR-5586-5p
MIMAT0018990	2.39E-09	3.50E-10	6.75	12.6	3.17	hsa-miR-548aj-3p
MIMAT0017983	8.98E-14	3.96E-15	8.82	23.8	3.169	hsa-miR-3606-5p
MIMAT0018078	4.74E-12	3.27E-13	8.04	19.4	3.167	hsa-miR-3658
MIMAT0000241	2.51E-12	1.58E-13	8.17	20.1	3.164	hsa-miR-208a-3p
MIMAT0019230	6.49E-08	1.31E-08	6.04	9.01	3.158	hsa-miR-3942-3p
MIMAT0027564	6.02E-10	7.49E-11	7.05	14.1	3.156	hsa-miR-6832-5p
MIMAT0013517	1.72E-09	2.42E-10	6.83	12.9	3.154	hsa-miR-2682-5p
MIMAT0003328	1.49E-10	1.57E-11	7.34	15.6	3.141	hsa-miR-653-5p
MIMAT0000279	9.69E-08	2.03E-08	5.95	8.59	3.141	hsa-miR-222-3p
MIMAT0035704	1.05E-10	1.02E-11	7.42	16	3.141	hsa-miR-548bb-3p
MIMAT0004700	8.86E-10	1.15E-10	6.97	13.6	3.138	hsa-miR-331-5p
MIMAT0007399	7.66E-08	1.57E-08	6	8.83	3.133	hsa-miR-1537-3p
MIMAT0019914	6.99E-13	3.92E-14	8.42	21.5	3.133	hsa-miR-4764-5p
MIMAT0003260	1.58E-08	2.82E-09	6.35	10.5	3.127	hsa-miR-592
MIMAT0000084	5.25E-08	1.03E-08	6.09	9.24	3.107	hsa-miR-27a-3p
MIMAT0002871	3.73E-08	7.21E-09	6.16	9.59	3.09	hsa-miR-500a-3p
MIMAT0000684	1.60E-08	2.86E-09	6.35	10.5	3.09	hsa-miR-302a-3p
MIMAT0031006	2.82E-09	4.19E-10	6.72	12.4	3.088	hsa-miR-8079
MIMAT0018182	2.20E-14	8.16E-16	9.1	25.3	3.088	hsa-miR-3908
MIMAT0000693	1.27E-07	2.68E-08	5.89	8.31	3.087	hsa-miR-30e-3p
MIMAT0005928	1.19E-09	1.61E-10	6.9	13.3	3.085	hsa-miR-548h-5p
MIMAT0026638	3.41E-09	5.31E-10	6.67	12.1	3.081	hsa-miR-1468-3p
MIMAT0018112	6.95E-12	4.99E-13	7.97	19	3.08	hsa-miR-3684
MIMAT0005879	3.22E-08	6.12E-09	6.19	9.75	3.078	hsa-miR-1289
MIMAT0019790	7.55E-13	4.30E-14	8.4	21.4	3.076	hsa-miR-4696
MIMAT0005932	1.09E-12	6.40E-14	8.33	21	3.072	hsa-miR-302f
MIMAT0004692	4.44E-10	5.27E-11	7.11	14.4	3.068	hsa-miR-340-5p
MIMAT0026611	1.49E-09	2.05E-10	6.86	13.1	3.063	hsa-miR-520g-5p
MIMAT0018979	1.39E-10	1.43E-11	7.36	15.7	3.062	hsa-miR-4457

MIMAT0026624	2.02E-13	9.77E-15	8.67	22.9	3.058	hsa-miR-651-3p
MIMAT0000278	3.28E-09	5.05E-10	6.68	12.2	3.057	hsa-miR-221-3p
MIMAT0005948	1.63E-11	1.29E-12	7.8	18.1	3.05	hsa-miR-664a-5p
MIMAT0001413	4.82E-07	1.17E-07	5.59	6.88	3.048	hsa-miR-20b-5p
MIMAT0018195	1.51E-07	3.25E-08	5.85	8.12	3.041	hsa-miR-3920
MIMAT0018446, M	1.57E-10	1.67E-11	7.33	15.5	3.038	hsa-miR-548z, hsa-miR-548h-3p
MIMAT0000420	9.60E-10	1.25E-10	6.95	13.6	3.036	hsa-miR-30b-5p
MIMAT0004685	9.22E-09	1.55E-09	6.47	11.1	3.033	hsa-miR-302d-5p
MIMAT0000089	6.21E-08	1.24E-08	6.05	9.06	3.032	hsa-miR-31-5p
MIMAT0003327	6.52E-08	1.32E-08	6.04	9.01	3.031	hsa-miR-449b-5p
MIMAT0004784	3.22E-08	6.13E-09	6.19	9.75	3.028	hsa-miR-455-3p
MIMAT0000281	1.38E-07	2.95E-08	5.87	8.22	3.027	hsa-miR-224-5p
MIMAT0004594	1.88E-09	2.67E-10	6.81	12.8	3.021	hsa-miR-132-5p
MIMAT0017988	1.33E-12	8.02E-14	8.29	20.8	3.02	hsa-miR-3611
MIMAT0002848	4.66E-07	1.12E-07	5.59	6.92	3.018	hsa-miR-518c-3p
MIMAT0000434	2.14E-07	4.82E-08	5.77	7.74	3.018	hsa-miR-142-3p
MIMAT0019080	1.58E-09	2.20E-10	6.84	13	3.018	hsa-miR-4537
MIMAT0026765	4.45E-12	3.04E-13	8.06	19.5	3.016	hsa-miR-1537-5p
MIMAT0004683	1.79E-07	3.95E-08	5.81	7.94	3.015	hsa-miR-362-3p
MIMAT0005793	6.68E-17	1.77E-18	10.1	31.4	3.014	hsa-miR-320c
MIMAT0019890	1.84E-08	3.34E-09	6.31	10.3	3.013	hsa-miR-4753-5p
MIMAT0005909	1.52E-15	4.57E-17	9.59	28.2	3.012	hsa-miR-1258
MIMAT0021131	1.39E-10	1.43E-11	7.36	15.7	3.011	hsa-miR-5197-3p
MIMAT0018198	1.64E-12	9.98E-14	8.26	20.6	3.011	hsa-miR-3923
MIMAT0005913	2.93E-11	2.42E-12	7.68	17.4	3.009	hsa-miR-1261
MIMAT0001536	1.91E-07	4.23E-08	5.8	7.87	3.005	hsa-miR-429
MIMAT0003317	1.94E-07	4.31E-08	5.79	7.85	3.005	hsa-miR-647
MIMAT0002882	4.68E-09	7.48E-10	6.61	11.8	3.005	hsa-miR-510-5p
MIMAT0003251	1.75E-07	3.84E-08	5.82	7.96	3.005	hsa-miR-548a-3p
MIMAT0027579	1.56E-09	2.16E-10	6.85	13	3.004	hsa-miR-6838-3p
MIMAT0016925	3.19E-10	3.60E-11	7.19	14.8	3.002	hsa-miR-500b-5p
MIMAT0004492	8.86E-08	1.84E-08	5.97	8.68	3.001	hsa-miR-19b-2-5p
MIMAT0015039	5.81E-10	7.16E-11	7.06	14.1	3.001	hsa-miR-3165
MIMAT0019782	3.67E-10	4.18E-11	7.16	14.6	3	hsa-miR-4691-3p
MIMAT0018989, M	4.07E-14	1.60E-15	8.98	24.7	2.997	hsa-miR-548ai, hsa-miR-570-5p
MIMAT0005893	7.67E-08	1.58E-08	6	8.83	2.993	hsa-miR-1305
MIMAT0019873	5.57E-08	1.10E-08	6.08	9.18	2.987	hsa-miR-4742-3p
MIMAT0005931	5.20E-12	3.61E-13	8.03	19.3	2.985	hsa-miR-302e
MIMAT0022861	2.76E-13	1.37E-14	8.61	22.5	2.98	hsa-miR-376c-5p
MIMAT0014998	3.86E-10	4.43E-11	7.15	14.6	2.976	hsa-miR-3133
MIMAT0002869	1.17E-08	2.04E-09	6.41	10.8	2.976	hsa-miR-519a-3p
MIMAT0022259	5.76E-23	6.06E-25	12.6	46.1	2.972	hsa-miR-5100
MIMAT0000275	1.81E-07	3.99E-08	5.81	7.93	2.964	hsa-miR-218-5p
MIMAT0000454	9.04E-10	1.18E-10	6.96	13.6	2.96	hsa-miR-184
MIMAT0003333	6.51E-11	5.97E-12	7.52	16.6	2.953	hsa-miR-549a
MIMAT0019203	2.48E-12	1.56E-13	8.18	20.1	2.951	hsa-miR-3136-3p
MIMAT0002171	7.11E-07	1.81E-07	5.49	6.46	2.951	hsa-miR-410-3p
MIMAT0019748	3.21E-08	6.09E-09	6.19	9.76	2.95	hsa-miR-219b-3p
MIMAT0000682	1.69E-07	3.69E-08	5.83	8	2.948	hsa-miR-200a-3p
MIMAT0015060	2.20E-09	3.21E-10	6.77	12.6	2.947	hsa-miR-548w
MIMAT0019751	3.01E-09	4.52E-10	6.71	12.3	2.945	hsa-miR-4670-3p
MIMAT0019938	1.79E-11	1.42E-12	7.78	18	2.945	hsa-miR-4779
MIMAT0019005	2.67E-13	1.31E-14	8.61	22.6	2.944	hsa-miR-4477b
MIMAT0004916	2.51E-10	2.77E-11	7.23	15	2.942	hsa-miR-888-5p
MIMAT0000070	1.84E-06	5.06E-07	5.27	5.46	2.939	hsa-miR-17-5p
MIMAT0015016	2.62E-10	2.91E-11	7.23	15	2.938	hsa-miR-3145-3p

MIMAT0019872	5.99E-11	5.39E-12	7.54	16.6	2.938	hsa-miR-4742-5p
MIMAT0000251	2.04E-08	3.74E-09	6.29	10.2	2.927	hsa-miR-147a
MIMAT0005914	1.74E-13	8.26E-15	8.69	23	2.924	hsa-miR-1262
MIMAT0013516	3.27E-09	5.01E-10	6.69	12.2	2.923	hsa-miR-2681-3p
MIMAT0004507	1.78E-07	3.92E-08	5.81	7.94	2.92	hsa-miR-92a-1-5p
MIMAT0005792	2.28E-23	2.14E-25	12.8	47.1	2.916	hsa-miR-320b
MIMAT0005951	2.44E-43	9.50E-47	21.7	95.7	2.914	hsa-miR-1307-3p
MIMAT0026476	1.82E-08	3.28E-09	6.32	10.4	2.908	hsa-miR-215-3p
MIMAT0027379	4.77E-10	5.73E-11	7.1	14.3	2.907	hsa-miR-6739-5p
MIMAT0019801	2.17E-14	7.88E-16	9.1	25.4	2.907	hsa-miR-4703-5p
MIMAT0000418	3.12E-09	4.77E-10	6.7	12.3	2.907	hsa-miR-23b-3p
MIMAT0000072	2.02E-07	4.50E-08	5.79	7.81	2.902	hsa-miR-18a-5p
MIMAT0018091	7.39E-10	9.45E-11	7	13.8	2.901	hsa-miR-3668
MIMAT0019984	2.58E-08	4.81E-09	6.24	9.99	2.901	hsa-miR-4804-5p
MIMAT0005934	1.39E-10	1.42E-11	7.36	15.7	2.901	hsa-miR-548p
MIMAT0015056	6.90E-13	3.79E-14	8.43	21.5	2.899	hsa-miR-3179
MIMAT0018077	3.04E-09	4.58E-10	6.7	12.3	2.898	hsa-miR-3657
MIMAT0019742	1.47E-07	3.16E-08	5.86	8.15	2.892	hsa-miR-4666a-3p
MIMAT0003247	7.46E-07	1.90E-07	5.48	6.41	2.89	hsa-miR-582-5p
MIMAT0022269	3.33E-12	2.19E-13	8.12	19.8	2.89	hsa-miR-5579-5p
MIMAT0002172	3.07E-07	7.10E-08	5.69	7.37	2.89	hsa-miR-376b-3p
MIMAT0018093	1.84E-08	3.32E-09	6.32	10.4	2.887	hsa-miR-3670
MIMAT0019947	1.30E-41	1.01E-44	20.8	91.1	2.887	hsa-miR-4783-3p
MIMAT0002828	6.62E-11	6.09E-12	7.51	16.5	2.885	hsa-miR-519e-5p
MIMAT0000736	4.81E-07	1.16E-07	5.59	6.89	2.881	hsa-miR-381-3p
MIMAT0018202	1.10E-11	8.29E-13	7.88	18.5	2.88	hsa-miR-3927-3p
MIMAT0003236	3.22E-07	7.46E-08	5.68	7.32	2.879	hsa-miR-571
MIMAT0026736	1.12E-09	1.50E-10	6.92	13.4	2.878	hsa-miR-548e-5p
MIMAT0019029	3.21E-08	6.09E-09	6.19	9.76	2.878	hsa-miR-4494
MIMAT0018097	6.03E-13	3.29E-14	8.45	21.7	2.876	hsa-miR-3674
MIMAT0022928	4.26E-09	6.72E-10	6.63	11.9	2.876	hsa-miR-376a-2-5p
MIMAT0018199	5.98E-14	2.59E-15	8.9	24.2	2.876	hsa-miR-3924
MIMAT0016877	8.27E-11	7.87E-12	7.47	16.3	2.869	hsa-miR-4256
MIMAT0018094	6.51E-10	8.23E-11	7.03	14	2.868	hsa-miR-3671
MIMAT0022265	2.68E-07	6.15E-08	5.72	7.5	2.867	hsa-miR-548ar-5p
MIMAT0022705	3.08E-10	3.47E-11	7.19	14.8	2.864	hsa-miR-539-3p
MIMAT0031003	1.59E-13	7.42E-15	8.71	23.1	2.863	hsa-miR-8076
MIMAT0003885	1.78E-09	2.52E-10	6.82	12.9	2.862	hsa-miR-454-3p
MIMAT0000453	3.94E-08	7.66E-09	6.15	9.54	2.862	hsa-miR-154-3p
MIMAT0015030	6.02E-20	1.22E-21	11.4	38.6	2.859	hsa-miR-3156-5p
MIMAT0004917	6.64E-09	1.10E-09	6.53	11.4	2.858	hsa-miR-888-3p
MIMAT0000253	3.92E-08	7.61E-09	6.15	9.54	2.854	hsa-miR-10a-5p
MIMAT0018095	5.50E-10	6.69E-11	7.07	14.2	2.852	hsa-miR-3672
MIMAT0004803	3.80E-07	8.89E-08	5.64	7.15	2.849	hsa-miR-548a-5p
MIMAT0009978	4.97E-09	8.06E-10	6.59	11.7	2.848	hsa-miR-2053
MIMAT0004809	3.69E-09	5.79E-10	6.66	12.1	2.846	hsa-miR-628-5p
MIMAT0004806, M	1.15E-07	2.43E-08	5.91	8.41	2.842	hsa-miR-548c-5p, hsa-miR-548o-5p, hsa-miR-548am-5p
MIMAT0027581	2.34E-08	4.34E-09	6.26	10.1	2.836	hsa-miR-6839-3p
MIMAT0015012	3.38E-09	5.23E-10	6.68	12.2	2.835	hsa-miR-3143
MIMAT0000272	4.25E-07	1.00E-07	5.62	7.03	2.834	hsa-miR-215-5p
MIMAT0030988	5.32E-10	6.45E-11	7.08	14.2	2.832	hsa-miR-8061
MIMAT0022483	3.12E-09	4.77E-10	6.7	12.3	2.83	hsa-miR-5691
MIMAT0003329	4.15E-07	9.80E-08	5.62	7.05	2.828	hsa-miR-411-5p
MIMAT0018936	1.06E-07	2.23E-08	5.93	8.49	2.822	hsa-miR-4423-3p
MIMAT0019905	1.95E-09	2.77E-10	6.8	12.8	2.821	hsa-miR-4759
MIMAT0000417	1.09E-06	2.87E-07	5.39	6.01	2.819	hsa-miR-15b-5p

MIMAT0000452	3.21E-08	6.09E-09	6.19	9.76	2.817	hsa-miR-154-5p
MIMAT0004774	3.03E-09	4.57E-10	6.7	12.3	2.816	hsa-miR-501-3p
MIMAT0021084	5.95E-08	1.19E-08	6.06	9.11	2.816	hsa-miR-5092
MIMAT0018972	2.34E-16	6.58E-18	9.92	30.1	2.815	hsa-miR-548ah-5p
MIMAT0017986	1.43E-10	1.49E-11	7.35	15.6	2.809	hsa-miR-3609
MIMAT0004689	3.73E-08	7.22E-09	6.16	9.59	2.806	hsa-miR-377-5p
MIMAT0019040	2.04E-09	2.95E-10	6.79	12.7	2.804	hsa-miR-4504
MIMAT0022279	1.38E-08	2.44E-09	6.38	10.7	2.8	hsa-miR-5582-5p
MIMAT0022270	7.03E-11	6.50E-12	7.5	16.5	2.8	hsa-miR-5579-3p
MIMAT0003219	8.29E-08	1.72E-08	5.98	8.75	2.799	hsa-miR-555
MIMAT0019974	3.87E-10	4.46E-11	7.15	14.6	2.799	hsa-miR-4798-5p
MIMAT0000646	5.34E-07	1.31E-07	5.56	6.77	2.797	hsa-miR-155-5p
MIMAT0005791	1.29E-09	1.76E-10	6.89	13.2	2.795	hsa-miR-1264
MIMAT0004562	4.66E-07	1.12E-07	5.59	6.92	2.786	hsa-miR-196a-3p
MIMAT0004566	2.57E-08	4.79E-09	6.24	9.99	2.78	hsa-miR-218-2-3p
MIMAT0000273	1.94E-06	5.39E-07	5.25	5.4	2.779	hsa-miR-216a-5p
MIMAT0011157	1.18E-06	3.13E-07	5.37	5.92	2.778	hsa-miR-2114-3p
MIMAT0019971	6.00E-08	1.20E-08	6.06	9.1	2.778	hsa-miR-4796-3p
MIMAT0005933	5.89E-13	3.17E-14	8.46	21.7	2.776	hsa-miR-1277-3p
MIMAT0003286	6.26E-08	1.26E-08	6.05	9.05	2.775	hsa-miR-617
MIMAT0019951	9.20E-11	8.83E-12	7.45	16.2	2.773	hsa-miR-1245b-3p
MIMAT0000103	4.27E-06	1.27E-06	5.06	4.57	2.771	hsa-miR-106a-5p
MIMAT0003226	1.73E-08	3.10E-09	6.33	10.4	2.771	hsa-miR-562
MIMAT0019918	3.10E-09	4.71E-10	6.7	12.3	2.77	hsa-miR-4766-3p
MIMAT0002873	5.93E-07	1.47E-07	5.54	6.66	2.769	hsa-miR-502-5p
MIMAT0000458	2.06E-08	3.79E-09	6.29	10.2	2.766	hsa-miR-190a-5p
MIMAT0000715	3.41E-07	7.92E-08	5.67	7.26	2.763	hsa-miR-302b-3p
MIMAT0022482	5.79E-07	1.43E-07	5.54	6.69	2.762	hsa-miR-5690
MIMAT0000269	2.01E-09	2.90E-10	6.79	12.7	2.759	hsa-miR-212-3p
MIMAT0021026	7.20E-09	1.20E-09	6.52	11.3	2.748	hsa-miR-5003-3p
MIMAT0019197	1.05E-10	1.01E-11	7.42	16	2.748	hsa-miR-3117-5p
MIMAT0002827	5.37E-06	1.65E-06	5	4.32	2.746	hsa-miR-515-3p
MIMAT0000085	2.55E-06	7.29E-07	5.19	5.11	2.746	hsa-miR-28-5p
MIMAT0018998	6.67E-08	1.35E-08	6.03	8.98	2.745	hsa-miR-4471
MIMAT0015037	4.76E-09	7.65E-10	6.6	11.8	2.741	hsa-miR-3163
MIMAT0000074	4.68E-06	1.41E-06	5.04	4.47	2.74	hsa-miR-19b-3p
MIMAT0000104	5.15E-06	1.57E-06	5.02	4.37	2.735	hsa-miR-107
MIMAT0003271	5.79E-08	1.15E-08	6.07	9.14	2.732	hsa-miR-603
MIMAT0003150	6.69E-10	8.51E-11	7.02	13.9	2.732	hsa-miR-455-5p
MIMAT0003321	9.34E-07	2.42E-07	5.43	6.17	2.73	hsa-miR-651-5p
MIMAT0004925	4.34E-09	6.88E-10	6.62	11.9	2.727	hsa-miR-876-3p
MIMAT0022975	2.80E-08	5.28E-09	6.22	9.9	2.726	hsa-miR-3934-3p
MIMAT0000090	2.65E-06	7.62E-07	5.18	5.06	2.722	hsa-miR-32-5p
MIMAT0000448	5.75E-07	1.42E-07	5.54	6.69	2.72	hsa-miR-136-5p
MIMAT0000705	2.49E-06	7.07E-07	5.19	5.14	2.719	hsa-miR-362-5p
MIMAT0002881	7.11E-07	1.81E-07	5.49	6.46	2.718	hsa-miR-509-3p
MIMAT0030993	2.49E-09	3.67E-10	6.75	12.5	2.717	hsa-miR-8066
MIMAT0030983	4.37E-06	1.31E-06	5.06	4.54	2.715	hsa-miR-8056
MIMAT0019969	1.86E-05	6.42E-06	4.69	3.01	2.714	hsa-miR-4795-3p
MIMAT0000432	5.81E-10	7.14E-11	7.06	14.1	2.714	hsa-miR-141-3p
MIMAT0032114, M	2.49E-10	2.74E-11	7.24	15.1	2.708	hsa-miR-548ad-5p, hsa-miR-548ae-5p
MIMAT0018358	2.41E-09	3.54E-10	6.75	12.5	2.703	hsa-miR-3942-5p
MIMAT0018081	9.66E-08	2.02E-08	5.95	8.59	2.703	hsa-miR-3660
MIMAT0018988	8.87E-13	5.12E-14	8.37	21.2	2.697	hsa-miR-4464
MIMAT0014981	6.61E-10	8.38E-11	7.03	14	2.697	hsa-miR-3119
MIMAT0005945	1.08E-10	1.05E-11	7.41	16	2.695	hsa-miR-1255b-5p
MIMAT0001541	2.22E-06	6.23E-07	5.22	5.26	2.695	hsa-miR-449a

MIMAT0022728	5.78E-11	5.09E-12	7.55	16.7	2.693	hsa-miR-513c-3p
MIMAT0014980	4.54E-13	2.41E-14	8.51	22	2.692	hsa-miR-3118
MIMAT0027677	2.58E-08	4.83E-09	6.24	9.99	2.691	hsa-miR-6888-3p
MIMAT0015042	3.40E-09	5.27E-10	6.68	12.2	2.686	hsa-miR-3167
MIMAT0018982	2.21E-05	7.80E-06	4.64	2.82	2.682	hsa-miR-4460
MIMAT0019004	6.94E-07	1.75E-07	5.5	6.49	2.68	hsa-miR-4477a
MIMAT0025851	2.58E-08	4.84E-09	6.24	9.98	2.679	hsa-miR-6720-3p
MIMAT0024615	2.49E-08	4.62E-09	6.25	10	2.679	hsa-miR-6131
MIMAT0000258	1.32E-06	3.53E-07	5.35	5.81	2.679	hsa-miR-181c-5p
MIMAT0026625	1.87E-10	2.01E-11	7.29	15.4	2.677	hsa-miR-653-3p
MIMAT0004686	1.36E-06	3.66E-07	5.34	5.77	2.675	hsa-miR-367-5p
MIMAT0000068	9.07E-06	2.91E-06	4.87	3.77	2.674	hsa-miR-15a-5p
MIMAT0000243	5.64E-08	1.12E-08	6.07	9.17	2.671	hsa-miR-148a-3p
MIMAT0022267	2.11E-05	7.39E-06	4.66	2.87	2.67	hsa-miR-548as-5p
MIMAT0022303	3.07E-10	3.45E-11	7.19	14.8	2.67	hsa-miR-548av-5p
MIMAT0027629	5.42E-06	1.67E-06	5	4.31	2.665	hsa-miR-6864-3p
MIMAT0022304	1.43E-08	2.55E-09	6.37	10.6	2.664	hsa-miR-548av-3p
MIMAT0003303	9.51E-07	2.47E-07	5.42	6.15	2.662	hsa-miR-633
MIMAT0003275	1.18E-08	2.05E-09	6.41	10.8	2.661	hsa-miR-607
MIMAT0004956	3.66E-09	5.72E-10	6.66	12.1	2.661	hsa-miR-374b-3p
MIMAT0002831, M	4.81E-07	1.16E-07	5.59	6.89	2.652	hsa-miR-519c-5p, hsa-miR-523-5p, hsa-miR-518e-5p, hsa-miR-522-5p, hsa-miR-519a-5p, hsa-miR-519b-5p
MIMAT0019824	5.68E-09	9.27E-10	6.57	11.6	2.65	hsa-miR-4715-5p
MIMAT0004551	1.44E-07	3.10E-08	5.86	8.17	2.648	hsa-miR-30d-3p
MIMAT0004812	1.31E-07	2.78E-08	5.89	8.28	2.644	hsa-miR-548d-5p
MIMAT0000431	2.41E-06	6.84E-07	5.2	5.17	2.644	hsa-miR-140-5p
MIMAT0027589	4.89E-07	1.19E-07	5.58	6.87	2.641	hsa-miR-6844
MIMAT0003268	4.66E-07	1.12E-07	5.59	6.92	2.641	hsa-miR-600
MIMAT0018183	3.31E-06	9.70E-07	5.12	4.83	2.637	hsa-miR-3909
MIMAT0004752	2.42E-07	5.47E-08	5.75	7.62	2.637	hsa-miR-20b-3p
MIMAT0015009	1.70E-07	3.70E-08	5.83	8	2.636	hsa-miR-548t-5p
MIMAT0004797	6.51E-07	1.62E-07	5.52	6.56	2.636	hsa-miR-582-3p
MIMAT0005930	9.01E-08	1.87E-08	5.97	8.66	2.635	hsa-miR-1276
MIMAT0004491	1.86E-06	5.15E-07	5.26	5.44	2.63	hsa-miR-19b-1-5p
MIMAT0022288	1.24E-06	3.31E-07	5.36	5.87	2.626	hsa-miR-5586-3p
MIMAT0019051	2.05E-06	5.71E-07	5.24	5.34	2.626	hsa-miR-4514
MIMAT0005895	1.03E-05	3.31E-06	4.84	3.65	2.624	hsa-miR-548f-3p
MIMAT0019960	1.51E-07	3.26E-08	5.85	8.12	2.62	hsa-miR-4789-3p
MIMAT0027571	1.74E-05	5.90E-06	4.71	3.09	2.617	hsa-miR-6835-3p
MIMAT0015007	3.28E-06	9.59E-07	5.13	4.84	2.615	hsa-miR-3139
MIMAT0011159	4.93E-07	1.20E-07	5.58	6.86	2.615	hsa-miR-2115-3p
MIMAT0003296	9.70E-08	2.03E-08	5.95	8.58	2.614	hsa-miR-627-5p
MIMAT0027664	3.60E-08	6.91E-09	6.17	9.64	2.613	hsa-miR-6882-5p
MIMAT0021023	8.53E-10	1.10E-10	6.98	13.7	2.612	hsa-miR-5002-5p
MIMAT0004549	3.18E-07	7.35E-08	5.68	7.33	2.611	hsa-miR-148a-5p
MIMAT0003274	1.29E-10	1.29E-11	7.38	15.8	2.609	hsa-miR-606
MIMAT0019762	1.04E-09	1.37E-10	6.93	13.5	2.601	hsa-miR-4678
MIMAT0004772	2.05E-08	3.76E-09	6.29	10.2	2.601	hsa-miR-499a-3p
MIMAT0022696	2.35E-08	4.36E-09	6.26	10.1	2.6	hsa-miR-301a-5p
MIMAT0000437	2.55E-06	7.28E-07	5.19	5.11	2.599	hsa-miR-145-5p
MIMAT0026610	4.46E-07	1.06E-07	5.61	6.98	2.593	hsa-miR-519d-5p
MIMAT0003322	1.65E-06	4.52E-07	5.29	5.57	2.591	hsa-miR-652-3p
MIMAT0019916	2.16E-07	4.88E-08	5.77	7.73	2.587	hsa-miR-4765
MIMAT0030020	1.85E-07	4.09E-08	5.81	7.9	2.581	hsa-miR-7705
MIMAT0019036	2.42E-07	5.49E-08	5.74	7.62	2.581	hsa-miR-4500

MIMAT0027580	3.20E-11	2.66E-12	7.67	17.3	2.58	hsa-miR-6839-5p
MIMAT0019936	9.31E-09	1.58E-09	6.46	11.1	2.577	hsa-miR-4778-5p
MIMAT0000720	2.64E-06	7.56E-07	5.18	5.07	2.575	hsa-miR-376c-3p
MIMAT0000691	7.47E-06	2.36E-06	4.92	3.97	2.575	hsa-miR-130b-3p
MIMAT0021036	5.29E-08	1.04E-08	6.09	9.24	2.571	hsa-miR-5007-3p
MIMAT0003223	2.37E-07	5.35E-08	5.75	7.64	2.569	hsa-miR-559
MIMAT0018969	9.29E-09	1.57E-09	6.46	11.1	2.569	hsa-miR-548ag
MIMAT0019879	5.60E-07	1.38E-07	5.55	6.72	2.568	hsa-miR-4745-3p
MIMAT0019944	3.74E-11	3.16E-12	7.63	17.2	2.568	hsa-miR-4782-5p
MIMAT0019833	3.33E-09	5.14E-10	6.68	12.2	2.563	hsa-miR-4720-5p
MIMAT0002874	8.02E-07	2.06E-07	5.46	6.33	2.561	hsa-miR-503-5p
MIMAT0016912	4.94E-07	1.20E-07	5.58	6.85	2.558	hsa-miR-4282
MIMAT0018184	4.57E-10	5.45E-11	7.11	14.4	2.555	hsa-miR-3910
MIMAT0003289	7.93E-11	7.45E-12	7.48	16.3	2.554	hsa-miR-620
MIMAT0004907	3.06E-07	7.07E-08	5.69	7.37	2.549	hsa-miR-892a
MIMAT0005917	2.00E-09	2.87E-10	6.79	12.7	2.549	hsa-miR-548m
MIMAT0000424	1.77E-05	6.07E-06	4.7	3.06	2.548	hsa-miR-128-3p
MIMAT0019968	1.18E-09	1.59E-10	6.91	13.3	2.548	hsa-miR-4795-5p
MIMAT0019752	1.47E-06	3.97E-07	5.32	5.7	2.547	hsa-miR-4671-5p
MIMAT0018114	3.40E-13	1.76E-14	8.56	22.3	2.546	hsa-miR-3686
MIMAT0001545	1.03E-06	2.68E-07	5.41	6.07	2.539	hsa-miR-450a-5p
MIMAT0021018	2.11E-10	2.28E-11	7.27	15.2	2.538	hsa-miR-4999-3p
MIMAT0022302	2.74E-06	7.90E-07	5.17	5.03	2.535	hsa-miR-5591-3p
MIMAT0005916	6.28E-10	7.85E-11	7.04	14	2.531	hsa-miR-548n
MIMAT0026737	5.81E-08	1.16E-08	6.07	9.13	2.53	hsa-miR-548j-3p
MIMAT0027628	1.86E-06	5.15E-07	5.26	5.44	2.529	hsa-miR-6864-5p
MIMAT0004921	1.39E-10	1.43E-11	7.36	15.7	2.528	hsa-miR-889-3p
MIMAT0003292	1.14E-08	1.97E-09	6.42	10.9	2.525	hsa-miR-623
MIMAT0025480	2.90E-09	4.31E-10	6.71	12.3	2.52	hsa-miR-6512-5p
MIMAT0003225	5.71E-10	6.99E-11	7.06	14.1	2.515	hsa-miR-561-3p
MIMAT0019925	1.75E-05	5.95E-06	4.71	3.08	2.513	hsa-miR-4771
MIMAT0005912	2.00E-06	5.56E-07	5.25	5.37	2.511	hsa-miR-548g-3p
MIMAT0019963	1.51E-07	3.27E-08	5.85	8.12	2.511	hsa-miR-4791
MIMAT0030989	4.25E-07	1.00E-07	5.62	7.03	2.51	hsa-miR-8062
MIMAT0018447, M	3.31E-08	6.32E-09	6.19	9.72	2.505	hsa-miR-548aa, hsa-miR-548t-3p
MIMAT0005885	4.65E-06	1.40E-06	5.04	4.48	2.504	hsa-miR-1295a
MIMAT0019361	6.85E-06	2.16E-06	4.94	4.06	2.504	hsa-miR-3976
MIMAT0015044	1.38E-07	2.94E-08	5.87	8.22	2.503	hsa-miR-3169
MIMAT0002833	2.20E-05	7.76E-06	4.64	2.83	2.502	hsa-miR-520a-5p
MIMAT0011156	3.86E-07	9.05E-08	5.64	7.13	2.502	hsa-miR-2114-5p
MIMAT0021019	1.95E-09	2.78E-10	6.8	12.8	2.501	hsa-miR-5000-5p
MIMAT0018946	1.05E-09	1.40E-10	6.93	13.5	2.501	hsa-miR-548ad-3p
MIMAT0019931	5.80E-11	5.18E-12	7.54	16.7	2.499	hsa-miR-4775
MIMAT0015045	1.53E-06	4.16E-07	5.31	5.65	2.498	hsa-miR-3170
MIMAT0014991	1.56E-09	2.16E-10	6.85	13	2.495	hsa-miR-3128
MIMAT0016879	2.28E-38	2.72E-41	19.3	83.3	2.493	hsa-miR-4258
MIMAT0018353	2.08E-06	5.80E-07	5.24	5.33	2.492	hsa-miR-3938
MIMAT0004613	6.42E-06	2.01E-06	4.96	4.13	2.491	hsa-miR-188-3p
MIMAT0000763	4.77E-06	1.44E-06	5.03	4.45	2.483	hsa-miR-338-3p
MIMAT0004553	6.94E-07	1.76E-07	5.5	6.48	2.473	hsa-miR-7-1-3p
MIMAT0019910	8.02E-06	2.54E-06	4.91	3.9	2.471	hsa-miR-4762-5p
MIMAT0000422	6.66E-06	2.09E-06	4.95	4.09	2.47	hsa-miR-124-3p
MIMAT0005894	1.37E-08	2.41E-09	6.38	10.7	2.469	hsa-miR-1243
MIMAT0022469	1.42E-07	3.03E-08	5.87	8.19	2.468	hsa-miR-5681a
MIMAT0022470	1.17E-08	2.04E-09	6.41	10.8	2.468	hsa-miR-5682
MIMAT0020300	9.02E-07	2.33E-07	5.44	6.21	2.467	hsa-miR-4520-2-3p
MIMAT0004922	6.22E-11	5.65E-12	7.53	16.6	2.467	hsa-miR-875-5p

MIMAT0004765	5.34E-07	1.31E-07	5.56	6.77	2.466	hsa-miR-491-3p
MIMAT0003164	2.08E-07	4.65E-08	5.78	7.78	2.462	hsa-miR-544a
MIMAT0002863	8.42E-06	2.68E-06	4.89	3.85	2.459	hsa-miR-518a-3p
MIMAT0027646	4.01E-06	1.19E-06	5.08	4.63	2.458	hsa-miR-6873-5p
MIMAT0014977	4.00E-10	4.64E-11	7.14	14.5	2.458	hsa-miR-3115
MIMAT0005799	4.69E-07	1.13E-07	5.59	6.91	2.457	hsa-miR-1283
MIMAT0019796	6.04E-06	1.88E-06	4.97	4.19	2.457	hsa-miR-4700-5p
MIMAT0009979	2.48E-07	5.64E-08	5.74	7.59	2.456	hsa-miR-2054
MIMAT0002856	1.28E-05	4.22E-06	4.79	3.41	2.455	hsa-miR-520d-3p
MIMAT0004948	1.16E-21	1.54E-23	12.1	42.9	2.455	hsa-miR-885-3p
MIMAT0017990	1.09E-10	1.08E-11	7.41	16	2.455	hsa-miR-3613-5p
MIMAT0014978	1.37E-06	3.69E-07	5.34	5.76	2.452	hsa-miR-3116
MIMAT0019864	3.53E-06	1.04E-06	5.11	4.76	2.451	hsa-miR-3064-5p
MIMAT0019972	1.35E-05	4.50E-06	4.77	3.35	2.45	hsa-miR-4797-5p
MIMAT0000510	5.25E-22	6.34E-24	12.3	43.8	2.449	hsa-miR-320a
MIMAT0019746	5.12E-06	1.55E-06	5.02	4.37	2.449	hsa-miR-4668-3p
MIMAT0000415	4.38E-06	1.31E-06	5.06	4.54	2.447	hsa-let-7i-5p
MIMAT0019360	1.06E-07	2.23E-08	5.93	8.49	2.447	hsa-miR-3975
MIMAT0018355	1.82E-08	3.29E-09	6.32	10.4	2.446	hsa-miR-3939
MIMAT0004924	6.73E-07	1.69E-07	5.51	6.52	2.445	hsa-miR-876-5p
MIMAT0015000	7.17E-08	1.47E-08	6.02	8.9	2.441	hsa-miR-3134
MIMAT0019896	1.13E-04	4.76E-05	4.2	1.09	2.438	hsa-miR-4755-3p
MIMAT0019765	6.53E-07	1.63E-07	5.51	6.56	2.436	hsa-miR-4680-3p
MIMAT0026626	9.52E-08	1.99E-08	5.95	8.61	2.436	hsa-miR-655-5p
MIMAT0004960	2.87E-06	8.31E-07	5.16	4.98	2.435	hsa-miR-208b-3p
MIMAT0030424	3.36E-06	9.88E-07	5.12	4.81	2.434	hsa-miR-7849-3p
MIMAT0003165	6.48E-07	1.61E-07	5.52	6.57	2.433	hsa-miR-545-3p
MIMAT0001635	1.14E-05	3.73E-06	4.82	3.53	2.433	hsa-miR-452-5p
MIMAT0019895	6.71E-07	1.68E-07	5.51	6.53	2.432	hsa-miR-4755-5p
MIMAT0000433	3.02E-05	1.10E-05	4.56	2.49	2.431	hsa-miR-142-5p
MIMAT0004599	9.96E-07	2.59E-07	5.41	6.11	2.428	hsa-miR-143-5p
MIMAT0000721	1.67E-05	5.67E-06	4.72	3.13	2.426	hsa-miR-369-3p
MIMAT0004785	6.91E-07	1.74E-07	5.5	6.49	2.425	hsa-miR-545-5p
MIMAT0022300	4.29E-08	8.40E-09	6.13	9.45	2.422	hsa-miR-5590-3p
MIMAT0018186	1.42E-10	1.48E-11	7.35	15.7	2.42	hsa-miR-3912-3p
MIMAT0026719	9.51E-06	3.05E-06	4.86	3.72	2.42	hsa-miR-889-5p
MIMAT0015011	5.23E-06	1.60E-06	5.01	4.35	2.419	hsa-miR-3142
MIMAT0019823	4.30E-07	1.02E-07	5.61	7.01	2.419	hsa-miR-4714-3p
MIMAT0004567	4.91E-07	1.19E-07	5.58	6.86	2.419	hsa-miR-219a-1-3p
MIMAT0014987	5.51E-05	2.12E-05	4.4	1.86	2.416	hsa-miR-548s
MIMAT0003284	2.68E-06	7.70E-07	5.18	5.05	2.416	hsa-miR-616-5p
MIMAT0002813	5.12E-06	1.56E-06	5.02	4.37	2.415	hsa-miR-493-5p
MIMAT0019728	1.79E-06	4.90E-07	5.28	5.49	2.413	hsa-miR-4660
MIMAT0005955	2.85E-08	5.38E-09	6.22	9.88	2.411	hsa-miR-1197
MIMAT0021030	1.22E-06	3.25E-07	5.37	5.89	2.41	hsa-miR-548ao-3p
MIMAT0005798	7.75E-08	1.60E-08	6	8.82	2.406	hsa-miR-1185-5p
MIMAT0004974	1.10E-08	1.89E-09	6.43	10.9	2.405	hsa-miR-924
MIMAT0019784	2.00E-09	2.86E-10	6.79	12.8	2.401	hsa-miR-4693-5p
MIMAT0027036	4.46E-07	1.06E-07	5.61	6.98	2.401	hsa-miR-3912-5p
MIMAT0005944	7.89E-07	2.02E-07	5.47	6.35	2.401	hsa-miR-1252-5p
MIMAT0001340	2.25E-08	4.14E-09	6.27	10.1	2.398	hsa-miR-423-3p
MIMAT0019934	1.68E-07	3.64E-08	5.83	8.02	2.398	hsa-miR-4777-5p
MIMAT0028233	3.00E-06	8.73E-07	5.15	4.93	2.396	hsa-miR-7161-3p
MIMAT0022271	1.69E-07	3.69E-08	5.83	8	2.396	hsa-miR-664b-5p
MIMAT0027410	3.04E-09	4.60E-10	6.7	12.3	2.394	hsa-miR-6755-5p
MIMAT0019898	6.18E-06	1.93E-06	4.97	4.17	2.392	hsa-miR-499b-3p
MIMAT0016861	1.31E-06	3.52E-07	5.35	5.81	2.39	hsa-miR-4308
MIMAT0003259	2.22E-06	6.24E-07	5.22	5.26	2.39	hsa-miR-591

MIMAT0018963	7.51E-05	3.02E-05	4.31	1.52	2.388	hsa-miR-4445-5p
MIMAT0000091	1.83E-05	6.30E-06	4.69	3.03	2.385	hsa-miR-33a-5p
MIMAT0000441	2.14E-05	7.51E-06	4.65	2.86	2.382	hsa-miR-9-5p
MIMAT0000100	3.77E-05	1.41E-05	4.5	2.25	2.381	hsa-miR-29b-3p
MIMAT0002876	1.80E-05	6.18E-06	4.7	3.04	2.381	hsa-miR-505-3p
MIMAT0004764	1.31E-06	3.50E-07	5.35	5.82	2.38	hsa-miR-490-5p
MIMAT0019212	4.27E-08	8.33E-09	6.13	9.45	2.379	hsa-miR-3160-5p
MIMAT0031176	5.18E-06	1.58E-06	5.01	4.36	2.377	hsa-miR-7973
MIMAT0015013	4.85E-09	7.84E-10	6.6	11.8	2.375	hsa-miR-548u
MIMAT0004929	3.71E-07	8.66E-08	5.65	7.17	2.374	hsa-miR-190b
MIMAT0021086	6.66E-06	2.09E-06	4.95	4.09	2.374	hsa-miR-5094
MIMAT0030992	6.94E-07	1.76E-07	5.5	6.48	2.374	hsa-miR-8065
MIMAT0006767	2.74E-07	6.30E-08	5.72	7.48	2.372	hsa-miR-1827
MIMAT0018357	1.80E-08	3.24E-09	6.32	10.4	2.371	hsa-miR-3941
MIMAT0000416	1.25E-05	4.11E-06	4.79	3.44	2.37	hsa-miR-1-3p
MIMAT0019891	1.07E-08	1.84E-09	6.43	10.9	2.364	hsa-miR-4753-3p
MIMAT0015033	8.39E-06	2.67E-06	4.89	3.85	2.362	hsa-miR-3159
MIMAT0019714	1.91E-05	6.64E-06	4.68	2.98	2.362	hsa-miR-4650-3p
MIMAT0005897	2.00E-05	6.97E-06	4.67	2.93	2.359	hsa-miR-1245a
MIMAT0019071	3.20E-33	1.25E-35	16.9	70.5	2.359	hsa-miR-4532
MIMAT0019016	9.65E-09	1.64E-09	6.45	11	2.358	hsa-miR-4482-5p
MIMAT0018201	3.03E-06	8.82E-07	5.15	4.92	2.358	hsa-miR-3926
MIMAT0022498	3.18E-06	9.30E-07	5.13	4.87	2.357	hsa-miR-5704
MIMAT0022277	2.09E-07	4.69E-08	5.78	7.77	2.357	hsa-miR-548at-5p
MIMAT0019220	2.37E-07	5.35E-08	5.75	7.64	2.356	hsa-miR-3664-3p
MIMAT0018973	7.35E-06	2.32E-06	4.93	3.99	2.352	hsa-miR-4451
MIMAT0019067	1.77E-05	6.04E-06	4.7	3.07	2.351	hsa-miR-4528
MIMAT0019734	2.65E-07	6.07E-08	5.72	7.52	2.35	hsa-miR-4659b-3p
MIMAT0016848	6.61E-06	2.07E-06	4.95	4.1	2.349	hsa-miR-4293
MIMAT0000714	2.70E-05	9.75E-06	4.59	2.61	2.345	hsa-miR-302b-5p
MIMAT0018954	4.56E-07	1.09E-07	5.6	6.95	2.345	hsa-miR-548ae-3p
MIMAT0019062	2.96E-06	8.59E-07	5.15	4.95	2.344	hsa-miR-4524a-5p
MIMAT0016853	2.16E-06	6.03E-07	5.23	5.29	2.341	hsa-miR-4300
MIMAT0005886	5.18E-07	1.27E-07	5.57	6.8	2.341	hsa-miR-1297
MIMAT0019977	2.58E-07	5.89E-08	5.73	7.55	2.34	hsa-miR-4799-3p
MIMAT0019013	7.04E-08	1.43E-08	6.02	8.92	2.339	hsa-miR-548ak
MIMAT0016903	2.59E-05	9.30E-06	4.6	2.65	2.338	hsa-miR-4273
MIMAT0014985	7.56E-07	1.93E-07	5.48	6.39	2.336	hsa-miR-3123
MIMAT0005896	1.97E-08	3.60E-09	6.3	10.3	2.336	hsa-miR-1244
MIMAT0018203	1.86E-06	5.15E-07	5.26	5.44	2.332	hsa-miR-676-5p
MIMAT0030981	1.26E-05	4.13E-06	4.79	3.43	2.329	hsa-miR-8054
MIMAT0004512	4.18E-06	1.25E-06	5.07	4.59	2.328	hsa-miR-100-3p
MIMAT0022468	2.53E-07	5.77E-08	5.73	7.57	2.327	hsa-miR-5680
MIMAT0002875	1.35E-05	4.49E-06	4.77	3.35	2.327	hsa-miR-504-5p
MIMAT0027517	2.50E-06	7.14E-07	5.19	5.13	2.326	hsa-miR-6808-3p
MIMAT0000445	1.17E-04	4.94E-05	4.19	1.06	2.325	hsa-miR-126-3p
MIMAT0025469	5.76E-06	1.79E-06	4.99	4.24	2.323	hsa-miR-6506-3p
MIMAT0019943	1.12E-08	1.93E-09	6.42	10.9	2.323	hsa-miR-4781-3p
MIMAT0000680	6.81E-05	2.70E-05	4.34	1.63	2.323	hsa-miR-106b-5p
MIMAT0004510	5.77E-06	1.79E-06	4.99	4.24	2.322	hsa-miR-96-3p
MIMAT0000737	3.36E-05	1.24E-05	4.53	2.38	2.321	hsa-miR-382-5p
MIMAT0020541	3.91E-08	7.57E-09	6.15	9.55	2.321	hsa-miR-5047
MIMAT0021045	1.35E-05	4.50E-06	4.77	3.35	2.32	hsa-miR-5011-5p
MIMAT0000681	8.20E-05	3.33E-05	4.29	1.43	2.318	hsa-miR-29c-3p
MIMAT0016905	1.41E-05	4.71E-06	4.76	3.3	2.317	hsa-miR-4275
MIMAT0004923	4.70E-06	1.42E-06	5.04	4.46	2.312	hsa-miR-875-3p
MIMAT0003386	9.99E-07	2.60E-07	5.41	6.1	2.31	hsa-miR-376a-5p
MIMAT0022924	2.12E-07	4.76E-08	5.77	7.75	2.308	hsa-miR-495-5p

MIMAT0001625	2.40E-05	8.55E-06	4.62	2.73	2.308	hsa-miR-431-5p
MIMAT0022292	9.37E-08	1.95E-08	5.96	8.62	2.307	hsa-miR-548au-3p
MIMAT0004568	2.33E-08	4.31E-09	6.26	10.1	2.3	hsa-miR-221-5p
MIMAT0001412	1.93E-04	8.63E-05	4.04	0.52	2.3	hsa-miR-18b-5p
MIMAT0004612	8.96E-06	2.87E-06	4.88	3.78	2.299	hsa-miR-186-3p
MIMAT0016850	5.28E-06	1.62E-06	5.01	4.34	2.298	hsa-miR-4301
MIMAT0019876	1.67E-04	7.36E-05	4.09	0.68	2.297	hsa-miR-3591-5p
MIMAT0011160	1.06E-06	2.79E-07	5.4	6.04	2.297	hsa-miR-2116-5p
MIMAT0003265	1.80E-05	6.16E-06	4.7	3.05	2.297	hsa-miR-597-5p
MIMAT0027435	8.80E-07	2.27E-07	5.44	6.24	2.297	hsa-miR-6767-3p
MIMAT0004513	1.35E-05	4.50E-06	4.77	3.35	2.296	hsa-miR-101-5p
MIMAT0003301	5.78E-05	2.23E-05	4.39	1.81	2.295	hsa-miR-33b-5p
MIMAT0022299	1.53E-06	4.15E-07	5.31	5.65	2.294	hsa-miR-5590-5p
MIMAT0018108	1.20E-06	3.18E-07	5.37	5.91	2.292	hsa-miR-3681-5p
MIMAT0027534	5.39E-06	1.65E-06	5	4.31	2.29	hsa-miR-6817-5p
MIMAT0019900	8.96E-06	2.86E-06	4.88	3.79	2.286	hsa-miR-4756-3p
MIMAT0000077	1.86E-05	6.41E-06	4.69	3.01	2.285	hsa-miR-22-3p
MIMAT0018957	7.56E-07	1.93E-07	5.48	6.39	2.282	hsa-miR-4439
MIMAT0003318	1.32E-05	4.36E-06	4.78	3.38	2.282	hsa-miR-648
MIMAT0003216	5.28E-06	1.62E-06	5.01	4.34	2.282	hsa-miR-553
MIMAT0000439	1.15E-05	3.74E-06	4.82	3.53	2.282	hsa-miR-153-3p
MIMAT0000759	6.92E-05	2.74E-05	4.34	1.62	2.281	hsa-miR-148b-3p
MIMAT0004796	1.28E-08	2.25E-09	6.39	10.7	2.279	hsa-miR-576-3p
MIMAT0005887	2.73E-05	9.86E-06	4.59	2.6	2.277	hsa-miR-1299
MIMAT0018190	1.09E-06	2.85E-07	5.39	6.02	2.276	hsa-miR-3916
MIMAT0019697	5.54E-06	1.71E-06	5	4.28	2.274	hsa-miR-4639-5p
MIMAT0019019	5.39E-05	2.06E-05	4.41	1.89	2.273	hsa-miR-4485-3p
MIMAT0000087	6.26E-05	2.45E-05	4.36	1.72	2.272	hsa-miR-30a-5p
MIMAT0003282	6.97E-19	1.55E-20	10.9	36.1	2.271	hsa-miR-614
MIMAT0018354	8.11E-08	1.68E-08	5.99	8.77	2.271	hsa-miR-548y
MIMAT0019907	6.68E-07	1.67E-07	5.51	6.53	2.27	hsa-miR-4760-3p
MIMAT0004777	6.82E-06	2.15E-06	4.94	4.06	2.267	hsa-miR-513a-3p
MIMAT0015025	4.73E-06	1.43E-06	5.04	4.46	2.265	hsa-miR-3152-3p
MIMAT0019228	4.05E-07	9.53E-08	5.63	7.08	2.263	hsa-miR-3925-3p
MIMAT0004556	1.93E-07	4.28E-08	5.8	7.86	2.263	hsa-miR-10b-3p
MIMAT0000685	1.46E-05	4.90E-06	4.75	3.27	2.262	hsa-miR-34b-5p
MIMAT0019794	5.92E-05	2.31E-05	4.38	1.78	2.262	hsa-miR-4699-5p
MIMAT0025473	2.17E-06	6.07E-07	5.23	5.28	2.256	hsa-miR-6508-3p
MIMAT0018117, M	5.31E-08	1.05E-08	6.09	9.23	2.256	hsa-miR-3689a-5p, hsa-miR-3689b-5p, hsa-miR-3689e
MIMAT0004987	1.38E-04	5.92E-05	4.14	0.88	2.251	hsa-miR-944
MIMAT0018974	1.96E-07	4.36E-08	5.79	7.84	2.25	hsa-miR-4452
MIMAT0022471	1.50E-08	2.67E-09	6.36	10.6	2.248	hsa-miR-548aw
MIMAT0005935	5.95E-08	1.19E-08	6.06	9.11	2.24	hsa-miR-548i
MIMAT0000267	3.33E-12	2.17E-13	8.12	19.8	2.239	hsa-miR-210-3p
MIMAT0026615	1.57E-05	5.30E-06	4.73	3.19	2.236	hsa-miR-552-5p
MIMAT0019945	3.68E-06	1.09E-06	5.1	4.72	2.236	hsa-miR-4782-3p
MIMAT0022291	3.83E-09	6.03E-10	6.65	12	2.236	hsa-miR-548au-5p
MIMAT0001080	1.90E-05	6.58E-06	4.68	2.98	2.232	hsa-miR-196b-5p
MIMAT0026609	1.31E-05	4.31E-06	4.78	3.39	2.232	hsa-miR-520f-5p
MIMAT0018000	3.87E-05	1.45E-05	4.49	2.23	2.231	hsa-miR-23c
MIMAT0000761	2.20E-05	7.76E-06	4.64	2.83	2.229	hsa-miR-324-5p
MIMAT0005870	7.93E-07	2.04E-07	5.47	6.34	2.229	hsa-miR-1206
MIMAT0004763	1.07E-06	2.80E-07	5.4	6.03	2.229	hsa-miR-488-3p
MIMAT0000729	1.24E-04	5.29E-05	4.17	0.99	2.229	hsa-miR-376a-3p
MIMAT0022978	5.43E-06	1.67E-06	5	4.3	2.225	hsa-miR-4743-3p
MIMAT0003232	1.59E-05	5.36E-06	4.73	3.18	2.22	hsa-miR-568

MIMAT0026739	1.83E-06	5.02E-07	5.27	5.47	2.22	hsa-miR-548f-5p
MIMAT0003339	5.73E-06	1.77E-06	4.99	4.25	2.218	hsa-miR-421
MIMAT0018193	1.21E-04	5.13E-05	4.18	1.02	2.217	hsa-miR-3919
MIMAT0003267	4.02E-05	1.52E-05	4.48	2.18	2.216	hsa-miR-599
MIMAT0004600	2.68E-07	6.17E-08	5.72	7.5	2.214	hsa-miR-144-5p
MIMAT0019802	1.59E-06	4.33E-07	5.3	5.61	2.214	hsa-miR-4703-3p
MIMAT0004675	1.76E-05	6.01E-06	4.71	3.07	2.211	hsa-miR-219a-2-3p
MIMAT0019235	3.53E-05	1.31E-05	4.52	2.32	2.211	hsa-miR-4520-5p
MIMAT0022923	1.00E-08	1.71E-09	6.45	11	2.211	hsa-miR-376b-5p
MIMAT0003242	3.34E-05	1.23E-05	4.53	2.38	2.208	hsa-miR-577
MIMAT0017995	4.93E-07	1.20E-07	5.58	6.85	2.206	hsa-miR-3616-5p
MIMAT0004514	8.49E-05	3.46E-05	4.28	1.39	2.205	hsa-miR-29b-1-5p
MIMAT0003235	7.04E-06	2.22E-06	4.94	4.03	2.202	hsa-miR-570-3p
MIMAT0001629	1.18E-04	5.01E-05	4.18	1.04	2.2	hsa-miR-329-3p
MIMAT0000270	8.63E-05	3.54E-05	4.27	1.37	2.199	hsa-miR-181a-3p
MIMAT0019952	2.93E-06	8.49E-07	5.15	4.96	2.198	hsa-miR-2467-5p
MIMAT0019834	1.11E-06	2.92E-07	5.39	5.99	2.197	hsa-miR-4720-3p
MIMAT0019694	1.56E-04	6.79E-05	4.11	0.75	2.194	hsa-miR-4637
MIMAT0018098	6.51E-07	1.62E-07	5.52	6.56	2.193	hsa-miR-3675-5p
MIMAT0021017	3.51E-07	8.19E-08	5.66	7.23	2.192	hsa-miR-4999-5p
MIMAT0019897	1.15E-06	3.03E-07	5.38	5.96	2.192	hsa-miR-499b-5p
MIMAT0019030	1.76E-07	3.87E-08	5.82	7.96	2.192	hsa-miR-4495
MIMAT0024612	4.09E-06	1.22E-06	5.07	4.61	2.191	hsa-miR-378j
MIMAT0000088	3.59E-05	1.33E-05	4.51	2.31	2.191	hsa-miR-30a-3p
MIMAT0018116	4.62E-07	1.11E-07	5.6	6.93	2.191	hsa-miR-3688-3p
MIMAT0000734	2.67E-05	9.62E-06	4.59	2.62	2.188	hsa-miR-380-5p
MIMAT0016880	5.21E-11	4.53E-12	7.57	16.8	2.188	hsa-miR-4259
MIMAT0022481	6.22E-06	1.95E-06	4.97	4.16	2.182	hsa-miR-5689
MIMAT0019002	9.19E-05	3.78E-05	4.26	1.31	2.181	hsa-miR-4475
MIMAT0019222	4.61E-05	1.76E-05	4.45	2.04	2.174	hsa-miR-3682-5p
MIMAT0022478	1.39E-05	4.63E-06	4.77	3.32	2.173	hsa-miR-5687
MIMAT0021122	5.83E-05	2.26E-05	4.39	1.8	2.171	hsa-miR-5191
MIMAT0004975	4.27E-05	1.62E-05	4.47	2.12	2.17	hsa-miR-509-3-5p
MIMAT0004798	7.83E-07	2.01E-07	5.47	6.36	2.169	hsa-miR-548b-5p
MIMAT0018086	1.99E-05	6.93E-06	4.67	2.93	2.169	hsa-miR-3664-5p
MIMAT0003163	2.15E-05	7.53E-06	4.65	2.85	2.166	hsa-miR-539-5p
MIMAT0000438	5.88E-05	2.28E-05	4.38	1.79	2.166	hsa-miR-152-3p
MIMAT0000256	3.25E-05	1.19E-05	4.54	2.41	2.166	hsa-miR-181a-5p
MIMAT0019000	2.27E-05	8.04E-06	4.64	2.79	2.164	hsa-miR-4473
MIMAT0019057	2.27E-06	6.42E-07	5.22	5.23	2.164	hsa-miR-4520-3p
MIMAT0005828	4.62E-07	1.10E-07	5.6	6.94	2.164	hsa-miR-1183
MIMAT0022264	2.32E-05	8.23E-06	4.63	2.77	2.164	hsa-miR-548aq-3p
MIMAT0027643	2.97E-06	8.63E-07	5.15	4.94	2.163	hsa-miR-6871-3p
MIMAT0031000	4.41E-36	8.60E-39	18.2	77.6	2.16	hsa-miR-8073
MIMAT0004813	1.18E-05	3.87E-06	4.81	3.5	2.158	hsa-miR-411-3p
MIMAT0003277	2.47E-05	8.81E-06	4.61	2.7	2.158	hsa-miR-609
MIMAT0003281	8.96E-06	2.87E-06	4.88	3.78	2.156	hsa-miR-613
MIMAT0003886	5.47E-05	2.10E-05	4.4	1.87	2.156	hsa-miR-769-5p
MIMAT0022701	1.18E-04	4.97E-05	4.19	1.05	2.154	hsa-miR-506-5p
MIMAT0004928	1.37E-05	4.55E-06	4.77	3.34	2.152	hsa-miR-147b
MIMAT0005900	6.09E-05	2.38E-05	4.37	1.75	2.152	hsa-miR-1248
MIMAT0004814	1.28E-05	4.21E-06	4.79	3.41	2.151	hsa-miR-654-3p
MIMAT0004185	1.13E-05	3.68E-06	4.82	3.54	2.151	hsa-miR-802
MIMAT0018941	9.07E-06	2.91E-06	4.87	3.77	2.15	hsa-miR-4426
MIMAT0019920	1.01E-05	3.24E-06	4.85	3.67	2.149	hsa-miR-4768-5p
MIMAT0000727	7.41E-05	2.97E-05	4.32	1.54	2.146	hsa-miR-374a-5p
MIMAT0004557	1.08E-04	4.51E-05	4.21	1.14	2.146	hsa-miR-34a-3p
MIMAT0018938	3.64E-06	1.07E-06	5.1	4.73	2.146	hsa-miR-548ac

MIMAT0028229	1.49E-06	4.05E-07	5.32	5.68	2.139	hsa-miR-7159-3p
MIMAT0022255	4.42E-05	1.68E-05	4.46	2.09	2.139	hsa-miR-4524b-5p
MIMAT0000442	3.52E-04	1.65E-04	3.87	-0.09	2.136	hsa-miR-9-3p
MIMAT0019921	5.09E-06	1.54E-06	5.02	4.38	2.135	hsa-miR-4768-3p
MIMAT0022485	5.64E-06	1.74E-06	4.99	4.26	2.133	hsa-miR-4666b
MIMAT0000719	1.60E-04	7.00E-05	4.1	0.72	2.133	hsa-miR-367-3p
MIMAT0019066	1.49E-05	5.03E-06	4.75	3.24	2.13	hsa-miR-4527
MIMAT0000086	5.59E-04	2.71E-04	3.74	-0.56	2.126	hsa-miR-29a-3p
MIMAT0026613	1.83E-05	6.31E-06	4.69	3.02	2.126	hsa-miR-510-3p
MIMAT0002851	8.32E-05	3.39E-05	4.28	1.41	2.123	hsa-miR-517-5p
MIMAT0003293	2.39E-05	8.48E-06	4.62	2.74	2.122	hsa-miR-624-5p
MIMAT0022712	6.67E-08	1.35E-08	6.03	8.98	2.121	hsa-miR-1271-3p
MIMAT0004509	2.73E-05	9.88E-06	4.59	2.59	2.118	hsa-miR-93-3p
MIMAT0005874	4.94E-05	1.89E-05	4.43	1.97	2.117	hsa-miR-548e-3p
MIMAT0019204	3.15E-05	1.15E-05	4.55	2.45	2.117	hsa-miR-3140-5p
MIMAT0028225	1.46E-06	3.95E-07	5.32	5.7	2.116	hsa-miR-7157-3p
MIMAT0031074	3.77E-05	1.41E-05	4.5	2.26	2.114	hsa-miR-450a-2-3p
MIMAT0004701	1.34E-05	4.43E-06	4.78	3.36	2.113	hsa-miR-338-5p
MIMAT0004588	4.66E-06	1.40E-06	5.04	4.47	2.11	hsa-miR-27b-5p
MIMAT0019906	8.10E-05	3.28E-05	4.29	1.45	2.109	hsa-miR-4760-5p
MIMAT0019957	4.42E-35	1.20E-37	17.7	75	2.108	hsa-miR-4787-3p
MIMAT0019911	2.58E-05	9.27E-06	4.6	2.66	2.105	hsa-miR-4762-3p
MIMAT0000703	6.36E-05	2.50E-05	4.36	1.71	2.104	hsa-miR-361-5p
MIMAT0015088	1.18E-06	3.12E-07	5.37	5.93	2.104	hsa-miR-514b-3p
MIMAT0000428	2.32E-04	1.05E-04	3.99	0.33	2.102	hsa-miR-135a-5p
MIMAT0004958	7.30E-05	2.91E-05	4.32	1.56	2.102	hsa-miR-301b-3p
MIMAT0003393	8.28E-05	3.37E-05	4.29	1.42	2.101	hsa-miR-425-5p
MIMAT0018996	1.88E-05	6.51E-06	4.69	2.99	2.101	hsa-miR-4469
MIMAT0021038	1.82E-04	8.09E-05	4.06	0.59	2.1	hsa-miR-548ap-3p
MIMAT0031013	1.13E-05	3.66E-06	4.82	3.55	2.096	hsa-miR-8086
MIMAT0003338	1.99E-05	6.95E-06	4.67	2.93	2.092	hsa-miR-660-5p
MIMAT0004506	1.13E-04	4.76E-05	4.2	1.09	2.09	hsa-miR-33a-3p
MIMAT0021029	3.05E-05	1.11E-05	4.56	2.48	2.089	hsa-miR-548ao-5p
MIMAT0003250	7.34E-05	2.94E-05	4.32	1.55	2.089	hsa-miR-585-3p
MIMAT0019976	3.65E-05	1.36E-05	4.51	2.29	2.088	hsa-miR-4799-5p
MIMAT0028227	1.31E-05	4.33E-06	4.78	3.39	2.088	hsa-miR-7158-3p
MIMAT0004779	4.47E-05	1.70E-05	4.46	2.08	2.088	hsa-miR-509-5p
MIMAT0004801	2.59E-05	9.30E-06	4.6	2.65	2.087	hsa-miR-590-3p
MIMAT0009197	4.04E-06	1.20E-06	5.08	4.62	2.084	hsa-miR-205-3p
MIMAT0015015	8.96E-06	2.86E-06	4.88	3.79	2.083	hsa-miR-3144-3p
MIMAT0000075	8.14E-04	4.05E-04	3.62	-0.94	2.083	hsa-miR-20a-5p
MIMAT0019716	6.83E-05	2.71E-05	4.34	1.63	2.083	hsa-miR-4652-5p
MIMAT0005904	2.18E-06	6.13E-07	5.23	5.27	2.082	hsa-miR-1253
MIMAT0015046	1.13E-05	3.68E-06	4.82	3.54	2.081	hsa-miR-3171
MIMAT0019076	1.13E-05	3.67E-06	4.82	3.54	2.08	hsa-miR-548am-3p
MIMAT0016858	2.24E-06	6.31E-07	5.22	5.25	2.078	hsa-miR-4306
MIMAT0004959	5.52E-05	2.13E-05	4.4	1.86	2.076	hsa-miR-216b-5p
MIMAT0018187	1.47E-05	4.94E-06	4.75	3.26	2.076	hsa-miR-3913-5p
MIMAT0016871	2.51E-05	8.98E-06	4.61	2.69	2.074	hsa-miR-4320
MIMAT0028234	6.50E-05	2.56E-05	4.35	1.68	2.073	hsa-miR-7162-5p
MIMAT0022692	2.83E-05	1.02E-05	4.58	2.56	2.072	hsa-miR-181b-3p
MIMAT0018197	1.92E-05	6.66E-06	4.68	2.97	2.071	hsa-miR-3922-3p
MIMAT0000276	2.55E-05	9.15E-06	4.61	2.67	2.07	hsa-miR-219a-5p
MIMAT0015086	2.45E-06	6.96E-07	5.2	5.15	2.07	hsa-miR-3201
MIMAT0004901	6.51E-05	2.57E-05	4.35	1.68	2.068	hsa-miR-298
MIMAT0030994	3.16E-05	1.16E-05	4.55	2.44	2.068	hsa-miR-8067
MIMAT0016887	3.09E-05	1.13E-05	4.55	2.47	2.067	hsa-miR-4325
MIMAT0019047	1.60E-05	5.42E-06	4.73	3.17	2.067	hsa-miR-4510

MIMAT0003389	2.16E-05	7.60E-06	4.65	2.85	2.066	hsa-miR-542-3p
MIMAT0022280	1.05E-05	3.38E-06	4.84	3.62	2.066	hsa-miR-5582-3p
MIMAT0026620	8.74E-05	3.59E-05	4.27	1.36	2.065	hsa-miR-598-5p
MIMAT0026640	1.91E-05	6.60E-06	4.68	2.98	2.065	hsa-miR-670-3p
MIMAT0005877	3.56E-05	1.32E-05	4.52	2.32	2.064	hsa-miR-1286
MIMAT0022713	5.30E-22	6.61E-24	12.2	43.7	2.057	hsa-miR-1185-2-3p
MIMAT0003879	7.40E-05	2.97E-05	4.32	1.54	2.054	hsa-miR-758-3p
MIMAT0022722, M	2.91E-05	1.06E-05	4.57	2.53	2.053	hsa-miR-548g-5p, hsa-miR-548x-5p, hsa-miR-548aj-5p
MIMAT0000730	5.61E-04	2.72E-04	3.74	-0.56	2.051	hsa-miR-377-3p
MIMAT0000456	3.47E-04	1.62E-04	3.88	-0.07	2.048	hsa-miR-186-5p
MIMAT0018196	2.25E-05	7.97E-06	4.64	2.8	2.048	hsa-miR-3921
MIMAT0030980	8.32E-05	3.39E-05	4.28	1.41	2.046	hsa-miR-8053
MIMAT0005907	2.31E-04	1.05E-04	3.99	0.34	2.045	hsa-miR-1256
MIMAT0019054	1.16E-04	4.90E-05	4.19	1.06	2.045	hsa-miR-4517
MIMAT0021035	1.64E-04	7.19E-05	4.09	0.7	2.042	hsa-miR-5007-5p
MIMAT0019052	6.62E-09	1.09E-09	6.53	11.4	2.042	hsa-miR-4515
MIMAT0004511	1.60E-04	6.98E-05	4.1	0.73	2.04	hsa-miR-99a-3p
MIMAT0018983	2.06E-05	7.22E-06	4.66	2.89	2.036	hsa-miR-4461
MIMAT0025459	7.18E-05	2.87E-05	4.33	1.57	2.036	hsa-miR-6501-3p
MIMAT0000429	1.78E-04	7.89E-05	4.07	0.61	2.035	hsa-miR-137
MIMAT0004927	1.97E-05	6.84E-06	4.67	2.95	2.033	hsa-miR-708-3p
MIMAT0019218	8.19E-05	3.32E-05	4.29	1.43	2.033	hsa-miR-3194-3p
MIMAT0015008	3.18E-06	9.28E-07	5.13	4.87	2.031	hsa-miR-3140-3p
MIMAT0022501	7.35E-05	2.95E-05	4.32	1.55	2.028	hsa-miR-5707
MIMAT0004489	3.55E-04	1.66E-04	3.87	-0.1	2.024	hsa-miR-16-1-3p
MIMAT0004702	3.16E-05	1.16E-05	4.55	2.44	2.023	hsa-miR-339-3p
MIMAT0019211	7.88E-22	1.01E-23	12.2	43.3	2.023	hsa-miR-3158-5p
MIMAT0001621	1.79E-04	7.96E-05	4.07	0.6	2.021	hsa-miR-369-5p
MIMAT0019081	5.47E-05	2.10E-05	4.4	1.87	2.02	hsa-miR-4538
MIMAT0003290	5.90E-05	2.29E-05	4.38	1.79	2.019	hsa-miR-621
MIMAT0005950	1.08E-04	4.51E-05	4.21	1.14	2.017	hsa-miR-1306-3p
MIMAT0004805	1.22E-04	5.21E-05	4.17	1	2.016	hsa-miR-616-3p
MIMAT0005908	1.87E-04	8.35E-05	4.05	0.56	2.016	hsa-miR-1257
MIMAT0015081	9.42E-05	3.89E-05	4.25	1.28	2.015	hsa-miR-548x-3p
MIMAT0026744	2.99E-05	1.08E-05	4.56	2.51	2.014	hsa-miR-1252-3p
MIMAT0025843	1.05E-04	4.37E-05	4.22	1.17	2.007	hsa-miR-6715b-3p
MIMAT0015087	5.23E-08	1.03E-08	6.09	9.25	2.006	hsa-miR-514b-5p
MIMAT0015020	1.91E-05	6.61E-06	4.68	2.98	2.005	hsa-miR-548v
MIMAT0019962	1.30E-04	5.58E-05	4.16	0.94	2.005	hsa-miR-4790-3p
MIMAT0022947	4.40E-27	3.43E-29	14.3	55.8	2.004	hsa-miR-1238-5p
MIMAT0019725	1.46E-05	4.93E-06	4.75	3.26	2.004	hsa-miR-4658
MIMAT0019221	6.01E-05	2.34E-05	4.38	1.77	2.004	hsa-miR-3677-5p
MIMAT0027607	2.90E-04	1.34E-04	3.93	0.11	2.002	hsa-miR-6853-3p
MIMAT0025850	2.66E-06	7.65E-07	5.18	5.06	2.002	hsa-miR-6719-3p
MIMAT0032026	1.33E-04	5.70E-05	4.15	0.92	2.001	hsa-miR-301b-5p

GSE 113486 downregulated miRNAs

ID	adj.P.Val	P.Value	t	B	logFC	miRNA_ID_LIST
MIMAT0004602	4.41E-21	7.22E-23	-12	41.4	-3.14	hsa-miR-125a-3p
MIMAT0004592	8.57E-10	1.11E-10	-7	13.7	-3.13	hsa-miR-125b-1-3p
MIMAT0015017	3.38E-13	1.74E-14	-8.6	22.3	-2.69	hsa-miR-1273c
MIMAT0025458	1.05E-09	1.39E-10	-6.9	13.5	-2.63	hsa-miR-6501-5p
MIMAT0019710	3.74E-20	7.29E-22	-11	39.1	-2.58	hsa-miR-4648
MIMAT0026481	5.11E-14	2.09E-15	-8.9	24.4	-2.5	hsa-miR-134-3p
MIMAT0019015	4.32E-09	6.84E-10	-6.6	11.9	-2.44	hsa-miR-4481
MIMAT0019852	2.28E-38	3.56E-41	-19	83.1	-2.43	hsa-miR-4730

MIMAT0027555	3.97E-11	3.38E-12	-7.6	17.1	-2.35	hsa-miR-6827-3p
MIMAT0005866	5.1E-21	8.76E-23	-12	41.2	-2.33	hsa-miR-1203
MIMAT0003240	1.49E-21	2.05E-23	-12	42.6	-2.31	hsa-miR-575
MIMAT0018164	6.94E-07	1.76E-07	-5.5	6.48	-2.29	hsa-miR-3713
MIMAT0027614	1.22E-14	4.23E-16	-9.2	26	-2.27	hsa-miR-6857-5p
MIMAT0021033	3.01E-10	3.36E-11	-7.2	14.9	-2.19	hsa-miR-5006-5p
MIMAT0027363	6.21E-09	1.02E-09	-6.5	11.5	-2.11	hsa-miR-6731-5p
MIMAT0026554	0.0000396	0.0000149	-4.5	2.2	-2.03	hsa-miR-433-5p

GSE 106817 upregulated miRNAs

ID	adj.P.Val	P.Value	t	B	logFC	miRNA_ID_LIST
MIMAT0000461	1.03E-119	1.28E-121	24.6	267	6.216	hsa-miR-195-5p
MIMAT0005898	3.05E-61	1.47E-62	17.1	131	5.82	hsa-miR-1246
MIMAT0000069	3.33E-81	9.48E-83	19.9	178	5.579	hsa-miR-16-5p
MIMAT0000278	1.54E-94	2.76E-96	21.6	209	5.269	hsa-miR-221-3p
MIMAT0005880	9.95E-65	4.29E-66	17.6	139	5.26	hsa-miR-1290
MIMAT0000440	1.14E-61	5.43E-63	17.2	132	5.129	hsa-miR-191-5p
MIMAT0000062	1.36E-66	5.24E-68	17.9	144	4.966	hsa-let-7a-5p
MIMAT0015072	4.42E-71	1.58E-72	18.5	154	4.907	hsa-miR-320e
MIMAT0019025	1.67E-66	6.52E-68	17.9	144	4.651	hsa-miR-4490
MIMAT0004597	1.91E-66	7.53E-68	17.9	143	4.646	hsa-miR-140-3p
MIMAT0004514	1.57E-55	9.70E-57	16.2	118	4.479	hsa-miR-29b-1-5p
MIMAT0001631	1.38E-44	1.47E-45	14.4	92.4	4.427	hsa-miR-451a
MIMAT0004697	6.97E-71	2.55E-72	18.5	154	4.334	hsa-miR-151a-5p
MIMAT0000100	7.35E-53	5.13E-54	15.8	112	4.321	hsa-miR-29b-3p
MIMAT0006764	1.17E-55	7.16E-57	16.2	118	4.276	hsa-miR-320d
MIMAT0022497	1.52E-56	9.13E-58	16.4	120	4.225	hsa-miR-5692b
MIMAT0019901	3.57E-45	3.67E-46	14.5	93.8	4.216	hsa-miR-4757-5p
MIMAT0023698	2.99E-41	3.70E-42	13.8	84.6	4.165	hsa-miR-6073
MIMAT0003252	1.81E-51	1.33E-52	15.6	109	4.158	hsa-miR-586
MIMAT0019223	1.50E-57	8.71E-59	16.5	123	4.132	hsa-miR-3688-5p
MIMAT0019935	4.79E-58	2.69E-59	16.6	124	4.114	hsa-miR-4777-3p
MIMAT0000097	2.37E-45	2.41E-46	14.6	94.2	4.114	hsa-miR-99a-5p
MIMAT0000065	6.06E-47	5.65E-48	14.8	97.9	4.099	hsa-let-7d-5p
MIMAT0000425	9.05E-47	8.50E-48	14.8	97.5	4.094	hsa-miR-130a-3p
MIMAT0010214	1.22E-43	1.37E-44	14.3	90.2	4.09	hsa-miR-151b
MIMAT0000101	1.27E-45	1.27E-46	14.6	94.8	4.082	hsa-miR-103a-3p
MIMAT0000096	6.21E-50	4.89E-51	15.3	105	4.071	hsa-miR-98-5p
MIMAT0000071	6.79E-60	3.55E-61	16.9	128	4.035	hsa-miR-17-3p
MIMAT0003254	4.24E-49	3.58E-50	15.2	103	4.033	hsa-miR-548b-3p
MIMAT0002864	1.21E-41	1.46E-42	13.9	85.5	3.967	hsa-miR-518d-3p
MIMAT0000064	4.07E-45	4.20E-46	14.5	93.6	3.916	hsa-let-7c-5p
MIMAT0000067	8.65E-50	6.88E-51	15.3	105	3.888	hsa-let-7f-5p
MIMAT0000082	6.47E-40	8.55E-41	13.6	81.5	3.885	hsa-miR-26a-5p
MIMAT0001413	3.28E-44	3.57E-45	14.4	91.5	3.868	hsa-miR-20b-5p
MIMAT0003255	2.90E-59	1.56E-60	16.8	127	3.849	hsa-miR-588
MIMAT0003253	1.37E-57	7.92E-59	16.5	123	3.845	hsa-miR-587
MIMAT0000083	4.48E-45	4.64E-46	14.5	93.5	3.831	hsa-miR-26b-5p
MIMAT0002869	2.14E-51	1.59E-52	15.6	108	3.829	hsa-miR-519a-3p
MIMAT0004809	7.42E-66	3.01E-67	17.8	142	3.817	hsa-miR-628-5p
MIMAT0019741	8.12E-47	7.60E-48	14.8	97.6	3.804	hsa-miR-4666a-5p
MIMAT0019014	2.73E-35	4.55E-36	12.7	70.7	3.8	hsa-miR-4480
MIMAT0004505	5.95E-48	5.36E-49	15	100	3.748	hsa-miR-32-3p
MIMAT0024615	1.56E-89	3.70E-91	21	197	3.736	hsa-miR-6131
MIMAT0000104	1.93E-38	2.80E-39	13.3	78	3.726	hsa-miR-107
MIMAT0015018	2.93E-49	2.43E-50	15.2	103	3.676	hsa-miR-3146
MIMAT0004700	8.87E-57	5.29E-58	16.4	121	3.673	hsa-miR-331-5p
MIMAT0002888	3.90E-55	2.46E-56	16.2	117	3.637	hsa-miR-532-5p

MIMAT0016844	4.18E-41	5.20E-42	13.8	84.3	3.631	hsa-miR-4295
MIMAT0002866	7.01E-50	5.55E-51	15.3	105	3.626	hsa-miR-517c-3p
MIMAT0000079	6.05E-35	1.03E-35	12.6	69.9	3.614	hsa-miR-24-1-5p
MIMAT0000099	8.89E-42	1.07E-42	13.9	85.8	3.606	hsa-miR-101-3p
MIMAT0004594	5.24E-62	2.45E-63	17.2	133	3.6	hsa-miR-132-5p
MIMAT0005893	9.70E-45	1.02E-45	14.4	92.7	3.588	hsa-miR-1305
MIMAT0022259	1.92E-202	1.50E-205	33.3	460	3.576	hsa-miR-5100
MIMAT0003220	7.88E-64	3.53E-65	17.5	137	3.564	hsa-miR-556-5p
MIMAT0000252	2.41E-45	2.46E-46	14.6	94.2	3.557	hsa-miR-7-5p
MIMAT0000436	2.16E-41	2.65E-42	13.9	84.9	3.552	hsa-miR-144-3p
MIMAT0004681	3.83E-39	5.27E-40	13.4	79.7	3.548	hsa-miR-26a-2-3p
MIMAT0035704	1.15E-63	5.24E-65	17.5	137	3.543	hsa-miR-548bb-3p
MIMAT0000077	9.79E-44	1.09E-44	14.3	90.4	3.541	hsa-miR-22-3p
MIMAT0002883	3.62E-61	1.79E-62	17.1	131	3.537	hsa-miR-514a-3p
MIMAT0003329	2.98E-35	5.01E-36	12.7	70.6	3.53	hsa-miR-411-5p
MIMAT0027627	4.99E-40	6.55E-41	13.6	81.7	3.517	hsa-miR-6863
MIMAT0002882	5.56E-48	4.99E-49	15	100	3.512	hsa-miR-510-5p
MIMAT0000074	2.97E-32	5.90E-33	12.1	63.6	3.5	hsa-miR-19b-3p
MIMAT0019230	7.56E-49	6.49E-50	15.1	102	3.496	hsa-miR-3942-3p
MIMAT0003238	2.71E-44	2.91E-45	14.4	91.7	3.492	hsa-miR-573
MIMAT0000418	3.12E-61	1.52E-62	17.1	131	3.485	hsa-miR-23b-3p
MIMAT0016885	2.78E-55	1.73E-56	16.2	117	3.458	hsa-miR-4255
MIMAT0000098	5.11E-36	8.29E-37	12.9	72.4	3.455	hsa-miR-100-5p
MIMAT0019727	3.07E-44	3.33E-45	14.4	91.6	3.447	hsa-miR-4659a-3p
MIMAT0000419	2.04E-38	2.97E-39	13.3	78	3.446	hsa-miR-27b-3p
MIMAT0019875	9.90E-40	1.32E-40	13.6	81	3.436	hsa-miR-4744
MIMAT0019232	4.20E-34	7.55E-35	12.5	67.9	3.432	hsa-miR-4423-5p
MIMAT0000070	1.72E-31	3.52E-32	12	61.8	3.414	hsa-miR-17-5p
MIMAT0000279	6.47E-34	1.18E-34	12.4	67.4	3.411	hsa-miR-222-3p
MIMAT0004698	1.56E-60	7.92E-62	17	130	3.408	hsa-miR-135b-3p
MIMAT0000274	1.78E-44	1.91E-45	14.4	92.1	3.404	hsa-miR-217
MIMAT0000068	1.89E-38	2.73E-39	13.3	78	3.404	hsa-miR-15a-5p
MIMAT0002868	4.13E-48	3.65E-49	15	101	3.395	hsa-miR-522-3p
MIMAT0002873	5.64E-38	8.44E-39	13.2	76.9	3.395	hsa-miR-502-5p
MIMAT0000421	4.68E-34	8.43E-35	12.5	67.8	3.388	hsa-miR-122-5p
MIMAT0000072	9.09E-35	1.57E-35	12.6	69.4	3.378	hsa-miR-18a-5p
MIMAT0022500	8.66E-52	6.28E-53	15.6	109	3.368	hsa-miR-5706
MIMAT0019925	1.98E-31	4.06E-32	11.9	61.6	3.354	hsa-miR-4771
MIMAT0000066	1.20E-38	1.71E-39	13.4	78.5	3.343	hsa-let-7e-5p
MIMAT0003258	4.36E-49	3.70E-50	15.2	103	3.337	hsa-miR-590-5p
MIMAT0000281	5.77E-34	1.05E-34	12.5	67.6	3.337	hsa-miR-224-5p
MIMAT0019969	2.36E-28	5.81E-29	11.3	54.4	3.321	hsa-miR-4795-3p
MIMAT0000434	3.01E-36	4.82E-37	12.9	72.9	3.313	hsa-miR-142-3p
MIMAT0022265	5.40E-33	1.04E-33	12.3	65.3	3.312	hsa-miR-548ar-5p
MIMAT0018093	8.59E-52	6.20E-53	15.6	109	3.308	hsa-miR-3670
MIMAT0003287	3.43E-55	2.15E-56	16.2	117	3.307	hsa-miR-618
MIMAT0002848	2.99E-30	6.67E-31	11.7	58.9	3.298	hsa-miR-518c-3p
MIMAT0004507	6.59E-39	9.30E-40	13.4	79.1	3.296	hsa-miR-92a-1-5p
MIMAT0003331	2.32E-31	4.85E-32	11.9	61.5	3.295	hsa-miR-655-3p
MIMAT0031893	1.85E-36	2.95E-37	12.9	73.4	3.289	hsa-miR-181b-2-3p
MIMAT0017997	1.74E-33	3.23E-34	12.4	66.4	3.288	hsa-miR-3617-5p
MIMAT0019236	3.98E-34	7.13E-35	12.5	67.9	3.271	hsa-miR-4529-5p
MIMAT0002850	1.15E-33	2.10E-34	12.4	66.9	3.27	hsa-miR-524-3p
MIMAT0022288	4.96E-39	6.86E-40	13.4	79.4	3.269	hsa-miR-5586-3p
MIMAT0011157	3.45E-37	5.29E-38	13.1	75.1	3.261	hsa-miR-2114-3p
MIMAT0003286	3.82E-48	3.37E-49	15	101	3.259	hsa-miR-617
MIMAT0019873	1.25E-38	1.78E-39	13.3	78.5	3.256	hsa-miR-4742-3p
MIMAT0004688	3.67E-38	5.46E-39	13.3	77.3	3.253	hsa-miR-374a-3p

MIMAT0019929	4.16E-48	3.69E-49	15	101	3.247	hsa-miR-4774-5p
MIMAT0003219	1.19E-48	1.04E-49	15.1	102	3.241	hsa-miR-555
MIMAT0004699	5.30E-49	4.53E-50	15.2	103	3.237	hsa-miR-148b-5p
MIMAT0003260	3.29E-38	4.85E-39	13.3	77.5	3.236	hsa-miR-592
MIMAT0019746	5.99E-36	9.76E-37	12.8	72.2	3.236	hsa-miR-4668-3p
MIMAT0026623	2.75E-49	2.26E-50	15.2	103	3.23	hsa-miR-627-3p
MIMAT0003288	1.18E-52	8.30E-54	15.8	111	3.229	hsa-miR-619-3p
MIMAT0004768	5.71E-53	3.96E-54	15.8	112	3.228	hsa-miR-497-3p
MIMAT0004913	2.68E-50	2.05E-51	15.4	106	3.222	hsa-miR-891b
MIMAT0003333	7.65E-54	5.13E-55	16	114	3.219	hsa-miR-549a
MIMAT0026606	5.44E-40	7.16E-41	13.6	81.7	3.212	hsa-miR-511-3p
MIMAT0019785	1.18E-54	7.61E-56	16.1	116	3.21	hsa-miR-4693-3p
MIMAT0002849	6.00E-33	1.16E-33	12.2	65.2	3.2	hsa-miR-524-5p
MIMAT0002870	1.66E-50	1.25E-51	15.4	106	3.198	hsa-miR-499a-5p
MIMAT0000433	1.49E-31	3.03E-32	12	61.9	3.198	hsa-miR-142-5p
MIMAT0002876	1.09E-35	1.79E-36	12.8	71.6	3.191	hsa-miR-505-3p
MIMAT0000688	1.92E-38	2.78E-39	13.3	78	3.178	hsa-miR-301a-3p
MIMAT0018982	8.06E-26	2.28E-26	10.7	48.5	3.177	hsa-miR-4460
MIMAT0019751	5.18E-39	7.21E-40	13.4	79.4	3.167	hsa-miR-4670-3p
MIMAT0018095	5.80E-64	2.56E-65	17.5	138	3.158	hsa-miR-3672
MIMAT0018112	8.33E-64	3.77E-65	17.5	137	3.153	hsa-miR-3684
MIMAT0018094	3.41E-61	1.67E-62	17.1	131	3.152	hsa-miR-3671
MIMAT0019805	1.12E-53	7.63E-55	15.9	114	3.151	hsa-miR-4705
MIMAT0019972	3.50E-30	7.92E-31	11.7	58.7	3.15	hsa-miR-4797-5p
MIMAT0015060	5.61E-41	7.07E-42	13.8	84	3.149	hsa-miR-548w
MIMAT0027581	5.33E-41	6.69E-42	13.8	84	3.144	hsa-miR-6839-3p
MIMAT0019831	5.29E-47	4.91E-48	14.8	98	3.143	hsa-miR-4718
MIMAT0000435	4.77E-32	9.58E-33	12.1	63.1	3.138	hsa-miR-143-3p
MIMAT0018078	6.82E-59	3.75E-60	16.7	126	3.137	hsa-miR-3658
MIMAT0019203	8.49E-66	3.48E-67	17.8	142	3.135	hsa-miR-3136-3p
MIMAT0019959	1.64E-26	4.54E-27	10.9	50.1	3.134	hsa-miR-4789-5p
MIMAT0003279	8.48E-33	1.65E-33	12.2	64.8	3.131	hsa-miR-611
MIMAT0015056	4.97E-62	2.30E-63	17.2	133	3.127	hsa-miR-3179
MIMAT0004683	1.30E-35	2.16E-36	12.8	71.4	3.126	hsa-miR-362-3p
MIMAT0019816	3.73E-56	2.27E-57	16.3	119	3.123	hsa-miR-4711-5p
MIMAT0004513	3.68E-32	7.37E-33	12.1	63.3	3.12	hsa-miR-101-5p
MIMAT0003251	1.39E-28	3.39E-29	11.3	55	3.115	hsa-miR-548a-3p
MIMAT0015012	1.57E-54	1.01E-55	16.1	116	3.11	hsa-miR-3143
MIMAT0019730	7.09E-38	1.06E-38	13.2	76.7	3.107	hsa-miR-4661-3p
MIMAT0000449	3.37E-28	8.42E-29	11.3	54.1	3.102	hsa-miR-146a-5p
MIMAT0000090	3.28E-28	8.18E-29	11.3	54.1	3.095	hsa-miR-32-5p
MIMAT0000646	1.49E-31	3.02E-32	12	61.9	3.093	hsa-miR-155-5p
MIMAT0019924	8.11E-46	8.09E-47	14.6	95.3	3.093	hsa-miR-4770
MIMAT0027629	4.41E-31	9.30E-32	11.9	60.8	3.091	hsa-miR-6864-3p
MIMAT0019218	3.53E-33	6.67E-34	12.3	65.7	3.083	hsa-miR-3194-3p
MIMAT0019690	3.41E-22	1.13E-22	9.88	40.1	3.082	hsa-miR-4633-3p
MIMAT0019748	3.44E-39	4.70E-40	13.5	79.8	3.082	hsa-miR-219b-3p
MIMAT0027579	4.68E-44	5.12E-45	14.3	91.1	3.079	hsa-miR-6838-3p
MIMAT0000089	4.69E-27	1.26E-27	11	51.4	3.076	hsa-miR-31-5p
MIMAT0002867	1.35E-33	2.49E-34	12.4	66.7	3.073	hsa-miR-520h
MIMAT0022484	3.70E-34	6.62E-35	12.5	68	3.073	hsa-miR-5692a
MIMAT0003285	6.84E-40	9.07E-41	13.6	81.4	3.07	hsa-miR-548c-3p
MIMAT0002172	1.95E-34	3.44E-35	12.5	68.7	3.068	hsa-miR-376b-3p
MIMAT0016902	4.92E-42	5.86E-43	14	86.4	3.068	hsa-miR-4272
MIMAT0022965	1.18E-46	1.13E-47	14.8	97.2	3.068	hsa-miR-3606-3p
MIMAT0000075	1.81E-27	4.77E-28	11.1	52.3	3.066	hsa-miR-20a-5p
MIMAT0004549	6.08E-44	6.71E-45	14.3	90.9	3.063	hsa-miR-148a-5p
MIMAT0019763	1.09E-42	1.27E-43	14.1	87.9	3.06	hsa-miR-4679

MIMAT0019078	2.96E-51	2.21E-52	15.5	108	3.051	hsa-miR-4536-5p
MIMAT0003321	4.83E-37	7.44E-38	13	74.8	3.047	hsa-miR-651-5p
MIMAT0004917	1.27E-47	1.15E-48	14.9	99.5	3.044	hsa-miR-888-3p
MIMAT0000691	4.30E-28	1.08E-28	11.2	53.8	3.043	hsa-miR-130b-3p
MIMAT0019742	2.31E-35	3.85E-36	12.7	70.8	3.042	hsa-miR-4666a-3p
MIMAT0026610	4.00E-36	6.46E-37	12.9	72.6	3.037	hsa-miR-519d-5p
MIMAT0004685	3.70E-38	5.50E-39	13.3	77.3	3.037	hsa-miR-302d-5p
MIMAT0027677	1.04E-46	9.81E-48	14.8	97.4	3.035	hsa-miR-6888-3p
MIMAT0004567	4.37E-48	3.90E-49	15	101	3.032	hsa-miR-219a-1-3p
MIMAT0018950	1.03E-22	3.30E-23	10	41.3	3.03	hsa-miR-4434
MIMAT0014998	1.38E-60	6.89E-62	17	130	3.029	hsa-miR-3133
MIMAT0000063	1.84E-30	4.01E-31	11.7	59.4	3.028	hsa-let-7b-5p
MIMAT0027589	3.40E-38	5.03E-39	13.3	77.4	3.027	hsa-miR-6844
MIMAT0022285	5.48E-46	5.40E-47	14.7	95.7	3.025	hsa-miR-5585-5p
MIMAT0003885	1.41E-51	1.03E-52	15.6	109	3.023	hsa-miR-454-3p
MIMAT0005879	3.62E-33	6.86E-34	12.3	65.7	3.018	hsa-miR-1289
MIMAT0014983	1.04E-46	9.88E-48	14.8	97.4	3.018	hsa-miR-3121-3p
MIMAT0001532	6.21E-28	1.58E-28	11.2	53.4	3.018	hsa-miR-448
MIMAT0004749	3.11E-30	6.96E-31	11.7	58.8	3.015	hsa-miR-424-3p
MIMAT0016898	2.96E-34	5.25E-35	12.5	68.2	3.013	hsa-miR-4263
MIMAT0004490	4.18E-27	1.12E-27	11	51.5	3.008	hsa-miR-19a-5p
MIMAT0019714	2.90E-27	7.75E-28	11.1	51.9	3.007	hsa-miR-4650-3p
MIMAT0001536	4.49E-30	1.02E-30	11.7	58.4	3.006	hsa-miR-429
MIMAT0005793	2.64E-55	1.63E-56	16.2	117	3.004	hsa-miR-320c
MIMAT0003269	9.54E-38	1.44E-38	13.2	76.4	3.001	hsa-miR-601
MIMAT0000682	1.76E-30	3.82E-31	11.7	59.4	3.001	hsa-miR-200a-3p
MIMAT0003317	5.23E-29	1.24E-29	11.4	56	2.997	hsa-miR-647
MIMAT0018195	2.59E-28	6.40E-29	11.3	54.3	2.988	hsa-miR-3920
MIMAT0019036	3.65E-39	5.01E-40	13.4	79.7	2.988	hsa-miR-4500
MIMAT0002831, M	2.71E-30	6.04E-31	11.7	59	2.985	hsa-miR-519c-5p, hsa-miR-523-5p, hsa-miR-518e-5p, hsa-miR-522-5p, hsa-miR-519a-5p, hsa-miR-519b-5p
MIMAT0015004	1.90E-39	2.57E-40	13.5	80.4	2.979	hsa-miR-544b
MIMAT0000084	3.04E-27	8.14E-28	11	51.8	2.971	hsa-miR-27a-3p
MIMAT0000734	2.70E-30	6.01E-31	11.7	59	2.97	hsa-miR-380-5p
MIMAT0000085	5.72E-29	1.36E-29	11.4	55.9	2.968	hsa-miR-28-5p
MIMAT0000733	2.16E-31	4.46E-32	11.9	61.5	2.966	hsa-miR-379-5p
MIMAT0019896	3.42E-22	1.13E-22	9.88	40.1	2.965	hsa-miR-4755-3p
MIMAT0018354	2.08E-59	1.11E-60	16.8	127	2.963	hsa-miR-548y
MIMAT0015040	1.82E-33	3.38E-34	12.4	66.4	2.959	hsa-miR-3166
MIMAT0004812	3.08E-36	4.94E-37	12.9	72.9	2.959	hsa-miR-548d-5p
MIMAT0000445	7.27E-27	1.99E-27	11	50.9	2.954	hsa-miR-126-3p
MIMAT0016872	6.83E-31	1.46E-31	11.8	60.4	2.952	hsa-miR-4317
MIMAT0003216	1.41E-36	2.22E-37	13	73.7	2.952	hsa-miR-553
MIMAT0000254	2.78E-29	6.53E-30	11.5	56.6	2.949	hsa-miR-10b-5p
MIMAT0002171	4.58E-29	1.09E-29	11.4	56.1	2.948	hsa-miR-410-3p
MIMAT0010497	4.83E-33	9.21E-34	12.3	65.4	2.946	hsa-miR-759
MIMAT0018111	3.95E-59	2.14E-60	16.8	126	2.944	hsa-miR-3683
MIMAT0018182	2.06E-54	1.35E-55	16.1	115	2.94	hsa-miR-3908
MIMAT0017985	3.29E-49	2.75E-50	15.2	103	2.935	hsa-miR-3607-3p
MIMAT0018183	1.88E-31	3.85E-32	11.9	61.7	2.934	hsa-miR-3909
MIMAT0000705	2.20E-23	6.90E-24	10.2	42.9	2.934	hsa-miR-362-5p
MIMAT0004801	1.92E-33	3.59E-34	12.3	66.3	2.932	hsa-miR-590-3p
MIMAT0000681	4.86E-26	1.36E-26	10.8	49	2.932	hsa-miR-29c-3p
MIMAT0019199	7.92E-29	1.90E-29	11.4	55.5	2.931	hsa-miR-3121-5p
MIMAT0005894	2.71E-49	2.22E-50	15.2	103	2.922	hsa-miR-1243

MIMAT0005928	2.59E-42	3.02E-43	14	87.1	2.913	hsa-miR-548h-5p
MIMAT0005897	7.54E-31	1.61E-31	11.8	60.3	2.911	hsa-miR-1245a
MIMAT0019004	4.57E-39	6.30E-40	13.4	79.5	2.909	hsa-miR-4477a
MIMAT0000759	1.36E-22	4.37E-23	9.98	41	2.902	hsa-miR-148b-3p
MIMAT0004510	6.32E-32	1.27E-32	12	62.8	2.899	hsa-miR-96-3p
MIMAT0019731	4.98E-41	6.21E-42	13.8	84.1	2.898	hsa-miR-4662a-5p
MIMAT0019916	5.90E-33	1.14E-33	12.3	65.2	2.898	hsa-miR-4765
MIMAT0015007	1.52E-28	3.72E-29	11.3	54.9	2.89	hsa-miR-3139
MIMAT0018083	5.96E-39	8.36E-40	13.4	79.2	2.887	hsa-miR-3662
MIMAT0018941	1.02E-36	1.58E-37	13	74	2.883	hsa-miR-4426
MIMAT0000422	1.16E-27	3.03E-28	11.1	52.8	2.882	hsa-miR-124-3p
MIMAT0026638	3.35E-38	4.95E-39	13.3	77.4	2.881	hsa-miR-1468-3p
MIMAT0005792	6.10E-83	1.67E-84	20.1	182	2.88	hsa-miR-320b
MIMAT0000736	1.37E-29	3.19E-30	11.6	57.3	2.878	hsa-miR-381-3p
MIMAT0002810	9.58E-25	2.83E-25	10.5	46	2.874	hsa-miR-202-5p
MIMAT0004515	1.29E-27	3.39E-28	11.1	52.7	2.874	hsa-miR-29b-2-5p
MIMAT0004613	5.14E-27	1.39E-27	11	51.3	2.871	hsa-miR-188-3p
MIMAT0003163	5.41E-29	1.29E-29	11.4	55.9	2.871	hsa-miR-539-5p
MIMAT0004810	3.10E-22	1.02E-22	9.89	40.2	2.87	hsa-miR-629-5p
MIMAT0003303	8.61E-31	1.85E-31	11.8	60.1	2.868	hsa-miR-633
MIMAT0003235	3.96E-31	8.33E-32	11.9	60.9	2.858	hsa-miR-570-3p
MIMAT0007399	5.81E-28	1.47E-28	11.2	53.5	2.855	hsa-miR-1537-3p
MIMAT0019029	1.12E-35	1.85E-36	12.8	71.6	2.852	hsa-miR-4494
MIMAT0018979	5.92E-36	9.63E-37	12.8	72.2	2.851	hsa-miR-4457
MIMAT0002813	3.05E-24	9.20E-25	10.4	44.9	2.85	hsa-miR-493-5p
MIMAT0011159	4.44E-33	8.45E-34	12.3	65.5	2.847	hsa-miR-2115-3p
MIMAT0000437	5.46E-27	1.49E-27	11	51.2	2.847	hsa-miR-145-5p
MIMAT0004803	3.05E-33	5.74E-34	12.3	65.9	2.846	hsa-miR-548a-5p
MIMAT0000243	1.50E-33	2.76E-34	12.4	66.6	2.844	hsa-miR-148a-3p
MIMAT0000683	1.11E-28	2.70E-29	11.4	55.2	2.843	hsa-miR-302a-5p
MIMAT0022301	3.29E-49	2.76E-50	15.2	103	2.842	hsa-miR-5591-5p
MIMAT0018091	2.79E-44	3.01E-45	14.4	91.7	2.841	hsa-miR-3668
MIMAT0000094	1.09E-24	3.25E-25	10.5	45.9	2.836	hsa-miR-95-3p
MIMAT0018355	1.14E-49	9.14E-51	15.3	104	2.835	hsa-miR-3939
MIMAT0026621	2.49E-43	2.84E-44	14.2	89.4	2.833	hsa-miR-605-3p
MIMAT0027564	3.61E-32	7.21E-33	12.1	63.4	2.831	hsa-miR-6832-5p
MIMAT0022483	5.31E-39	7.41E-40	13.4	79.3	2.827	hsa-miR-5691
MIMAT0000416	4.34E-28	1.09E-28	11.2	53.8	2.826	hsa-miR-1-3p
MIMAT0019782	1.08E-34	1.87E-35	12.6	69.3	2.826	hsa-miR-4691-3p
MIMAT0000414	1.05E-24	3.10E-25	10.5	45.9	2.82	hsa-let-7g-5p
MIMAT0001636	1.59E-21	5.43E-22	9.72	38.5	2.82	hsa-miR-452-3p
MIMAT0026722	1.04E-49	8.27E-51	15.3	104	2.819	hsa-miR-208b-5p
MIMAT0022728	5.57E-57	3.28E-58	16.5	121	2.818	hsa-miR-513c-3p
MIMAT0000715	7.40E-28	1.90E-28	11.2	53.3	2.817	hsa-miR-302b-3p
MIMAT0004511	5.26E-27	1.43E-27	11	51.3	2.815	hsa-miR-99a-3p
MIMAT0026609	1.77E-28	4.35E-29	11.3	54.7	2.813	hsa-miR-520f-5p
MIMAT0022705	2.31E-50	1.77E-51	15.4	106	2.812	hsa-miR-539-3p
MIMAT0004491	7.47E-29	1.79E-29	11.4	55.6	2.81	hsa-miR-19b-1-5p
MIMAT0019918	2.68E-41	3.29E-42	13.8	84.7	2.808	hsa-miR-4766-3p
MIMAT0019832	1.13E-36	1.77E-37	13	73.9	2.807	hsa-miR-4719
MIMAT0019762	3.30E-57	1.93E-58	16.5	122	2.803	hsa-miR-4678
MIMAT0000718	9.97E-32	2.01E-32	12	62.3	2.803	hsa-miR-302d-3p
MIMAT0004909	1.44E-25	4.11E-26	10.7	47.9	2.8	hsa-miR-450b-5p
MIMAT0004784	1.20E-27	3.15E-28	11.1	52.8	2.797	hsa-miR-455-3p
MIMAT0022706	8.22E-52	5.90E-53	15.6	109	2.796	hsa-miR-561-5p
MIMAT0002863	5.13E-22	1.72E-22	9.84	39.7	2.793	hsa-miR-518a-3p
MIMAT0004793	2.62E-27	6.98E-28	11.1	52	2.792	hsa-miR-556-3p
MIMAT0000438	2.03E-27	5.38E-28	11.1	52.2	2.791	hsa-miR-152-3p

MIMAT0022287	1.57E-36	2.49E-37	13	73.6	2.79	hsa-miR-5586-5p
MIMAT0000444	2.85E-24	8.59E-25	10.4	44.9	2.79	hsa-miR-126-5p
MIMAT0005948	6.98E-46	6.94E-47	14.6	95.4	2.789	hsa-miR-664a-5p
MIMAT0000272	3.04E-28	7.56E-29	11.3	54.2	2.789	hsa-miR-215-5p
MIMAT0004972	2.36E-30	5.21E-31	11.7	59.1	2.789	hsa-miR-922
MIMAT0019833	4.70E-44	5.17E-45	14.3	91.1	2.788	hsa-miR-4720-5p
MIMAT0022303	1.02E-48	8.88E-50	15.1	102	2.787	hsa-miR-548av-5p
MIMAT0018198	1.23E-58	6.86E-60	16.7	125	2.786	hsa-miR-3923
MIMAT0003322	9.94E-31	2.14E-31	11.8	60	2.779	hsa-miR-652-3p
MIMAT0005931	7.21E-43	8.32E-44	14.1	88.4	2.774	hsa-miR-302e
MIMAT0019024	9.72E-35	1.68E-35	12.6	69.4	2.773	hsa-miR-548al
MIMAT0019784	2.50E-39	3.39E-40	13.5	80.1	2.771	hsa-miR-4693-5p
MIMAT0000719	1.81E-25	5.22E-26	10.7	47.7	2.768	hsa-miR-367-3p
MIMAT0019040	1.01E-36	1.57E-37	13	74	2.765	hsa-miR-4504
MIMAT0003250	4.26E-24	1.29E-24	10.3	44.5	2.765	hsa-miR-585-3p
MIMAT0002879	1.18E-26	3.26E-27	10.9	50.4	2.76	hsa-miR-507
MIMAT0004566	4.39E-33	8.34E-34	12.3	65.5	2.758	hsa-miR-218-2-3p
MIMAT0004806, M	2.31E-31	4.80E-32	11.9	61.5	2.752	hsa-miR-548c-5p, hsa-miR-548o-5p, hsa-miR-548am-5p
MIMAT0019735	2.23E-25	6.44E-26	10.6	47.5	2.752	hsa-miR-4663
MIMAT0000088	5.33E-27	1.45E-27	11	51.2	2.747	hsa-miR-30a-3p
MIMAT0000717	2.29E-28	5.64E-29	11.3	54.5	2.747	hsa-miR-302c-3p
MIMAT0000091	9.49E-24	2.92E-24	10.3	43.7	2.745	hsa-miR-33a-5p
MIMAT0020957	3.33E-22	1.10E-22	9.89	40.1	2.741	hsa-miR-548ah-3p
MIMAT0022267	3.60E-22	1.19E-22	9.88	40	2.741	hsa-miR-548as-5p
MIMAT0004551	3.17E-36	5.10E-37	12.9	72.8	2.739	hsa-miR-30d-3p
MIMAT0019938	2.31E-38	3.38E-39	13.3	77.8	2.738	hsa-miR-4779
MIMAT0004692	3.37E-35	5.70E-36	12.7	70.4	2.736	hsa-miR-340-5p
MIMAT0003319	1.23E-30	2.65E-31	11.8	59.8	2.733	hsa-miR-649
MIMAT0019362	4.76E-26	1.33E-26	10.8	49	2.732	hsa-miR-3977
MIMAT0004702	1.52E-30	3.30E-31	11.8	59.6	2.731	hsa-miR-339-3p
MIMAT0003318	2.62E-27	6.97E-28	11.1	52	2.729	hsa-miR-648
MIMAT0019745	7.34E-38	1.10E-38	13.2	76.6	2.728	hsa-miR-4668-5p
MIMAT0019953	6.58E-55	4.21E-56	16.1	117	2.727	hsa-miR-2467-3p
MIMAT0004517	7.74E-20	2.90E-20	9.29	34.6	2.723	hsa-miR-106a-3p
MIMAT0025459	5.28E-27	1.43E-27	11	51.3	2.723	hsa-miR-6501-3p
MIMAT0004492	1.75E-26	4.86E-27	10.9	50	2.721	hsa-miR-19b-2-5p
MIMAT0004975	8.13E-29	1.96E-29	11.4	55.5	2.721	hsa-miR-509-3-5p
MIMAT0027628	3.02E-28	7.51E-29	11.3	54.2	2.717	hsa-miR-6864-5p
MIMAT0022279	7.22E-37	1.12E-37	13	74.3	2.715	hsa-miR-5582-5p
MIMAT0003234	1.99E-31	4.11E-32	11.9	61.6	2.714	hsa-miR-569
MIMAT0019801	2.54E-46	2.46E-47	14.7	96.4	2.711	hsa-miR-4703-5p
MIMAT0019790	1.51E-46	1.44E-47	14.8	97	2.71	hsa-miR-4696
MIMAT0017983	1.79E-49	1.45E-50	15.3	104	2.705	hsa-miR-3606-5p
MIMAT0019947	1.41E-182	4.94E-185	31.3	412	2.703	hsa-miR-4783-3p
MIMAT0002881	2.83E-29	6.66E-30	11.5	56.6	2.703	hsa-miR-509-3p
MIMAT0017984	1.31E-41	1.59E-42	13.9	85.4	2.699	hsa-miR-3607-5p
MIMAT0002861	1.99E-19	7.61E-20	9.19	33.7	2.698	hsa-miR-518e-3p
MIMAT0018189	4.07E-30	9.22E-31	11.7	58.5	2.695	hsa-miR-3915
MIMAT0003165	2.76E-30	6.16E-31	11.7	58.9	2.695	hsa-miR-545-3p
MIMAT0025466	7.16E-28	1.83E-28	11.2	53.3	2.693	hsa-miR-6505-5p
MIMAT0030424	1.04E-25	2.95E-26	10.7	48.3	2.693	hsa-miR-7849-3p
MIMAT0003164	1.33E-25	3.79E-26	10.7	48	2.693	hsa-miR-544a
MIMAT0001635	3.55E-21	1.23E-21	9.63	37.7	2.691	hsa-miR-452-5p
MIMAT0026765	3.27E-45	3.35E-46	14.5	93.8	2.685	hsa-miR-1537-5p
MIMAT0004916	1.14E-32	2.24E-33	12.2	64.5	2.684	hsa-miR-888-5p
MIMAT0000431	8.90E-24	2.74E-24	10.3	43.8	2.683	hsa-miR-140-5p

MIMAT0022270	1.54E-45	1.56E-46	14.6	94.6	2.682	hsa-miR-5579-3p
MIMAT0000453	3.31E-29	7.82E-30	11.5	56.4	2.681	hsa-miR-154-3p
MIMAT0018447, M	3.06E-34	5.44E-35	12.5	68.2	2.68	hsa-miR-548aa, hsa-miR-548t-3p
MIMAT0002833	2.13E-23	6.69E-24	10.2	42.9	2.677	hsa-miR-520a-5p
MIMAT0019052	3.80E-50	2.93E-51	15.4	105	2.671	hsa-miR-4515
MIMAT0015033	6.57E-28	1.67E-28	11.2	53.4	2.666	hsa-miR-3159
MIMAT0003267	1.07E-26	2.96E-27	10.9	50.5	2.665	hsa-miR-599
MIMAT0018988	9.23E-53	6.48E-54	15.8	112	2.663	hsa-miR-4464
MIMAT0005945	5.39E-45	5.61E-46	14.5	93.3	2.663	hsa-miR-1255b-5p
MIMAT0015039	8.60E-35	1.48E-35	12.6	69.5	2.66	hsa-miR-3165
MIMAT0000076	2.22E-19	8.50E-20	9.17	33.6	2.653	hsa-miR-21-5p
MIMAT0004763	2.58E-30	5.72E-31	11.7	59	2.646	hsa-miR-488-3p
MIMAT0005909	2.32E-51	1.73E-52	15.6	108	2.639	hsa-miR-1258
MIMAT0027379	5.13E-34	9.28E-35	12.5	67.7	2.639	hsa-miR-6739-5p
MIMAT0022861	1.52E-51	1.11E-52	15.6	109	2.639	hsa-miR-376c-5p
MIMAT0004805	3.07E-22	1.00E-22	9.9	40.2	2.638	hsa-miR-616-3p
MIMAT0003289	1.74E-50	1.31E-51	15.4	106	2.638	hsa-miR-620
MIMAT0000241	1.62E-40	2.08E-41	13.7	82.9	2.637	hsa-miR-208a-3p
MIMAT0019897	3.93E-44	4.29E-45	14.3	91.3	2.633	hsa-miR-499b-5p
MIMAT0004798	9.25E-44	1.03E-44	14.3	90.4	2.629	hsa-miR-548b-5p
MIMAT0004506	1.65E-27	4.33E-28	11.1	52.4	2.626	hsa-miR-33a-3p
MIMAT0002839	5.20E-21	1.81E-21	9.59	37.4	2.625	hsa-miR-525-3p
MIMAT0000458	2.36E-33	4.42E-34	12.3	66.1	2.624	hsa-miR-190a-5p
MIMAT0019019	1.41E-20	5.07E-21	9.48	36.3	2.621	hsa-miR-4485-3p
MIMAT0016877	1.07E-40	1.37E-41	13.7	83.3	2.621	hsa-miR-4256
MIMAT0000680	3.19E-20	1.17E-20	9.39	35.5	2.621	hsa-miR-106b-5p
MIMAT0016855	5.24E-28	1.32E-28	11.2	53.6	2.62	hsa-miR-4302
MIMAT0005791	1.22E-39	1.64E-40	13.5	80.8	2.617	hsa-miR-1264
MIMAT0018954	1.28E-29	2.96E-30	11.6	57.4	2.616	hsa-miR-548ae-3p
MIMAT0004690	9.42E-26	2.67E-26	10.7	48.4	2.616	hsa-miR-379-3p
MIMAT0005885	1.44E-22	4.66E-23	9.97	41	2.614	hsa-miR-1295a
MIMAT0019725	5.44E-25	1.59E-25	10.5	46.6	2.614	hsa-miR-4658
MIMAT0004796	2.82E-43	3.22E-44	14.2	89.3	2.613	hsa-miR-576-3p
MIMAT0000730	2.89E-19	1.11E-19	9.14	33.3	2.612	hsa-miR-377-3p
MIMAT0000260	3.45E-20	1.27E-20	9.38	35.4	2.611	hsa-miR-182-3p
MIMAT0004765	1.66E-29	3.87E-30	11.5	57.1	2.611	hsa-miR-491-3p
MIMAT0016905	2.00E-26	5.59E-27	10.9	49.9	2.611	hsa-miR-4275
MIMAT0019869	1.61E-47	1.47E-48	14.9	99.2	2.609	hsa-miR-4740-5p
MIMAT0019914	8.15E-44	9.02E-45	14.3	90.6	2.608	hsa-miR-4764-5p
MIMAT0000259	2.07E-21	7.08E-22	9.69	38.3	2.606	hsa-miR-182-5p
MIMAT0022300	1.69E-32	3.36E-33	12.2	64.1	2.606	hsa-miR-5590-3p
MIMAT0000078	1.60E-35	2.66E-36	12.8	71.2	2.605	hsa-miR-23a-3p
MIMAT0004901	9.61E-24	2.96E-24	10.3	43.7	2.603	hsa-miR-298
MIMAT0003247	1.39E-22	4.49E-23	9.98	41	2.603	hsa-miR-582-5p
MIMAT0004689	1.57E-25	4.50E-26	10.7	47.8	2.602	hsa-miR-377-5p
MIMAT0003223	1.05E-34	1.82E-35	12.6	69.3	2.602	hsa-miR-559
MIMAT0019937	1.28E-36	2.00E-37	13	73.8	2.598	hsa-miR-4778-3p
MIMAT0018972	6.05E-58	3.42E-59	16.6	124	2.597	hsa-miR-548ah-5p
MIMAT0022269	2.06E-40	2.65E-41	13.7	82.6	2.595	hsa-miR-5579-5p
MIMAT0019736	2.46E-37	3.78E-38	13.1	75.4	2.595	hsa-miR-4662b
MIMAT0027571	1.18E-21	4.01E-22	9.75	38.8	2.594	hsa-miR-6835-3p
MIMAT0003282	4.55E-90	1.06E-91	21.1	198	2.594	hsa-miR-614
MIMAT0019703	3.63E-19	1.41E-19	9.12	33	2.594	hsa-miR-4643
MIMAT0030988	1.20E-44	1.27E-45	14.4	92.5	2.593	hsa-miR-8061
MIMAT0019716	3.60E-22	1.19E-22	9.88	40	2.588	hsa-miR-4652-5p
MIMAT0011162	2.27E-27	6.04E-28	11.1	52.1	2.586	hsa-miR-2117
MIMAT0005914	1.00E-53	6.77E-55	15.9	114	2.586	hsa-miR-1262

MIMAT0004764	5.29E-25	1.55E-25	10.5	46.6	2.585	hsa-miR-490-5p
MIMAT0019962	4.60E-23	1.45E-23	10.1	42.1	2.584	hsa-miR-4790-3p
MIMAT0022722, M	2.35E-33	4.38E-34	12.3	66.1	2.574	hsa-miR-548g-5p, hsa-miR-548x-5p, hsa-miR-548aj-5p
MIMAT0018946	1.80E-39	2.43E-40	13.5	80.4	2.573	hsa-miR-548ad-3p
MIMAT0005951	8.63E-154	4.71E-156	28.3	346	2.573	hsa-miR-1307-3p
MIMAT0015042	4.48E-35	7.59E-36	12.7	70.2	2.572	hsa-miR-3167
MIMAT0030983	2.64E-19	1.02E-19	9.15	33.4	2.571	hsa-miR-8056
MIMAT0005933	5.24E-46	5.15E-47	14.7	95.7	2.569	hsa-miR-1277-3p
MIMAT0022304	1.26E-38	1.80E-39	13.3	78.4	2.564	hsa-miR-548av-3p
MIMAT0004922	9.98E-49	8.68E-50	15.1	102	2.562	hsa-miR-875-5p
MIMAT0001545	6.22E-23	1.98E-23	10.1	41.8	2.562	hsa-miR-450a-5p
MIMAT0018989, M	8.31E-40	1.11E-40	13.6	81.2	2.559	hsa-miR-548ai, hsa-miR-570-5p
MIMAT0000251	2.82E-23	8.89E-24	10.1	42.6	2.559	hsa-miR-147a
MIMAT0019765	8.44E-28	2.17E-28	11.2	53.1	2.558	hsa-miR-4680-3p
MIMAT0016861	6.69E-28	1.71E-28	11.2	53.4	2.558	hsa-miR-4308
MIMAT0014977	1.70E-54	1.11E-55	16.1	116	2.556	hsa-miR-3115
MIMAT0019752	1.97E-31	4.05E-32	11.9	61.6	2.556	hsa-miR-4671-5p
MIMAT0005944	1.17E-29	2.71E-30	11.6	57.5	2.554	hsa-miR-1252-5p
MIMAT0000275	4.83E-24	1.47E-24	10.3	44.4	2.551	hsa-miR-218-5p
MIMAT0019070	1.68E-23	5.24E-24	10.2	43.1	2.551	hsa-miR-4531
MIMAT0000735	7.56E-20	2.82E-20	9.3	34.6	2.545	hsa-miR-380-3p
MIMAT0022696	2.55E-24	7.66E-25	10.4	45	2.543	hsa-miR-301a-5p
MIMAT0019890	3.69E-25	1.07E-25	10.6	47	2.54	hsa-miR-4753-5p
MIMAT0002841	2.22E-20	8.08E-21	9.43	35.9	2.54	hsa-miR-518f-5p
MIMAT0019974	1.85E-30	4.06E-31	11.7	59.4	2.537	hsa-miR-4798-5p
MIMAT0031003	5.04E-41	6.31E-42	13.8	84.1	2.533	hsa-miR-8076
MIMAT0015016	1.71E-31	3.48E-32	12	61.8	2.533	hsa-miR-3145-3p
MIMAT0019076	7.14E-27	1.95E-27	11	51	2.533	hsa-miR-548am-3p
MIMAT0018077	5.62E-30	1.28E-30	11.6	58.2	2.531	hsa-miR-3657
MIMAT0014981	2.14E-36	3.42E-37	12.9	73.2	2.531	hsa-miR-3119
MIMAT0004606	1.37E-21	4.67E-22	9.73	38.7	2.53	hsa-miR-136-3p
MIMAT0005917	2.02E-40	2.60E-41	13.7	82.7	2.529	hsa-miR-548m
MIMAT0002874	7.51E-22	2.53E-22	9.8	39.3	2.526	hsa-miR-503-5p
MIMAT0004925	4.40E-31	9.27E-32	11.9	60.8	2.526	hsa-miR-876-3p
MIMAT0014987	8.62E-19	3.40E-19	9.02	32.2	2.524	hsa-miR-548s
MIMAT0005912	3.14E-30	7.06E-31	11.7	58.8	2.524	hsa-miR-548g-3p
MIMAT0005913	9.12E-41	1.16E-41	13.7	83.5	2.524	hsa-miR-1261
MIMAT0018202	3.19E-39	4.34E-40	13.5	79.9	2.52	hsa-miR-3927-3p
MIMAT0000456	1.67E-21	5.71E-22	9.71	38.5	2.519	hsa-miR-186-5p
MIMAT0018108	7.54E-28	1.94E-28	11.2	53.2	2.518	hsa-miR-3681-5p
MIMAT0019934	3.02E-33	5.68E-34	12.3	65.9	2.518	hsa-miR-4777-5p
MIMAT0019936	1.67E-36	2.65E-37	12.9	73.5	2.514	hsa-miR-4778-5p
MIMAT0004562	8.03E-19	3.17E-19	9.03	32.3	2.512	hsa-miR-196a-3p
MIMAT0002809	1.96E-17	8.26E-18	8.65	29	2.511	hsa-miR-146b-5p
MIMAT0004596	1.69E-27	4.45E-28	11.1	52.4	2.508	hsa-miR-138-2-3p
MIMAT0004501	2.39E-16	1.05E-16	8.35	26.5	2.507	hsa-miR-27a-5p
MIMAT0019968	9.37E-45	9.82E-46	14.4	92.8	2.507	hsa-miR-4795-5p
MIMAT0022289	7.12E-22	2.39E-22	9.81	39.4	2.503	hsa-miR-5587-5p
MIMAT0019197	3.75E-42	4.46E-43	14	86.7	2.501	hsa-miR-3117-5p
MIMAT0019860	1.46E-28	3.56E-29	11.3	54.9	2.5	hsa-miR-4735-5p
MIMAT0011156	3.42E-30	7.71E-31	11.7	58.7	2.498	hsa-miR-2114-5p
MIMAT0004686	8.41E-24	2.58E-24	10.3	43.8	2.497	hsa-miR-367-5p
MIMAT0004921	3.46E-43	3.97E-44	14.2	89.1	2.497	hsa-miR-889-3p
MIMAT0019810	5.32E-53	3.65E-54	15.8	112	2.496	hsa-miR-4708-3p
MIMAT0018957	3.75E-28	9.40E-29	11.2	54	2.496	hsa-miR-4439

MIMAT0000442	5.42E-19	2.13E-19	9.07	32.6	2.495	hsa-miR-9-3p
MIMAT0019911	1.95E-29	4.56E-30	11.5	57	2.495	hsa-miR-4762-3p
MIMAT0015086	3.83E-35	6.49E-36	12.7	70.3	2.495	hsa-miR-3201
MIMAT0019907	2.63E-42	3.09E-43	14	87.1	2.493	hsa-miR-4760-3p
MIMAT0019960	1.13E-27	2.93E-28	11.1	52.8	2.492	hsa-miR-4789-3p
MIMAT0003293	3.23E-22	1.06E-22	9.89	40.2	2.492	hsa-miR-624-5p
MIMAT0021037	2.19E-30	4.81E-31	11.7	59.2	2.492	hsa-miR-548ap-5p
MIMAT0015037	3.42E-30	7.72E-31	11.7	58.7	2.492	hsa-miR-3163
MIMAT0017995	5.04E-27	1.36E-27	11	51.3	2.492	hsa-miR-3616-5p
MIMAT0022493	6.66E-26	1.88E-26	10.7	48.7	2.491	hsa-miR-5700
MIMAT0000446	1.94E-18	7.76E-19	8.93	31.4	2.49	hsa-miR-127-3p
MIMAT0025851	1.14E-35	1.88E-36	12.8	71.5	2.488	hsa-miR-6720-3p
MIMAT0000429	5.19E-18	2.12E-18	8.81	30.4	2.488	hsa-miR-137
MIMAT0026624	8.00E-43	9.27E-44	14.1	88.3	2.487	hsa-miR-651-3p
MIMAT0015030	1.47E-60	7.39E-62	17	130	2.483	hsa-miR-3156-5p
MIMAT0002875	9.43E-22	3.19E-22	9.77	39.1	2.482	hsa-miR-504-5p
MIMAT0004797	4.74E-24	1.44E-24	10.3	44.4	2.482	hsa-miR-582-3p
MIMAT0000684	1.22E-22	3.92E-23	9.99	41.1	2.478	hsa-miR-302a-3p
MIMAT0025480	6.48E-41	8.19E-42	13.8	83.8	2.477	hsa-miR-6512-5p
MIMAT0005870	8.61E-33	1.67E-33	12.2	64.8	2.475	hsa-miR-1206
MIMAT0022266	1.27E-20	4.54E-21	9.49	36.4	2.475	hsa-miR-548ar-3p
MIMAT0003225	3.04E-38	4.47E-39	13.3	77.5	2.47	hsa-miR-561-3p
MIMAT0020300	6.43E-24	1.97E-24	10.3	44.1	2.469	hsa-miR-4520-2-3p
MIMAT0014985	7.49E-29	1.79E-29	11.4	55.6	2.467	hsa-miR-3123
MIMAT0000693	9.36E-23	2.98E-23	10	41.4	2.466	hsa-miR-30e-3p
MIMAT0017988	3.23E-42	3.81E-43	14	86.9	2.465	hsa-miR-3611
MIMAT0022724	1.26E-29	2.91E-30	11.6	57.4	2.464	hsa-miR-1277-5p
MIMAT0004924	2.51E-24	7.51E-25	10.4	45.1	2.461	hsa-miR-876-5p
MIMAT0018097	6.93E-40	9.21E-41	13.6	81.4	2.459	hsa-miR-3674
MIMAT0022264	2.27E-27	6.04E-28	11.1	52.1	2.458	hsa-miR-548aq-3p
MIMAT0003389	4.12E-30	9.35E-31	11.7	58.5	2.456	hsa-miR-542-3p
MIMAT0018446, M	8.61E-33	1.68E-33	12.2	64.8	2.454	hsa-miR-548z, hsa-miR-548h-3p
MIMAT0017986	1.44E-34	2.51E-35	12.6	69	2.453	hsa-miR-3609
MIMAT0003386	5.48E-25	1.60E-25	10.5	46.6	2.452	hsa-miR-376a-5p
MIMAT0018186	1.92E-43	2.16E-44	14.2	89.7	2.452	hsa-miR-3912-3p
MIMAT0019080	2.07E-25	5.97E-26	10.6	47.6	2.451	hsa-miR-4537
MIMAT0000448	9.70E-20	3.65E-20	9.27	34.4	2.446	hsa-miR-136-5p
MIMAT0019943	4.96E-31	1.05E-31	11.9	60.7	2.443	hsa-miR-4781-3p
MIMAT0005907	1.02E-17	4.20E-18	8.73	29.7	2.442	hsa-miR-1256
MIMAT0004785	3.82E-28	9.58E-29	11.2	53.9	2.442	hsa-miR-545-5p
MIMAT0019872	8.89E-31	1.91E-31	11.8	60.1	2.441	hsa-miR-4742-5p
MIMAT0003325	1.63E-19	6.17E-20	9.21	33.9	2.44	hsa-miR-662
MIMAT0015046	9.16E-28	2.37E-28	11.2	53	2.44	hsa-miR-3171
MIMAT0018938	4.97E-24	1.51E-24	10.3	44.4	2.439	hsa-miR-548ac
MIMAT0015009	4.94E-25	1.44E-25	10.6	46.7	2.439	hsa-miR-548t-5p
MIMAT0028216	1.75E-19	6.66E-20	9.2	33.8	2.433	hsa-miR-7153-5p
MIMAT0015044	2.83E-29	6.66E-30	11.5	56.6	2.432	hsa-miR-3169
MIMAT0000439	5.67E-21	1.98E-21	9.58	37.3	2.431	hsa-miR-153-3p
MIMAT0000073	1.75E-28	4.29E-29	11.3	54.7	2.429	hsa-miR-19a-3p
MIMAT0003290	3.46E-27	9.29E-28	11	51.7	2.426	hsa-miR-621
MIMAT0000510	1.52E-147	1.01E-149	27.7	331	2.425	hsa-miR-320a
MIMAT0001625	5.25E-19	2.06E-19	9.08	32.7	2.424	hsa-miR-431-5p
MIMAT0018199	3.46E-40	4.53E-41	13.6	82.1	2.423	hsa-miR-3924
MIMAT0003328	1.16E-27	3.03E-28	11.1	52.8	2.422	hsa-miR-653-5p
MIMAT0003236	1.35E-20	4.85E-21	9.49	36.4	2.421	hsa-miR-571
MIMAT0000714	2.26E-16	9.92E-17	8.36	26.6	2.419	hsa-miR-302b-5p
MIMAT0002859	4.93E-20	1.83E-20	9.34	35.1	2.417	hsa-miR-516b-5p

MIMAT0004929	5.21E-33	9.97E-34	12.3	65.3	2.416	hsa-miR-190b
MIMAT0030418	2.03E-29	4.77E-30	11.5	56.9	2.415	hsa-miR-6516-3p
MIMAT0003232	5.19E-24	1.58E-24	10.3	44.3	2.415	hsa-miR-568
MIMAT0019963	3.51E-26	9.83E-27	10.8	49.4	2.414	hsa-miR-4791
MIMAT0019212	6.40E-39	9.01E-40	13.4	79.1	2.414	hsa-miR-3160-5p
MIMAT0004987	8.57E-16	3.88E-16	8.19	25.2	2.413	hsa-miR-944
MIMAT0005916	4.90E-33	9.36E-34	12.3	65.4	2.413	hsa-miR-548n
MIMAT0000424	2.93E-19	1.13E-19	9.14	33.3	2.412	hsa-miR-128-3p
MIMAT0027435	4.33E-27	1.16E-27	11	51.5	2.41	hsa-miR-6767-3p
MIMAT0023704	4.31E-32	8.63E-33	12.1	63.2	2.408	hsa-miR-6079
MIMAT0022471	1.34E-32	2.66E-33	12.2	64.3	2.408	hsa-miR-548aw
MIMAT0019984	2.76E-28	6.84E-29	11.3	54.3	2.403	hsa-miR-4804-5p
MIMAT0003338	1.24E-23	3.85E-24	10.2	43.4	2.402	hsa-miR-660-5p
MIMAT0005887	4.79E-22	1.60E-22	9.85	39.8	2.401	hsa-miR-1299
MIMAT0001340	5.78E-27	1.58E-27	11	51.2	2.4	hsa-miR-423-3p
MIMAT0027632	2.54E-30	5.63E-31	11.7	59	2.399	hsa-miR-6866-5p
MIMAT0022862	1.46E-23	4.52E-24	10.2	43.3	2.398	hsa-miR-381-5p
MIMAT0002828	5.38E-35	9.15E-36	12.7	70	2.396	hsa-miR-519e-5p
MIMAT0025846	4.53E-73	1.48E-74	18.8	159	2.394	hsa-miR-6717-5p
MIMAT0019961	9.89E-21	3.51E-21	9.52	36.7	2.393	hsa-miR-4790-5p
MIMAT0022263	1.13E-21	3.83E-22	9.76	38.9	2.392	hsa-miR-548aq-5p
MIMAT0001638	4.14E-16	1.84E-16	8.28	26	2.392	hsa-miR-409-5p
MIMAT0030993	1.11E-28	2.68E-29	11.4	55.2	2.391	hsa-miR-8066
MIMAT0003272	2.68E-22	8.75E-23	9.91	40.3	2.389	hsa-miR-604
MIMAT0018443	4.04E-25	1.18E-25	10.6	46.9	2.389	hsa-miR-374c-5p
MIMAT0019898	3.29E-22	1.08E-22	9.89	40.1	2.388	hsa-miR-499b-3p
MIMAT0030427	2.18E-31	4.52E-32	11.9	61.5	2.387	hsa-miR-7852-3p
MIMAT0003327	5.18E-23	1.64E-23	10.1	42	2.386	hsa-miR-449b-5p
MIMAT0021131	7.00E-31	1.49E-31	11.8	60.3	2.382	hsa-miR-5197-3p
MIMAT0006767	2.15E-31	4.43E-32	11.9	61.6	2.382	hsa-miR-1827
MIMAT0009978	1.35E-31	2.73E-32	12	62	2.375	hsa-miR-2053
MIMAT0019005	2.11E-36	3.37E-37	12.9	73.3	2.374	hsa-miR-4477b
MIMAT0019234	5.78E-24	1.77E-24	10.3	44.2	2.373	hsa-miR-4474-5p
MIMAT0019951	2.12E-34	3.74E-35	12.5	68.6	2.372	hsa-miR-1245b-3p
MIMAT0015013	1.16E-34	2.02E-35	12.6	69.2	2.372	hsa-miR-548u
MIMAT0004974	5.97E-37	9.22E-38	13	74.5	2.371	hsa-miR-924
MIMAT0020541	3.23E-34	5.76E-35	12.5	68.1	2.37	hsa-miR-5047
MIMAT0020959	9.31E-37	1.44E-37	13	74.1	2.369	hsa-miR-4536-3p
MIMAT0019945	2.67E-25	7.71E-26	10.6	47.3	2.368	hsa-miR-4782-3p
MIMAT0004807	1.94E-19	7.42E-20	9.19	33.7	2.367	hsa-miR-624-3p
MIMAT0019013	1.86E-38	2.68E-39	13.3	78.1	2.367	hsa-miR-548ak
MIMAT0013517	4.82E-22	1.61E-22	9.85	39.7	2.361	hsa-miR-2682-5p
MIMAT0018353	3.96E-20	1.46E-20	9.37	35.3	2.36	hsa-miR-3938
MIMAT0022277	6.02E-29	1.44E-29	11.4	55.8	2.356	hsa-miR-548at-5p
MIMAT0019851	5.22E-20	1.94E-20	9.34	35	2.356	hsa-miR-4729
MIMAT0004556	3.45E-25	1.00E-25	10.6	47.1	2.355	hsa-miR-10b-3p
MIMAT0019016	1.49E-34	2.61E-35	12.6	68.9	2.355	hsa-miR-4482-5p
MIMAT0014991	8.85E-36	1.44E-36	12.8	71.8	2.354	hsa-miR-3128
MIMAT0022712	1.30E-38	1.86E-39	13.3	78.4	2.352	hsa-miR-1271-3p
MIMAT0000727	2.04E-20	7.40E-21	9.44	36	2.351	hsa-miR-374a-5p
MIMAT0003281	2.57E-20	9.38E-21	9.42	35.7	2.351	hsa-miR-613
MIMAT0003275	8.81E-33	1.72E-33	12.2	64.8	2.35	hsa-miR-607
MIMAT0004543	3.66E-16	1.62E-16	8.3	26.1	2.35	hsa-miR-192-3p
MIMAT0019761	1.26E-28	3.06E-29	11.4	55.1	2.35	hsa-miR-4677-3p
MIMAT0004512	3.42E-23	1.08E-23	10.1	42.4	2.344	hsa-miR-100-3p
MIMAT0003265	1.60E-17	6.69E-18	8.68	29.2	2.343	hsa-miR-597-5p
MIMAT0001629	8.96E-19	3.55E-19	9.01	32.1	2.337	hsa-miR-329-3p
MIMAT0019971	2.39E-20	8.69E-21	9.42	35.8	2.337	hsa-miR-4796-3p

MIMAT0000273	6.58E-18	2.70E-18	8.78	30.1	2.337	hsa-miR-216a-5p
MIMAT0022291	1.00E-39	1.34E-40	13.6	81	2.336	hsa-miR-548au-5p
MIMAT0018974	1.09E-30	2.35E-31	11.8	59.9	2.336	hsa-miR-4452
MIMAT0003274	1.59E-32	3.15E-33	12.2	64.2	2.335	hsa-miR-606
MIMAT0005932	7.31E-35	1.25E-35	12.6	69.7	2.335	hsa-miR-302f
MIMAT0004600	1.63E-33	3.00E-34	12.4	66.5	2.331	hsa-miR-144-5p
MIMAT0000452	8.89E-21	3.13E-21	9.53	36.8	2.331	hsa-miR-154-5p
MIMAT0000692	1.70E-17	7.10E-18	8.67	29.2	2.33	hsa-miR-30e-5p
MIMAT0004907	1.37E-21	4.68E-22	9.73	38.7	2.33	hsa-miR-892a
MIMAT0003268	9.72E-21	3.44E-21	9.52	36.7	2.329	hsa-miR-600
MIMAT0000428	1.00E-18	3.98E-19	9	32	2.328	hsa-miR-135a-5p
MIMAT0005934	8.06E-29	1.94E-29	11.4	55.5	2.327	hsa-miR-548p
MIMAT0019824	7.13E-30	1.63E-30	11.6	58	2.327	hsa-miR-4715-5p
MIMAT0004568	3.53E-31	7.41E-32	11.9	61	2.324	hsa-miR-221-5p
MIMAT0019905	1.19E-27	3.09E-28	11.1	52.8	2.322	hsa-miR-4759
MIMAT0003297	7.71E-20	2.88E-20	9.29	34.6	2.322	hsa-miR-628-3p
MIMAT0019931	1.54E-36	2.44E-37	13	73.6	2.32	hsa-miR-4775
MIMAT0009197	4.71E-28	1.19E-28	11.2	53.7	2.319	hsa-miR-205-3p
MIMAT0005896	2.05E-37	3.13E-38	13.1	75.6	2.317	hsa-miR-1244
MIMAT0019039	1.40E-14	6.76E-15	7.83	22.4	2.316	hsa-miR-4503
MIMAT0019957	3.83E-187	7.47E-190	31.7	424	2.316	hsa-miR-4787-3p
MIMAT0004588	1.52E-25	4.35E-26	10.7	47.9	2.314	hsa-miR-27b-5p
MIMAT0004927	4.06E-22	1.35E-22	9.86	39.9	2.314	hsa-miR-708-3p
MIMAT0019689	1.19E-21	4.04E-22	9.75	38.8	2.312	hsa-miR-4633-5p
MIMAT0022928	1.02E-29	2.35E-30	11.6	57.6	2.312	hsa-miR-376a-2-5p
MIMAT0000454	6.79E-25	2.00E-25	10.5	46.4	2.311	hsa-miR-184
MIMAT0019825	1.57E-15	7.17E-16	8.11	24.6	2.311	hsa-miR-4715-3p
MIMAT0030985	9.98E-18	4.13E-18	8.73	29.7	2.311	hsa-miR-8058
MIMAT0019823	1.49E-22	4.83E-23	9.97	40.9	2.311	hsa-miR-4714-3p
MIMAT0000720	1.85E-17	7.79E-18	8.66	29.1	2.31	hsa-miR-376c-3p
MIMAT0005824	4.55E-20	1.68E-20	9.35	35.2	2.309	hsa-miR-1179
MIMAT0019920	1.72E-24	5.12E-25	10.4	45.4	2.308	hsa-miR-4768-5p
MIMAT0004752	3.02E-22	9.88E-23	9.9	40.2	2.308	hsa-miR-20b-3p
MIMAT0004779	3.28E-19	1.27E-19	9.13	33.2	2.307	hsa-miR-509-5p
MIMAT0022299	6.22E-24	1.90E-24	10.3	44.1	2.306	hsa-miR-5590-5p
MIMAT0000253	7.59E-21	2.66E-21	9.55	37	2.304	hsa-miR-10a-5p
MIMAT0000256	4.09E-18	1.66E-18	8.84	30.6	2.304	hsa-miR-181a-5p
MIMAT0019693	8.82E-22	2.98E-22	9.78	39.1	2.303	hsa-miR-4636
MIMAT0003301	1.85E-19	7.05E-20	9.19	33.7	2.3	hsa-miR-33b-5p
MIMAT0005935	4.59E-38	6.85E-39	13.2	77.1	2.299	hsa-miR-548i
MIMAT0022498	1.23E-20	4.39E-21	9.5	36.5	2.298	hsa-miR-5704
MIMAT0004774	1.56E-26	4.33E-27	10.9	50.2	2.296	hsa-miR-501-3p
MIMAT0018184	1.49E-31	3.03E-32	12	61.9	2.296	hsa-miR-3910
MIMAT0022978	5.42E-22	1.82E-22	9.83	39.6	2.294	hsa-miR-4743-3p
MIMAT0016879	2.13E-194	3.32E-197	32.5	440	2.294	hsa-miR-4258
MIMAT0003264	5.69E-23	1.80E-23	10.1	41.9	2.293	hsa-miR-596
MIMAT0021084	3.57E-18	1.44E-18	8.85	30.8	2.292	hsa-miR-5092
MIMAT0003150	8.48E-29	2.05E-29	11.4	55.5	2.291	hsa-miR-455-5p
MIMAT0019768	3.35E-19	1.30E-19	9.13	33.1	2.291	hsa-miR-4683
MIMAT0019795	2.38E-26	6.63E-27	10.8	49.7	2.29	hsa-miR-4699-3p
MIMAT0018193	3.12E-16	1.38E-16	8.32	26.3	2.289	hsa-miR-3919
MIMAT0005890	4.39E-14	2.17E-14	7.68	21.3	2.289	hsa-miR-1302
MIMAT0000276	2.91E-19	1.12E-19	9.14	33.3	2.289	hsa-miR-219a-5p
MIMAT0005904	5.16E-24	1.57E-24	10.3	44.3	2.289	hsa-miR-1253
MIMAT0004555	7.95E-21	2.79E-21	9.55	36.9	2.288	hsa-miR-10a-3p
MIMAT0022280	1.62E-25	4.66E-26	10.7	47.8	2.287	hsa-miR-5582-3p
MIMAT0000095	1.97E-16	8.62E-17	8.37	26.7	2.285	hsa-miR-96-5p
MIMAT0031175	3.61E-33	6.82E-34	12.3	65.7	2.284	hsa-miR-548ba

MIMAT0004956	3.04E-30	6.81E-31	11.7	58.8	2.284	hsa-miR-374b-3p
MIMAT0018969	2.22E-31	4.62E-32	11.9	61.5	2.283	hsa-miR-548ag
MIMAT0005955	1.05E-29	2.42E-30	11.6	57.6	2.282	hsa-miR-1197
MIMAT0005906	9.29E-19	3.68E-19	9.01	32.1	2.282	hsa-miR-1255a
MIMAT0000417	2.44E-19	9.36E-20	9.16	33.5	2.281	hsa-miR-15b-5p
MIMAT0003242	5.95E-21	2.08E-21	9.58	37.2	2.281	hsa-miR-577
MIMAT0018358	1.56E-29	3.62E-30	11.5	57.2	2.28	hsa-miR-3942-5p
MIMAT0018930	3.67E-15	1.72E-15	8.01	23.8	2.279	hsa-miR-4418
MIMAT0026744	2.19E-22	7.12E-23	9.93	40.6	2.277	hsa-miR-1252-3p
MIMAT0013516	1.01E-20	3.57E-21	9.52	36.7	2.276	hsa-miR-2681-3p
MIMAT0004910	3.08E-20	1.13E-20	9.4	35.5	2.276	hsa-miR-450b-3p
MIMAT0019834	3.02E-28	7.50E-29	11.3	54.2	2.276	hsa-miR-4720-3p
MIMAT0002843	1.53E-14	7.43E-15	7.82	22.3	2.275	hsa-miR-520b
MIMAT0019910	5.25E-19	2.05E-19	9.08	32.7	2.273	hsa-miR-4762-5p
MIMAT0019030	1.39E-27	3.66E-28	11.1	52.6	2.272	hsa-miR-4495
MIMAT0005798	1.83E-30	4.00E-31	11.7	59.4	2.27	hsa-miR-1185-5p
MIMAT0015025	9.49E-26	2.70E-26	10.7	48.4	2.27	hsa-miR-3152-3p
MIMAT0004772	1.61E-25	4.63E-26	10.7	47.8	2.268	hsa-miR-499a-3p
MIMAT0025843	1.06E-22	3.39E-23	10	41.3	2.268	hsa-miR-6715b-3p
MIMAT0002806	1.25E-14	6.01E-15	7.85	22.5	2.267	hsa-miR-490-3p
MIMAT0005895	8.27E-17	3.56E-17	8.48	27.6	2.262	hsa-miR-548f-3p
MIMAT0018357	1.66E-33	3.06E-34	12.4	66.5	2.262	hsa-miR-3941
MIMAT0019220	1.76E-26	4.90E-27	10.9	50	2.262	hsa-miR-3664-3p
MIMAT0019944	2.19E-38	3.21E-39	13.3	77.9	2.261	hsa-miR-4782-5p
MIMAT0003292	6.92E-27	1.89E-27	11	51	2.26	hsa-miR-623
MIMAT0022476	1.68E-22	5.46E-23	9.96	40.8	2.259	hsa-miR-5692c
MIMAT0018086	1.09E-20	3.90E-21	9.51	36.6	2.257	hsa-miR-3664-5p
MIMAT0019927	1.65E-20	5.96E-21	9.46	36.2	2.256	hsa-miR-4772-3p
MIMAT0004912	2.75E-22	8.97E-23	9.91	40.3	2.255	hsa-miR-890
MIMAT0027036	1.85E-30	4.06E-31	11.7	59.4	2.255	hsa-miR-3912-5p
MIMAT0002827	1.46E-18	5.82E-19	8.96	31.7	2.253	hsa-miR-515-3p
MIMAT0030020	2.83E-25	8.21E-26	10.6	47.2	2.252	hsa-miR-7705
MIMAT0016912	3.74E-24	1.13E-24	10.3	44.7	2.251	hsa-miR-4282
MIMAT0027367	3.67E-19	1.43E-19	9.12	33	2.248	hsa-miR-6733-5p
MIMAT0026625	1.82E-30	3.96E-31	11.7	59.4	2.246	hsa-miR-653-3p
MIMAT0002826	4.19E-14	2.07E-14	7.69	21.3	2.245	hsa-miR-515-5p
MIMAT0031000	4.51E-177	1.76E-179	30.7	400	2.244	hsa-miR-8073
MIMAT0004960	5.14E-18	2.10E-18	8.81	30.4	2.238	hsa-miR-208b-3p
MIMAT0004509	4.19E-18	1.70E-18	8.84	30.6	2.238	hsa-miR-93-3p
MIMAT0032114, M	9.00E-29	2.18E-29	11.4	55.4	2.237	hsa-miR-548ad-5p, hsa-miR-548ae-5p
MIMAT0003226	1.93E-27	5.11E-28	11.1	52.3	2.236	hsa-miR-562
MIMAT0018990	2.59E-20	9.46E-21	9.41	35.7	2.236	hsa-miR-548aj-3p
MIMAT0019753	2.19E-21	7.52E-22	9.68	38.2	2.233	hsa-miR-4671-3p
MIMAT0019228	1.95E-26	5.43E-27	10.9	49.9	2.232	hsa-miR-3925-3p
MIMAT0018188	2.16E-24	6.44E-25	10.4	45.2	2.231	hsa-miR-3914
MIMAT0022278	6.55E-23	2.08E-23	10.1	41.8	2.231	hsa-miR-548at-3p
MIMAT0026737	1.00E-29	2.31E-30	11.6	57.6	2.231	hsa-miR-548j-3p
MIMAT0019734	9.35E-27	2.58E-27	10.9	50.7	2.23	hsa-miR-4659b-3p
MIMAT0016908	1.17E-22	3.75E-23	10	41.2	2.23	hsa-miR-4277
MIMAT0026476	1.50E-19	5.70E-20	9.22	33.9	2.23	hsa-miR-215-3p
MIMAT0014980	7.68E-34	1.40E-34	12.4	67.3	2.229	hsa-miR-3118
MIMAT0004601	1.65E-29	3.85E-30	11.5	57.1	2.226	hsa-miR-145-3p
MIMAT0030981	6.36E-20	2.36E-20	9.31	34.8	2.223	hsa-miR-8054
MIMAT0019977	4.75E-24	1.44E-24	10.3	44.4	2.222	hsa-miR-4799-3p
MIMAT0000441	1.77E-21	6.04E-22	9.71	38.4	2.217	hsa-miR-9-5p
MIMAT0015020	1.39E-23	4.32E-24	10.2	43.3	2.217	hsa-miR-548v
MIMAT0018998	3.08E-22	1.01E-22	9.89	40.2	2.213	hsa-miR-4471

MIMAT0019895	4.18E-22	1.39E-22	9.86	39.9	2.212	hsa-miR-4755-5p
MIMAT0026736	5.74E-28	1.45E-28	11.2	53.5	2.212	hsa-miR-548e-5p
MIMAT0019804	4.61E-19	1.80E-19	9.09	32.8	2.211	hsa-miR-4704-3p
MIMAT0015008	2.38E-23	7.47E-24	10.2	42.8	2.21	hsa-miR-3140-3p
MIMAT0002817	8.33E-16	3.77E-16	8.19	25.3	2.205	hsa-miR-495-3p
MIMAT0018117, M	3.87E-29	9.15E-30	11.5	56.3	2.205	hsa-miR-3689a-5p, hsa-miR-3689b-5p, hsa-miR-3689e
MIMAT0022481	8.00E-22	2.70E-22	9.79	39.2	2.202	hsa-miR-5689
MIMAT0003330	1.28E-27	3.35E-28	11.1	52.7	2.2	hsa-miR-654-5p
MIMAT0028234	1.57E-20	5.67E-21	9.47	36.2	2.2	hsa-miR-7162-5p
MIMAT0019902	4.51E-16	2.02E-16	8.27	25.9	2.2	hsa-miR-4757-3p
MIMAT0026640	8.14E-21	2.86E-21	9.54	36.9	2.199	hsa-miR-670-3p
MIMAT0002821	2.10E-17	8.86E-18	8.64	29	2.197	hsa-miR-181d-5p
MIMAT0019926	1.05E-18	4.19E-19	9	32	2.196	hsa-miR-4772-5p
MIMAT0004777	5.31E-21	1.85E-21	9.59	37.3	2.195	hsa-miR-513a-3p
MIMAT0009979	2.50E-28	6.18E-29	11.3	54.4	2.193	hsa-miR-2054
MIMAT0000721	1.97E-18	7.91E-19	8.92	31.4	2.192	hsa-miR-369-3p
MIMAT0002860, M	3.17E-15	1.47E-15	8.02	23.9	2.192	hsa-miR-516b-3p, hsa-miR-516a-3p
MIMAT0026626	6.72E-28	1.72E-28	11.2	53.4	2.189	hsa-miR-655-5p
MIMAT0016853	8.89E-20	3.34E-20	9.28	34.5	2.189	hsa-miR-4300
MIMAT0003245	7.77E-17	3.34E-17	8.49	27.7	2.187	hsa-miR-580-3p
MIMAT0017990	2.82E-35	4.71E-36	12.7	70.6	2.187	hsa-miR-3613-5p
MIMAT0021122	9.86E-23	3.14E-23	10	41.4	2.187	hsa-miR-5191
MIMAT0000761	3.66E-18	1.48E-18	8.85	30.7	2.186	hsa-miR-324-5p
MIMAT0021042	1.19E-17	4.95E-18	8.71	29.5	2.186	hsa-miR-5009-3p
MIMAT0003271	4.36E-18	1.77E-18	8.83	30.6	2.184	hsa-miR-603
MIMAT0015063	3.97E-20	1.46E-20	9.37	35.3	2.181	hsa-miR-3183
MIMAT0022479	3.65E-18	1.48E-18	8.85	30.7	2.181	hsa-miR-5688
MIMAT0001075	4.93E-16	2.21E-16	8.26	25.8	2.181	hsa-miR-384
MIMAT0022470	1.24E-32	2.45E-33	12.2	64.4	2.179	hsa-miR-5682
MIMAT0005903	1.80E-16	7.86E-17	8.38	26.8	2.178	hsa-miR-1251-5p
MIMAT0030980	1.01E-19	3.82E-20	9.26	34.3	2.178	hsa-miR-8053
MIMAT0019049	2.56E-21	8.83E-22	9.67	38.1	2.177	hsa-miR-4512
MIMAT0004955	1.30E-20	4.66E-21	9.49	36.4	2.175	hsa-miR-374b-5p
MIMAT0004928	2.34E-19	8.98E-20	9.17	33.5	2.174	hsa-miR-147b
MIMAT0000258	4.43E-18	1.80E-18	8.83	30.5	2.174	hsa-miR-181c-5p
MIMAT0000087	8.72E-17	3.76E-17	8.47	27.5	2.172	hsa-miR-30a-5p
MIMAT0019950	9.71E-21	3.43E-21	9.52	36.7	2.17	hsa-miR-1245b-5p
MIMAT0015029	1.54E-18	6.14E-19	8.95	31.6	2.169	hsa-miR-3155a
MIMAT0019930	8.45E-27	2.32E-27	10.9	50.8	2.169	hsa-miR-4774-3p
MIMAT0018114	5.61E-39	7.86E-40	13.4	79.3	2.168	hsa-miR-3686
MIMAT0000432	9.59E-33	1.88E-33	12.2	64.7	2.168	hsa-miR-141-3p
MIMAT0026478	7.55E-20	2.81E-20	9.3	34.6	2.167	hsa-miR-133a-5p
MIMAT0002825	2.91E-14	1.42E-14	7.73	21.7	2.166	hsa-miR-520e
MIMAT0000250	2.59E-18	1.04E-18	8.89	31.1	2.161	hsa-miR-139-5p
MIMAT0004503	9.45E-13	4.99E-13	7.26	18.2	2.158	hsa-miR-29a-5p
MIMAT0005874	3.58E-15	1.67E-15	8.01	23.8	2.158	hsa-miR-548e-3p
MIMAT0000728	1.22E-14	5.87E-15	7.85	22.6	2.157	hsa-miR-375
MIMAT0017998	2.35E-19	9.01E-20	9.17	33.5	2.155	hsa-miR-3618
MIMAT0022940	2.14E-17	9.02E-18	8.64	28.9	2.154	hsa-miR-1178-5p
MIMAT0002846	8.51E-13	4.48E-13	7.27	18.3	2.152	hsa-miR-520c-3p
MIMAT0002840	1.79E-14	8.71E-15	7.8	22.2	2.151	hsa-miR-523-3p
MIMAT0019815	2.76E-40	3.58E-41	13.7	82.3	2.15	hsa-miR-4710
MIMAT0021036	6.32E-25	1.85E-25	10.5	46.4	2.15	hsa-miR-5007-3p
MIMAT0022472	8.96E-19	3.54E-19	9.01	32.1	2.149	hsa-miR-5683
MIMAT0004587	1.91E-19	7.27E-20	9.19	33.7	2.147	hsa-miR-23b-5p

MIMAT0021030	5.37E-17	2.30E-17	8.53	28	2.146	hsa-miR-548ao-3p
MIMAT0003296	3.96E-21	1.37E-21	9.62	37.6	2.146	hsa-miR-627-5p
MIMAT0005799	3.51E-20	1.29E-20	9.38	35.4	2.145	hsa-miR-1283
MIMAT0003879	1.23E-20	4.40E-21	9.5	36.5	2.145	hsa-miR-758-3p
MIMAT0002856	1.44E-15	6.57E-16	8.13	24.7	2.144	hsa-miR-520d-3p
MIMAT0015066	2.44E-15	1.13E-15	8.06	24.2	2.144	hsa-miR-3065-5p
MIMAT0003311	4.00E-17	1.70E-17	8.57	28.3	2.143	hsa-miR-641
MIMAT0005936	3.34E-19	1.29E-19	9.13	33.1	2.143	hsa-miR-1278
MIMAT0026613	5.47E-21	1.91E-21	9.59	37.3	2.142	hsa-miR-510-3p
MIMAT0019866	7.91E-22	2.66E-22	9.79	39.2	2.142	hsa-miR-4738-5p
MIMAT0018963	2.08E-12	1.12E-12	7.15	17.4	2.142	hsa-miR-4445-5p
MIMAT0000729	6.89E-13	3.61E-13	7.3	18.5	2.141	hsa-miR-376a-3p
MIMAT0000244	2.37E-17	1.00E-17	8.63	28.8	2.141	hsa-miR-30c-5p
MIMAT0025456	2.33E-24	6.98E-25	10.4	45.1	2.139	hsa-miR-548az-5p
MIMAT0004675	2.07E-15	9.50E-16	8.08	24.4	2.138	hsa-miR-219a-2-3p
MIMAT0018196	1.37E-23	4.24E-24	10.2	43.3	2.135	hsa-miR-3921
MIMAT0004481	8.59E-20	3.22E-20	9.28	34.5	2.135	hsa-let-7a-3p
MIMAT0028227	1.48E-23	4.62E-24	10.2	43.3	2.135	hsa-miR-7158-3p
MIMAT0000685	2.30E-17	9.73E-18	8.63	28.9	2.135	hsa-miR-34b-5p
MIMAT0019842	1.14E-15	5.18E-16	8.15	25	2.134	hsa-miR-4724-3p
MIMAT0004923	1.05E-18	4.17E-19	9	32	2.129	hsa-miR-875-3p
MIMAT0005940	4.14E-21	1.44E-21	9.62	37.6	2.125	hsa-miR-1282
MIMAT0014978	1.93E-16	8.47E-17	8.38	26.7	2.123	hsa-miR-3116
MIMAT0025473	1.97E-20	7.12E-21	9.44	36	2.121	hsa-miR-6508-3p
MIMAT0019976	2.06E-16	9.04E-17	8.37	26.7	2.12	hsa-miR-4799-5p
MIMAT0021025	1.86E-20	6.72E-21	9.45	36.1	2.119	hsa-miR-5003-5p
MIMAT0004612	6.93E-19	2.73E-19	9.04	32.4	2.117	hsa-miR-186-3p
MIMAT0032026	9.43E-19	3.74E-19	9.01	32.1	2.115	hsa-miR-301b-5p
MIMAT0000103	2.33E-15	1.07E-15	8.06	24.2	2.115	hsa-miR-106a-5p
MIMAT0018976	8.78E-91	1.95E-92	21.2	200	2.114	hsa-miR-4454
MIMAT0022469	2.41E-20	8.76E-21	9.42	35.8	2.114	hsa-miR-5681a
MIMAT0003332	2.57E-15	1.19E-15	8.05	24.1	2.107	hsa-miR-656-3p
MIMAT0015001	1.45E-16	6.28E-17	8.41	27	2.097	hsa-miR-3135a
MIMAT0003339	1.43E-18	5.71E-19	8.96	31.7	2.096	hsa-miR-421
MIMAT0003284	4.19E-18	1.70E-18	8.84	30.6	2.096	hsa-miR-616-5p
MIMAT0000081	4.13E-17	1.76E-17	8.56	28.3	2.095	hsa-miR-25-3p
MIMAT0018939	8.61E-12	4.75E-12	6.94	16	2.095	hsa-miR-4424
MIMAT0026611	3.45E-19	1.34E-19	9.12	33.1	2.094	hsa-miR-520g-5p
MIMAT0021026	2.84E-24	8.55E-25	10.4	44.9	2.094	hsa-miR-5003-3p
MIMAT0019879	1.03E-17	4.25E-18	8.73	29.7	2.092	hsa-miR-4745-3p
MIMAT0018187	3.85E-22	1.28E-22	9.87	40	2.09	hsa-miR-3913-5p
MIMAT0023703	7.63E-20	2.85E-20	9.29	34.6	2.089	hsa-miR-6078
MIMAT0004497	4.21E-15	1.98E-15	7.99	23.6	2.089	hsa-miR-24-2-5p
MIMAT0004775	5.13E-17	2.19E-17	8.54	28.1	2.088	hsa-miR-502-3p
MIMAT0004599	1.86E-19	7.08E-20	9.19	33.7	2.088	hsa-miR-143-5p
MIMAT0001412	3.56E-12	1.93E-12	7.07	16.9	2.083	hsa-miR-18b-5p
MIMAT0021019	2.30E-24	6.86E-25	10.4	45.1	2.083	hsa-miR-5000-5p
MIMAT0000261	7.17E-15	3.40E-15	7.92	23.1	2.081	hsa-miR-183-5p
MIMAT0022292	1.57E-24	4.66E-25	10.4	45.5	2.072	hsa-miR-548au-3p
MIMAT0000086	5.95E-15	2.81E-15	7.94	23.3	2.071	hsa-miR-29a-3p
MIMAT0022923	6.21E-28	1.58E-28	11.2	53.4	2.07	hsa-miR-376b-5p
MIMAT0019894	8.07E-17	3.47E-17	8.48	27.6	2.068	hsa-miR-4754
MIMAT0005900	1.03E-17	4.27E-18	8.73	29.7	2.065	hsa-miR-1248
MIMAT0022281	2.51E-14	1.23E-14	7.75	21.8	2.062	hsa-miR-5583-5p
MIMAT0016850	8.94E-18	3.68E-18	8.75	29.8	2.062	hsa-miR-4301
MIMAT0000755	8.53E-14	4.30E-14	7.59	20.6	2.06	hsa-miR-323a-3p
MIMAT0005915	4.88E-19	1.91E-19	9.08	32.8	2.058	hsa-miR-1263
MIMAT0019067	4.04E-15	1.89E-15	7.99	23.7	2.057	hsa-miR-4528

MIMAT0004565	1.33E-13	6.79E-14	7.53	20.2	2.056	hsa-miR-218-1-3p
MIMAT0031006	1.17E-20	4.18E-21	9.5	36.5	2.056	hsa-miR-8079
MIMAT0003231	3.73E-15	1.74E-15	8	23.8	2.056	hsa-miR-567
MIMAT0018995	1.15E-19	4.36E-20	9.25	34.2	2.055	hsa-miR-4468
MIMAT0001541	7.29E-16	3.29E-16	8.21	25.4	2.055	hsa-miR-449a
MIMAT0007402	9.21E-20	3.47E-20	9.27	34.4	2.054	hsa-miR-103b
MIMAT0022701	5.85E-16	2.63E-16	8.24	25.6	2.052	hsa-miR-506-5p
MIMAT0024612	1.84E-23	5.77E-24	10.2	43	2.052	hsa-miR-378j
MIMAT0027608	1.74E-15	8.01E-16	8.1	24.5	2.052	hsa-miR-6854-5p
MIMAT0001620	9.15E-18	3.77E-18	8.74	29.8	2.051	hsa-miR-200a-5p
MIMAT0016880	2.83E-49	2.34E-50	15.2	103	2.051	hsa-miR-4259
MIMAT0000420	1.57E-18	6.29E-19	8.95	31.6	2.05	hsa-miR-30b-5p
MIMAT0028218	7.45E-19	2.94E-19	9.04	32.3	2.049	hsa-miR-7154-5p
MIMAT0000459	7.90E-14	3.97E-14	7.6	20.7	2.048	hsa-miR-193a-3p
MIMAT0000415	1.37E-15	6.25E-16	8.13	24.8	2.046	hsa-let-7i-5p
MIMAT0031015	6.19E-13	3.24E-13	7.32	18.6	2.044	hsa-miR-8088
MIMAT0002811	5.01E-16	2.25E-16	8.26	25.8	2.043	hsa-miR-202-3p
MIMAT0022692	8.45E-21	2.98E-21	9.54	36.9	2.041	hsa-miR-181b-3p
MIMAT0028233	1.17E-17	4.88E-18	8.71	29.6	2.039	hsa-miR-7161-3p
MIMAT0000707	5.44E-13	2.84E-13	7.34	18.8	2.037	hsa-miR-363-3p
MIMAT0016925	2.18E-21	7.49E-22	9.69	38.2	2.035	hsa-miR-500b-5p
MIMAT0019363	3.45E-12	1.87E-12	7.07	16.9	2.034	hsa-miR-3978
MIMAT0019776	1.17E-245	4.58E-249	37.3	560	2.033	hsa-miR-1343-3p
MIMAT0002832	6.71E-20	2.50E-20	9.31	34.8	2.032	hsa-miR-519c-3p
MIMAT0027410	6.16E-23	1.96E-23	10.1	41.8	2.03	hsa-miR-6755-5p
MIMAT0022501	4.62E-17	1.97E-17	8.55	28.2	2.028	hsa-miR-5707
MIMAT0005877	1.52E-16	6.60E-17	8.41	27	2.027	hsa-miR-1286
MIMAT0027607	3.77E-16	1.67E-16	8.29	26.1	2.026	hsa-miR-6853-3p
MIMAT0026617	9.23E-13	4.87E-13	7.26	18.2	2.023	hsa-miR-580-5p
MIMAT0018000	1.36E-14	6.58E-15	7.83	22.5	2.022	hsa-miR-23c
MIMAT0004570	3.65E-15	1.70E-15	8.01	23.8	2.022	hsa-miR-223-5p
MIMAT0019222	2.65E-16	1.17E-16	8.34	26.4	2.022	hsa-miR-3682-5p
MIMAT0007885	1.10E-14	5.28E-15	7.86	22.7	2.022	hsa-miR-1911-5p
MIMAT0027580	2.00E-34	3.53E-35	12.5	68.6	2.017	hsa-miR-6839-5p
MIMAT0024611	1.25E-22	4.02E-23	9.99	41.1	2.015	hsa-miR-6128
MIMAT0015000	2.66E-18	1.07E-18	8.89	31	2.014	hsa-miR-3134
MIMAT0002178	2.36E-14	1.15E-14	7.76	21.9	2.014	hsa-miR-487a-3p
MIMAT0015011	8.96E-15	4.27E-15	7.89	22.9	2.009	hsa-miR-3142
MIMAT0035703	6.68E-18	2.74E-18	8.78	30.1	2.008	hsa-miR-548bb-5p
MIMAT0019056	9.28E-16	4.21E-16	8.18	25.2	2.008	hsa-miR-4519
MIMAT0022702	2.18E-17	9.21E-18	8.64	28.9	2.005	hsa-miR-514a-5p
MIMAT0003215	3.36E-14	1.65E-14	7.72	21.6	2.004	hsa-miR-552-3p
MIMAT0019876	1.46E-11	8.13E-12	6.86	15.5	2.004	hsa-miR-3591-5p
MIMAT0019906	6.31E-15	2.98E-15	7.94	23.2	2.001	hsa-miR-4760-5p

GSE 106817 downregulated miRNAs

ID	adj.P.Val	P.Value	t	B	logFC	miRNA_ID_LIST
MIMAT0004602	9.08E-115	1.17E-116	-24	255	-3.28	hsa-miR-125a-3p
MIMAT0019015	4.73E-89	1.14E-90	-21	196	-3.1	hsa-miR-4481
MIMAT0019710	6.04E-106	9.41E-108	-23	235	-2.97	hsa-miR-4648
MIMAT0004592	4.61E-31	9.75E-32	-12	60.8	-2.73	hsa-miR-125b-1-3p
MIMAT0026554	2.25E-34	3.99E-35	-13	68.5	-2.72	hsa-miR-433-5p
MIMAT0024610	5.34E-94	1.04E-95	-22	207	-2.53	hsa-miR-6127
MIMAT0019852	4.79E-110	7.1E-112	-24	244	-2.48	hsa-miR-4730
MIMAT0027363	2.91E-83	7.83E-85	-20	182	-2.32	hsa-miR-6731-5p
MIMAT0018164	5.74E-33	1.11E-33	-12	65.2	-2.32	hsa-miR-3713
MIMAT0027614	6.18E-73	2.05E-74	-19	159	-2.3	hsa-miR-6857-5p
MIMAT0025458	3.6E-36	5.81E-37	-13	72.7	-2.29	hsa-miR-6501-5p

MIMAT0015064	3.67E-141	2.72E-143	-27	317	-2.28	hsa-miR-3184-5p
MIMAT0015017	3.71E-54	2.44E-55	-16	115	-2.26	hsa-miR-1273c
MIMAT0003240	7.65E-141	5.96E-143	-27	316	-2.25	hsa-miR-575
MIMAT0018121	6.11E-31	1.3E-31	-12	60.5	-2.18	hsa-miR-3692-5p
MIMAT0004589	4.2E-46	4.11E-47	-15	95.9	-2.12	hsa-miR-30b-3p
MIMAT0011778	9.26E-27	2.55E-27	-11	50.7	-2.11	hsa-miR-2278
MIMAT0027402	2.97E-35	4.97E-36	-13	70.6	-2.05	hsa-miR-6751-5p
MIMAT0027666	1.2E-22	3.86E-23	-10	41.2	-2.05	hsa-miR-6883-5p

GSE 59856 upregulated miRNAs

ID	adj.P.Val	P.Value	t	B	logFC	miRNA_ID_LIST
MIMAT0019964	2.05E-29	1.2E-31	13.5	61.2	1.08	hsa-miR-4792
MIMAT0023700	2.59E-48	4.05E-51	19.2	106	1.03	hsa-miR-6075
MIMAT0005898	1.87E-14	5.85E-16	8.67	25.4	1.01	hsa-miR-1246
MIMAT0020603	3.66E-15	1.1E-16	8.91	27.1	0.988	hsa-miR-5096
MIMAT0005880	1.85E-12	7.16E-14	7.94	20.7	0.959	hsa-miR-1290
MIMAT0022286	1.22E-21	1.86E-23	11.1	42.5	0.954	hsa-miR-5585-3p
MIMAT0027574	1.82E-26	1.57E-28	12.6	54.1	0.873	hsa-miR-6836-5p
MIMAT0019855	1.14E-14	3.53E-16	8.74	25.9	0.868	hsa-miR-4732-5p
MIMAT0005876	1.8E-13	6.06E-15	8.32	23.1	0.816	hsa-miR-1285-3p
MIMAT0026622	2.4E-17	5.55E-19	9.67	32.3	0.814	hsa-miR-619-5p

GSE 59856 downregulated miRNAs

ID	adj.P.Val	P.Value	t	B	logFC	miRNA_ID_LIST
MIMAT0004602	3.14E-71	1.23E-74	-27	159	-2.47	hsa-miR-125a-3p
MIMAT0022693	4.84E-42	1.14E-44	-17	90.9	-1.63	hsa-miR-204-3p
MIMAT0004592	2.76E-52	3.24E-55	-21	115	-1.62	hsa-miR-125b-1-3p
MIMAT0019003	3.48E-36	1.36E-38	-16	77.1	-1.57	hsa-miR-4476
MIMAT0018971	2.48E-28	1.65E-30	-13	58.6	-1.47	hsa-miR-4450
MIMAT0029782	3.32E-26	3.25E-28	-13	53.4	-1.42	hsa-miR-7641
MIMAT0003240	9.65E-40	3.02E-42	-17	85.4	-1.41	hsa-miR-575
MIMAT0027686	1.57E-63	1.23E-66	-24	141	-1.36	hsa-miR-6893-5p
MIMAT0027431	7.18E-32	3.37E-34	-14	67	-1.23	hsa-miR-6765-3p
MIMAT0003228	3.28E-19	6.29E-21	-10	36.7	-1.12	hsa-miR-564
